# Ti-Doping in Silica-Supported PtZn Propane Dehydrogenation Catalysts: From Improved Stability to the Nature of the Pt–Ti Interaction

**DOI:** 10.1021/jacsau.3c00197

**Published:** 2023-06-30

**Authors:** Lukas Rochlitz, Jörg W. A. Fischer, Quentin Pessemesse, Adam H. Clark, Anton Ashuiev, Daniel Klose, Pierre-Adrien Payard, Gunnar Jeschke, Christophe Copéret

**Affiliations:** † Department of Chemistry and Applied Biosciences, ETH Zürich, Vladimir-Prelog-Weg 2, Zürich CH-8093, Switzerland; ‡ Université de Lyon, Université Claude Bernard Lyon I, CNRS, INSA, CPE, UMR 5246, ICBMS, Rue Victor Grignard, Villeurbanne Cedex F-69622, France; § Paul Scherrer Institut, Villigen CH-5232, Switzerland

**Keywords:** propane dehydrogenation, metal−support interaction, heterogeneous catalysis, alloys, nanoparticles, regeneration, interfaces, promoters

## Abstract

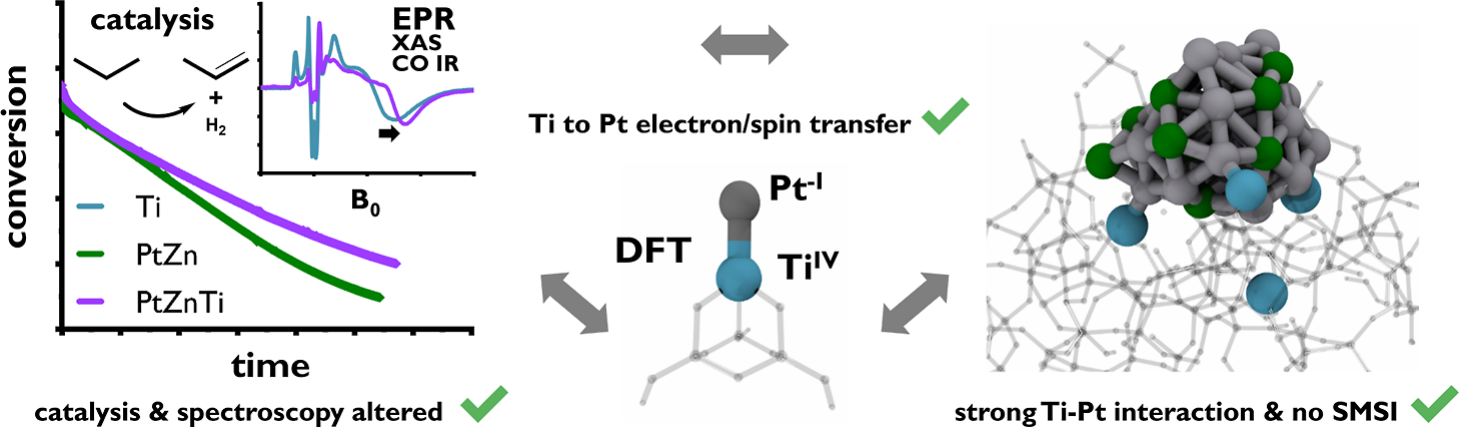

Propane dehydrogenation is an important industrial reaction to access propene, the world’s second most used polymer precursor. Catalysts for this transformation are required to be long living at high temperature and robust toward harsh oxidative regeneration conditions. In this work, combining surface organometallic chemistry and thermolytic molecular precursor approach, we prepared well-defined silica-supported Pt and alloyed PtZn materials to investigate the effect of Ti-doping on catalytic performances. Chemisorption experiments and density functional calculations reveal a significant change in the electronic structure of the nanoparticles (NPs) due to the Ti-doping. Evaluation of the resulting materials PtZn/SiO_2_ and PtZnTi/SiO_2_ during long deactivation phases reveal a stabilizing effect of Ti in PtZnTi/SiO_2_ with a *k*_d_ of 0.015 h^–1^ compared to PtZn/SiO_2_ with a *k*_d_ of 0.022 h^–1^ over 108 h on stream. Such a stabilizing effect is also present during a second deactivation phase after applying a regeneration protocol to the materials under O_2_ and H_2_ at high temperatures. A combined scanning transmission electron microscopy, *in situ* X-ray absorption spectroscopy, electron paramagnetic resonance, and density functional theory study reveals that this effect is related to a sintering prevention of the alloyed PtZn NPs in PtZnTi/SiO_2_ due to a strong interaction of the NPs with Ti sites. However, in contrast to classical strong metal–support interaction, we show that the coverage of the Pt NPs with TiO_*x*_ species is not needed to explain the changes in adsorption and reactivity properties. Indeed, the interaction of the Pt NPs with Ti^III^ sites is enough to decrease CO adsorption and to induce a red-shift of the CO band because of electron transfer from the Ti^III^ sites to Pt^0^.

## 1 Introduction

Heterogeneous catalysts heavily rely on using multi-metallic materials, where different elements (promoters) have been introduced during the catalyst development phase to improve their catalytic performances. A prominent example is propane dehydrogenation (PDH) utilizing Pt-based catalysts, containing several promoters to improve the catalytic performances, namely, catalyst selectivity, stability, and regenerability. Improving PDH catalysts has recently gained momentum because PDH has become a strategically important process in the petrochemical industry due to the emergence of shale gas resources and the changes in the cracking technology impairing propene production from such a source.^1−3^ The main challenges associated with PDH are related to the endothermic nature of this reaction (Δ*H*_298_^0^ = 124.3 kJ mol^–1^), resulting in the use of high operating temperatures (550–750 °C) to achieve reasonable conversion levels. These temperatures favor coke formation and result in increased catalyst sintering, both leading to deactivation.

The Oleflex process, developed in the 1990s, was the first industrial process for PDH based on a bimetallic catalyst, namely, PtSn supported on Al_2_O_3_. Much more recently, another bimetallic, PtGa-based catalyst was implemented in industrial settings.^1,4−6^ Several recent reviews furthermore highlight the high relevance of this process.^3,7−9^ Overall, many different metal promoters have been utilized to improve the catalytic performances of Pt-based systems for light alkane dehydrogenation; most of them being post-transition (Zn,^10−15^ Ga,^4,16^ In,^17^ and Sn^1,18,19^) and transition metals (Mn^20−22^ or Cu^23−27^). Besides these promoters, alkali metals and different supports have been used to improve catalyst performances, with the goal to minimize cracking and improve the regeneration process.^1^

In that context, late and post transition-metal promoters are known to form alloyed phases with Pt facilitating propene desorption and preventing coke deposition, hence the improved selectivity and stability. The reduced coke formation was proposed to result from Pt site isolation at the alloy surface, while the facilitated propene desorption likely results from several factors including electronic effects of the promoters.^1^ Sintering is further proposed to be prevented by the strong interaction of oxidized surface sites with the catalytically active metal phase, resulting in increased stability.^1^ In most cases, the role of promoters remains to be understood at the molecular level, in particular for early- and mid-transition metals. For instance, while ordered PtMn alloys have been proposed to form, leading to similar effects as shown for post transition-metal containing systems,^20^ evidence for highly segregated PtMn structures with no Pt site isolation have also been reported.^22^ Moving further to the left of the periodic table, noteworthy examples are based on Pt nanoparticles (NPs) supported on aluminum titanate that display improved catalytic performances compared to monometallic Pt; this has been attributed to Ti improving propene desorption and facilitating coke migration to the support surface.^28^ While being more difficult to reduce than Mn, Ti is well-known to alter the reactivity of Pt NPs when supported on TiO_2_ due to the so-called strong metal–support interaction (SMSI).^29,30^ More recent literature shows that the interaction of Pt and TiO_2_ can be of different nature depending on factors like particle size, specific treatments of the materials, as well as treatment temperature.^31−34^ This strong interaction can result in migration of Ti species on the Pt NP surface, which can further lead to its encapsulation. Ongoing scientific discussions, related to the nature of possible interactions between Pt and Ti species, highlight the need to further investigate Pt and Ti containing materials in order to develop a better molecular-level understanding of these systems.

Our group has recently introduced a combined methodology of surface organometallic chemistry (SOMC)^35−38^ and thermolytic molecular precursor (TMP)^39^ approach to generate multi-metallic materials with tailored interfaces and compositions (*e.g.*, alloys).^40,41^ This approach is particularly well-suited to obtain a molecular-level understanding of promotional effects in heterogeneous catalysts by enabling the acquisition of detailed and conclusive information from X-ray absorption spectroscopy (XAS), CO adsorption followed by Fourier-transform infrared (FTIR) spectroscopy, high-angle annular dark-field scanning transmission electron microscopy (HAADF-STEM), and computational studies.^10,16,22,42^ As an example, our group has recently reported that the presence of isolated, Lewis acidic Ti^IV^ sites at the interface of Cu NPs enables promotion of methanol formation rates in the CO_2_ hydrogenation reaction when supported on silica doped with surface Ti sites. This result sharply contrasts the reactivity of Cu supported on TiO_2_ that displays a much lower activity and forms mostly CO.^43,44^

In this work, we decided to investigate the effect of Ti sites on Pt-based materials and to also discuss the results in the context of PDH, with the goal to understand the promotional effect of Ti. For this reason, we prepared and studied, both, monometallic Pt NPs supported on SiO_2_ and an earlier reported bimetallic PtZn material—known to be a promising PDH catalyst^10,14,45^—and their Ti-doped equivalents *via* a SOMC/TMP approach. A significant sintering prevention can be observed for Ti-doped materials, leading to higher stability under PDH conditions compared to Ti-free catalysts. A combination of STEM/EDX, CO adsorption followed by FTIR and catalytic PDH tests is first used to identify and describe similarities and differences between the investigated materials. In the second step, we further use a more sophisticated approach, combining a CO/H_2_ chemisorption, XAS, electron paramagnetic resonance (EPR), and a density functional theory (DFT)-based computational study, in order to better understand the role of Ti. We identify the presence of a strong interaction between Pt NPs and Ti surface sites, which could explain the increased stability of Ti-doped materials during PDH. We further identify that such an interaction is particularly strong for Ti^III^ d^1^ sites with Pt^0^, inducing an electron transfer associated with the formation of a Pt^δ−^-Ti^IV^ system, leading to a change in the electronic structure of Pt NPs and adsorption properties of Pt surface atoms, and also increased sintering resistance during PDH.

## 2 Results and Discussion

### 2.1 Synthesis, STEM, and CO Adsorption FTIR Studies

**Figure 1:**
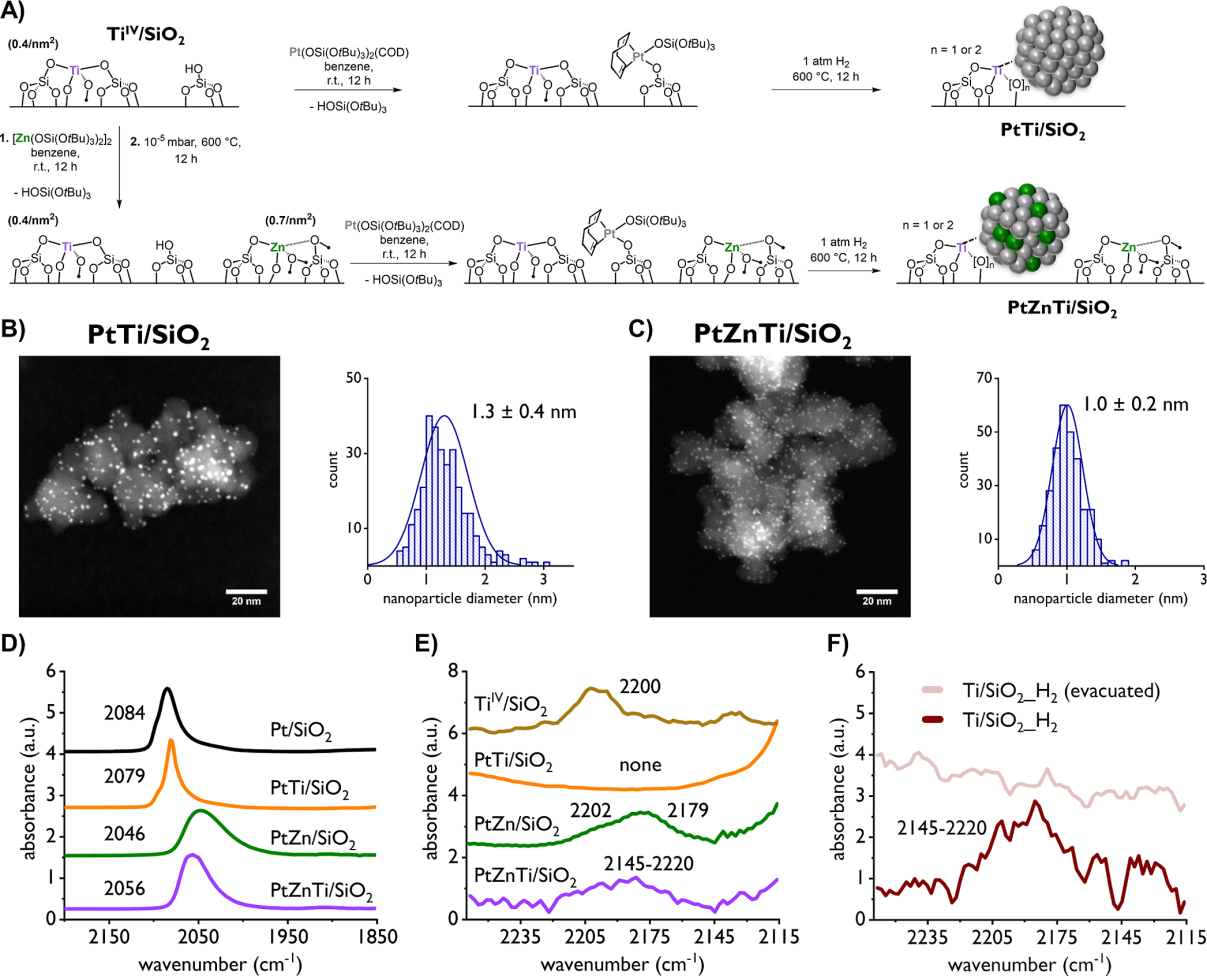
(A) Schematic representation of the synthetic approach used for PtTi/SiO_2_ and PtZnTi/SiO_2_ resulting in Pt NPs on a Ti-doped SiO_2_ support and PtZn-alloyed NPs on a Ti/Zn-doped SiO_2_ support, respectively. (B) Representative HAADF-STEM micrograph of PtTi/SiO_2_ and corresponding PSD. (C) Representative HAADF-STEM micrograph of PtZnTi/SiO_2_ and corresponding PSD. (D) Background subtracted FTIR spectra of adsorbed ^12^CO on several Pt containing materials showing the adsorption feature attributed to CO being adsorbed on Pt. (E) Background subtracted FTIR spectra of adsorbed ^12^CO on several materials showing the adsorption feature(s) attributed to CO being adsorbed on Lewis acidic sites. (F) Background subtracted ^12^CO adsorption FTIR spectra of Ti/SiO_2__H_2_, a Ti containing material that was treated under H_2_ (see the text for more detail), before and after evacuation under high vacuum showing the adsorption feature attributed to CO adsorption on Lewis acidic sites.

To investigate the effect of Ti on the catalytic performances of Pt-based materials for the PDH reaction, we prepared a trimetallic silica-supported Pt–Zn–Ti material, the corresponding bimetallic Pt–Ti and Pt–Zn, and the monometallic Pt ones using the SOMC/TMP approach (Figure 1A).^10,16,39^ Highly dispersed Ti^IV^ sites were introduced on SiO_2_ by grafting [Ti^IV^(OSi(OtBu)_3_)_3_(O^i^Pr)] on SiO_2-700_ (see the Supporting Information for experimental details) followed by a thermal treatment under high vacuum.^44^ The resulting Ti^IV^/SiO_2_ material contains Ti^IV^ sites (∼0.4 Ti/nm^2^, ∼0.6 wt % Ti) along with isolated surface OH groups (compare Figure S1). In order to obtain Ti/SiO_2__H_2_, Ti^IV^/SiO_2_ was treated under H_2_ at 600 °C for 10 h. The introduction of Zn for the trimetallic case was carried out by further grafting [Zn^II^(OSi(OtBu)_3_)_2_]_2_ on Ti^IV^/SiO_2_ (0.3 mmol/(g Ti^IV^/SiO_2_) nominal grafting) followed by a subsequent treatment under high vacuum at 600 °C (see the Supporting Information for experimental details) to obtain Zn^II^Ti^IV^/SiO_2_.

With these supports in hand, Pt was introduced by grafting [Pt^II^(OSi(OtBu)_3_)_2_(COD)] (0.3 mmol Pt *per* g Ti^IV^/SiO_2_ or Zn^II^Ti^IV^/SiO_2_)^46,47^ (COD = 1,5-cyclooctadiene) onto the surface OH groups of Ti^IV^/SiO_2_ or Zn^II^Ti^IV^/SiO_2_. The resulting materials—Pt^II^_Ti^IV^/SiO_2_ and Pt^II^_Zn^II^Ti^IV^/SiO_2_—were then introduced to a flow of H_2_ at 600 °C, leading to the formation of PtTi/SiO_2_ and PtZnTi/SiO_2_, respectively. The final materials were all black solids (3.46 wt % Pt, 0.59 wt % Ti and 2.95 wt % Pt, 1.53 wt % Zn, 0.58 wt % Ti), indicating NP formation. Reference materials Pt/SiO_2_ and PtZn/SiO_2_ were also synthesized in a similar way as reported earlier.^10^ HAADF-STEM micrographs of the PtTi/SiO_2_ and PtZnTi/SiO_2_ materials show the formation of rather small and homogeneously distributed NPs with narrow particle size distribution (PSD) (Figure 1B,C and Table 1).

**Table 1:** EA, PSD, and CO Adsorption FTIR and Chemisorption Results for a Collection of Materials

	metal loadings EA [wt %]					CO adsorption FTIR [cm^–1^]		chemisorption [mmol X/g_Pt_]	
material	Pt	Zn	Ti	PSD [nm]	PSD after (PDH) [nm]	Pt	LA site(s)	CO	H_2_
Ti^IV^/SiO_2_			0.59				2200		
Ti/SiO_2__H2			0.59				2145–2220		
Zn^II^/SiO_2_^a^		1.73					2206		
Pt/SiO_2_^b^	3.96			2.0 ± 0.8	2.5 ± 0.9 (10 h)	2084		2.5^c^	1.7^c^
PtTi/SiO_2_	3.46		0.59	1.3 ± 0.4	1.5 ± 0.6 (19 h)	2079		2.7	1.4
PtZn/SiO_2_^d^	3.13	1.63		1.0 ± 0.3	1.3 ± 0.4 (109 h)	2046	2179/2202	1.7	1.5
PtZnTi/SiO_2_	2.95	1.53	0.58	1.0 ± 0.2	1.1 ± 0.3 (114 h)	2056	2145–2220	1.5	1.2

a Data from ref (10).

b Data from ref (22) unless otherwise indicated.

c Data from ref (16).

d CO FTIR and chemisorption data from ref (10).

Notably, PtTi/SiO_2_ displays significantly smaller particles with narrower PSD (1.3 ± 0.4 nm) than monometallic Pt/SiO_2_ (2.0 ± 0.8 nm), while the bi- and trimetallic PtZn/SiO_2_ (1.0 ± 0.3 nm) and PtZnTi/SiO_2_ (1.0 ± 0.2 nm) have the same PSD, slightly smaller than what is observed for PtTi/SiO_2_ (Table 1). HAADF-STEM analysis indicates an effect of Ti on the particle formation, which results in smaller particle sizes and a narrower PSD, especially in the absence of Zn (see the Supporting Information for additional STEM micrographs and size distributions).

To obtain further information about the surface structure of the various supported NPs, CO adsorption FTIR spectra of PtTi/SiO_2_ and PtZnTi/SiO_2_ were then recorded by exposing self-supporting pellets of the materials to CO (around 11 mg, 10 mbar CO) and compared to the spectra obtained for Pt/SiO_2_, PtZn/SiO_2_, and materials without Pt, namely, Ti^IV^/SiO_2_, Ti/SiO_2__H_2_, and Zn^II^/SiO_2_. The results are summarized in Figure 1D–F and Table 1. As seen from Figure 1D, PtTi/SiO_2_ shows a feature at 2079 cm^–1^ close to what is observed for Pt/SiO_2_ (2084 cm^–1^), albeit slightly red-shifted, characteristic of pure Pt NPs. In contrast, PtZn/SiO_2_ (2046 cm^–1^) and PtZnTi/SiO_2_ (2056 cm^–1^) both show strongly red-shifted FTIR CO vibrational frequencies compared to Pt NPs, consistent with PtZn alloy formation.^10^ While the vibrational bands of these sites are broad, indicating the existence of multiple adsorption sites, the small difference (*ca.* 10 cm^–1^) could be related to slight changes in the NP surface structure (composition) induced by the presence of Ti at the interface. Figure 1E shows that Ti^IV^/SiO_2_, Zn^II^/SiO_2_, and PtZnTi/SiO_2_ all have features close to 2200 cm^–1^, related to CO adsorbed on Lewis acidic sites, while no such feature is observed for PtTi/SiO_2_, indicating that Ti sites in PtTi/SiO_2_ are not accessible for CO adsorption. A similar feature in PtZnTi/SiO_2_ and PtZn/SiO_2_ makes it reasonable to assume that both originate from CO adsorbed on Zn^II^ sites^10^ (Figure 1E) while some adsorption on Ti sites cannot be entirely excluded as CO adsorbed on Ti/SiO_2__H_2_ also shows a similar feature (Figure 1F).

### 2.2 Catalytic Evaluation in Propane Dehydrogenation

**Figure 2:**
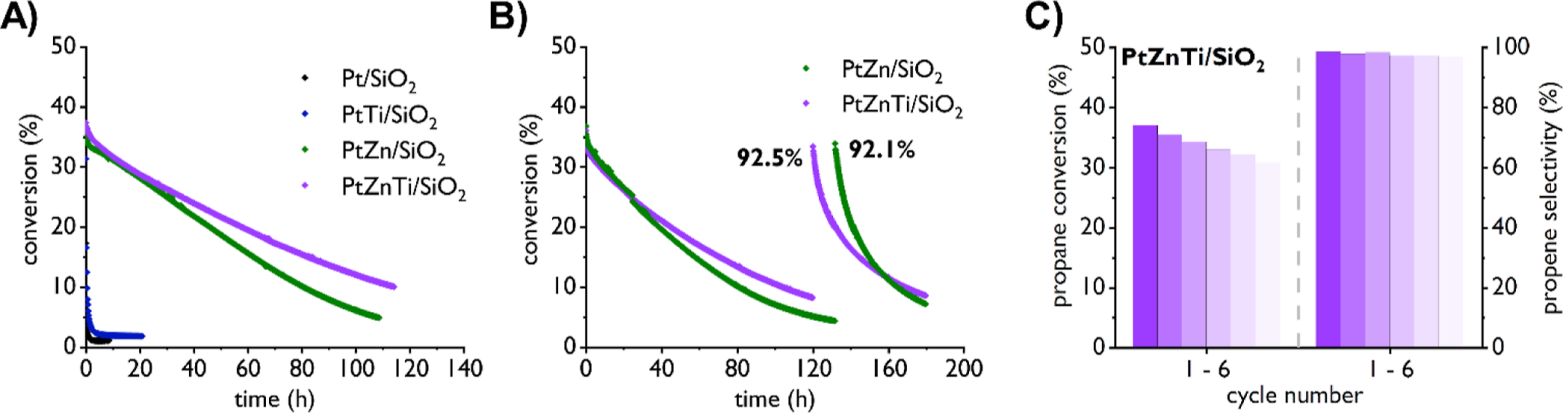
(A) Propane conversion of several Pt containing materials indicating an effect of Ti-doping on the stability of Pt and PtZn NPs supported on SiO_2_. (B) Propane conversion of PtZn/SiO_2_ and PtZnTi/SiO_2_ during two consecutive long deactivation phases with an intermediate long regeneration cycle (1.5 h O_2_ and 1 h H_2_; see the Supporting Information and Table 3 for detail), indicating a stabilizing effect of Ti also after regeneration conditions were applied. (C) Propane conversion (left) and propene selectivity (right) for PtZnTi/SiO_2_ during six consecutive fast deactivation/regeneration cycles (20 min PDH, 20 min O_2_, and 20 min H_2_; see the Supporting Information and Table 3 for detail) showing rather linear deactivation of the catalyst under such conditions while the propene selectivity stays at a very high level.

Next, we evaluated the catalytic performance of these materials in PDH using similar conditions in all cases. The catalytic results are summarized in Table 2 and Figure 2A. PtTi/SiO_2_ shows increased initial and final productivity compared to Pt/SiO_2_ even when run at more than doubled weight hourly space velocity (WHSV) while selectivity levels are very comparable to the monometallic material. This is consistent with a lower deactivation constant of PtTi/SiO_2_ (*k*_d_: 1.33 h^–1^) compared to Pt/SiO_2_ (*k*_d_: 1.46 h^–1^), despite the considerably higher WHSV for the bimetallic material. The observed differences could at least in part be due to the significantly smaller particles in PtTi/SiO_2_ (initial: 1.3 ± 0.4 nm; final: 1.5 ± 0.6 nm; see Table 1) compared to Pt/SiO_2_ (initial: 2.0 ± 0.8 nm; final: 2.5 ± 0.9 nm; see Table 1). However, besides the general differences in particle size, the HAADF-STEM data (for additional STEM micrographs and PSDs, see the Supporting Information) indicate a significant sintering reduction of the bimetallic PtTi compared to the monometallic material that can be related to the presence of Ti sites. As a control experiment, we also tested Ti/SiO_2__H_2_ which proved inactive under the applied catalytic conditions (see Figure S53).

**Table 2:** Catalytic PDH Parameters of Several Materials at 550 °C and 10:40 mL/min (C_3_H_8_/Ar) at 1 barg

material	time [h]	conv. [%]	select. [%]	WHSV [g_C3H8_/g_Pt_ h]	productivity [g_C3H6_/g_Pt_ h]	*k*_d_ [h^–1^]
Pt/SiO_2_^a^	0.1	17.2	81.5	834	110	1.46
	0.2	1.1	77.8		7	
PtTi/SiO_2_	0.1	31.3	77.7	1916	445	1.33
	2	3.1	82.1		47	
PtZn/SiO_2_	0.1	36.7	98.7	2067	714	0.022
	108	4.9	90.0		87	
PtZnTi/SiO_2_	0.1	37.3	98.4	2233	782	0.015
	108	10.8	95.3		220	

a All catalytic data for this sample are taken from ref (22).

We next evaluated whether the Ti addition also has an effect on the catalytic performance and regeneration behavior of SiO_2_-supported bimetallic, alloyed PtZn NPs. We first tested PtZnTi/SiO_2_ in comparison with PtZn/SiO_2_ under the same catalytic conditions as we applied for PtTi/SiO_2_. As can be seen from Table 2, the initial productivities of PtZn/SiO_2_ and PtZnTi/SiO_2_ are very similar. However, after 108 h on stream, the productivity of the trimetallic material remains much higher (220 g_C3H6_/g_Pt_ h) compared to the PtZn material after 108 h on stream (87 g_C3H6_/g_Pt_ h); clearly indicating a stabilizing effect of Ti also in the case of alloyed PtZn particles supported on SiO_2_. The stabilizing effect is also apparent from a comparison of the deactivation constants of PtZnTi/SiO_2_ (0.015 h^–1^) compared to PtZn/SiO_2_ (0.022 h^–1^). Interestingly, the effect on deactivation starts to be clearly visible only after around 30 h on stream which could be due to compounding effects associated with the increase of particle sizes and coke formation (Figure 2A). The increase in stability can again most likely be attributed to sintering prevention as reflected in the PSD of PtZnTi/SiO_2_ (initial: 1.0 ± 0.2 nm; final: 1.1 ± 0.3 nm; see Table 1) compared to PtZn/SiO_2_ (initial: 1.0 ± 0.3 nm; final: 1.3 ± 0.4 nm; see Table 1).

With this information in hand, we were then interested to investigate whether the stabilizing effect of Ti species is still present after applying regeneration conditions. The results are summarized in Figure 2B and Table 3. We applied two long deactivation phases to the PtZn and PtZnTi materials with a long regeneration phase in between the two deactivation phases consisting of a 90 min O_2_/Ar and a consecutive 60 min H_2_ treatment (see footnote of Table 3 for details). The data show that during the second deactivation phase, the stabilizing effect due to Ti remains. The deactivation rate for PtZn/SiO_2_ (*k*_d_: 0.040 h^–1^) is still higher during the second phase than that for PtZnTi/SiO_2_ (*k*_d_: 0.028 h^–1^), while both materials generally deactivate faster compared to the first deactivation phase. The effect of Ti is again likely attributed to sintering prevention, as indicated by the different PSD of PtZn/SiO_2_ (1.5 ± 0.8 nm) compared to PtZnTi/SiO_2_ (1.2 ± 0.7 nm) after a total of three long deactivation phases (see footnote of Table 3 for detail).

**Table 3:** Regeneration Data for the Short and Long Regeneration Cycles of PtZn/SiO_2_ and PtZnTi/SiO_2_

material	regeneration cycle	conv. [%]^a^	select. [%]^a^	*k*_d_ [h^–1^]^b^	PSD^c^
Short Cycles^d^					
PtZn/SiO_2_	1	37.8	98.3	0.151	1.0 ± 0.3
	6	31.0	97.0		1.7 ± 1.2
PtZnTi/SiO_2_	1	37.0	98.5	0.139	1.0 ± 0.3
	6	30.8	96.9		1.5 ± 1.0
Long Cycles^e^					
PtZn/SiO_2_	1	36.8	98.0	0.019	1.0 ± 0.3
	2	33.9	95.0	0.040	1.5 ± 0.8^f^
PtZnTi/SiO_2_	1	36.0	98.3	0.015	1.0 ± 0.3
	2	33.4	95.7	0.028	1.2 ± 0.7^f^

a Average of 20 min (three datapoints) for short regeneration cycles; initial values for long regeneration cycles.

b Calculated for the initial conversion of first to sixth cycle and a total of 2 h on stream for short cycles; calculated for the initial conversion of cycle X to the final conversion of the same cycle for long regeneration cycles.

c Before the first cycle and after second/sixth cycle.

d PDH cycles of 20 min with 10:40 mL/min (C_3_H_8_:Ar) at 550 °C and 1 barg [WHSV of 1079 h^–1^ (PtZn/SiO_2_) and 1134 h^–1^ (PtZnTi/SiO_2_)] with flow of 5% O_2_/Ar at 500 °C and 0.5 barg for 20 min and 100% H_2_ at 550 °C and 0.5 barg for 20 min in between deactivation phases.

e PDH cycles of several days (see Figure 2B) with 10:40 mL/min (C_3_H_8_:Ar) at 550 °C and 1 barg [WHSV of 2105 h^–1^ (PtZn/SiO_2_) and 2220 h^–1^ (PtZnTi/SiO_2_)] with flow of 5% O_2_/Ar at 500 °C and 0.5 barg for 90 min and 100% H_2_ at 550 °C and 0.5 barg for 60 min in between deactivation phases.

f The values shown are after three long cycles and an overall of 315.1 h (PtZn/SiO_2_) and 221.3 h (PtZnTi/SiO_2_) on stream.

We next evaluated the stability of PtZn/SiO_2_ and PtZnTi/SiO_2_ during fast, consecutive deactivation/regeneration conditions as frequently applied in industrial settings. For this purpose, we tested both the PtZn/SiO_2_ and PtZnTi/SiO_2_ material under similar conditions, consisting of cycles of 20 min PDH, followed by 20 min oxidation and reduction treatments each (see the Supporting Information and Table 3 for details). The results are depicted in Figure 2C and Table 3 (see Figure S59 for the corresponding results for PtZn/SiO_2_). In both cases, the deactivation over six consecutive regeneration cycles follows a linear and very similar trend for both materials with very minor stabilization of conversion levels. In contrast to the significantly decreasing conversion levels, propene selectivity maintains a very high level in both cases (≥97%). While the deactivation constant is slightly lower for the trimetallic (*k*_d_: 0.139 h^–1^) compared to the bimetallic (*k*_d_: 0.151 h^–1^) case, both materials suffer from significant deactivation over the six regeneration cycles. This observation can most likely be related to the more distinct particle growth over the total of 2 h on stream during the six short regeneration cycles (+0.7 nm for PtZn/SiO_2_; +0.5 nm for PtZnTi/SiO_2_, see also Table 3) compared to the one observed after more than 100 h on stream during the long PDH deactivation phase (*vide supra*; +0.3 nm for PtZn/SiO_2_; +0.1 nm for PtZnTi/SiO_2_). The combined results clearly indicate that the particle growth during regeneration cycles is not due to the applied PDH conditions but must be a result of sintering during the O_2_/Ar and H_2_ treatments. In summary, these data show that Ti does not exhibit a significant stabilizing effect on the PtZn particles supported on SiO_2_ for a small number of fast, consecutive deactivation/regeneration phases while a clear stabilizing effect can be observed for both, long deactivation phases before and after regeneration.

### 2.3 Experimental Indication of Pt–Ti Interactions

Preliminary characterization data for the Ti containing materials PtTi/SiO_2_ as well as PtZnTi/SiO_2_ indicate that there is no significant difference between the Ti-doped materials and Pt/SiO_2_ and PtZn/SiO_2_, respectively. Besides, the PSD for Pt/PtTi with a significant difference in PSD, CO adsorption FTIR analyses, and PSDs of the materials are similar compared to the respective materials without Ti addition, indicating that Ti does not alloy with Pt or PtZn NPs. In contrast, catalytic tests in the PDH reaction, as well as regeneration studies, gave a first clear indication that Ti addition leads to a significant stabilizing effect under catalytic conditions. We therefore expected some kind of NP interaction with Ti sites, most likely a Pt–Ti interaction. Such an interaction was already hinted at by the non-accessibility of Ti sites for CO adsorption, observed in the CO FTIR study. In the following sections, we are aiming at deciphering this interaction with combined chemisorption, XAS, EPR, and computational studies in order to explain the behavior of the materials under catalytic conditions.

#### 2.3.1 CO and H_2_ Adsorption Analysis

As a first step, we performed H_2_ and CO chemisorption on PtTi/SiO_2_ (2.7 mmol CO/g_Pt_, 1.4 mmol H_2_/g_Pt_) and PtZnTi/SiO_2_ (1.5 mmol CO/g_Pt_, 1.2 mmol H_2_/g_Pt_) and compared these two materials to the earlier reported Pt/SiO_2_ (2.5 mmol CO/g_Pt_, 1.7 mmol H_2_/g_Pt_)^16^ and PtZn/SiO_2_ (1.7 mmol CO/g_Pt_, 1.5 mmol H_2_/g_Pt_)^10^ in order to obtain a better understanding of the surface structure/composition (see Table 1). At first glance, the data show that a large drop in CO adsorption ability occurs when Zn is present, while the H_2_ adsorption ability is less affected. Additionally, when comparing the adsorption of H_2_ and CO, the CO/H_2_ adsorption ratio is significantly higher for PtTi/SiO_2_ (1.93) and drops considerably for Pt/SiO_2_ (1.47), while they are similar yet lower for PtZn/SiO_2_ (1.13) and PtZnTi/SiO_2_ (1.25). Looking at the data in more detail, one can observe a significant decrease in CO and H_2_ adsorption ability when going from Pt/SiO_2_ to PtTi/SiO_2_. This decrease is especially obvious, when considering the large difference in PSD (1.3 nm for PtTi/SiO_2_*vs* 2.0 nm for Pt/SiO_2_) that indicates much larger reactive surface for the bimetallic material, which should result in considerably higher CO and H_2_ adsorption values; however, this is not the case (see Table 1). The data indicate that Ti greatly decreases the adsorption ability of Pt NPs, indicating that there must be some close proximity between Pt and Ti,^48^ even if the strength of the CO bond itself is only weakly affected as evidenced by FTIR spectroscopy (*vide supra*).

We already reported that moving from Pt/SiO_2_ to PtZn/SiO_2_ materials (with large difference in PSD) results in a significant drop in both the CO and H_2_ adsorption ability, although with a much stronger decrease for CO. A comparison of PtZn/SiO_2_ with PtTi/SiO_2_ furthermore reveals that Zn introduction leads to an even stronger decrease in CO and H_2_ adsorption (note the 0.3 nm PSD difference) with a much stronger effect on the CO adsorption ability. PtZn/SiO_2_ and PtZnTi/SiO_2_ show a rather similar drop in adsorption with a slightly decreased adsorption ability for PtZnTi/SiO_2_ for both probe molecules, which can be attributed to a combined effect of Zn and Ti-doping.

In summary, the data show that in both cases of Ti-doping, a significant decrease in CO and H_2_ adsorption ability of the material can be detected, similar to what is observed upon SMSI, while CO adsorption FTIR shows that no significant coverage of the particles with TiO_*x*_ species is taking place. The data indicate that Ti must be in close proximity to the Pt and PtZn particles while not significantly covering their surface (see the Supporting Information for more detail and H_2_ and CO uptake curves).

#### 2.3.2 X-ray Absorption Spectroscopy Analysis

**Figure 3:**
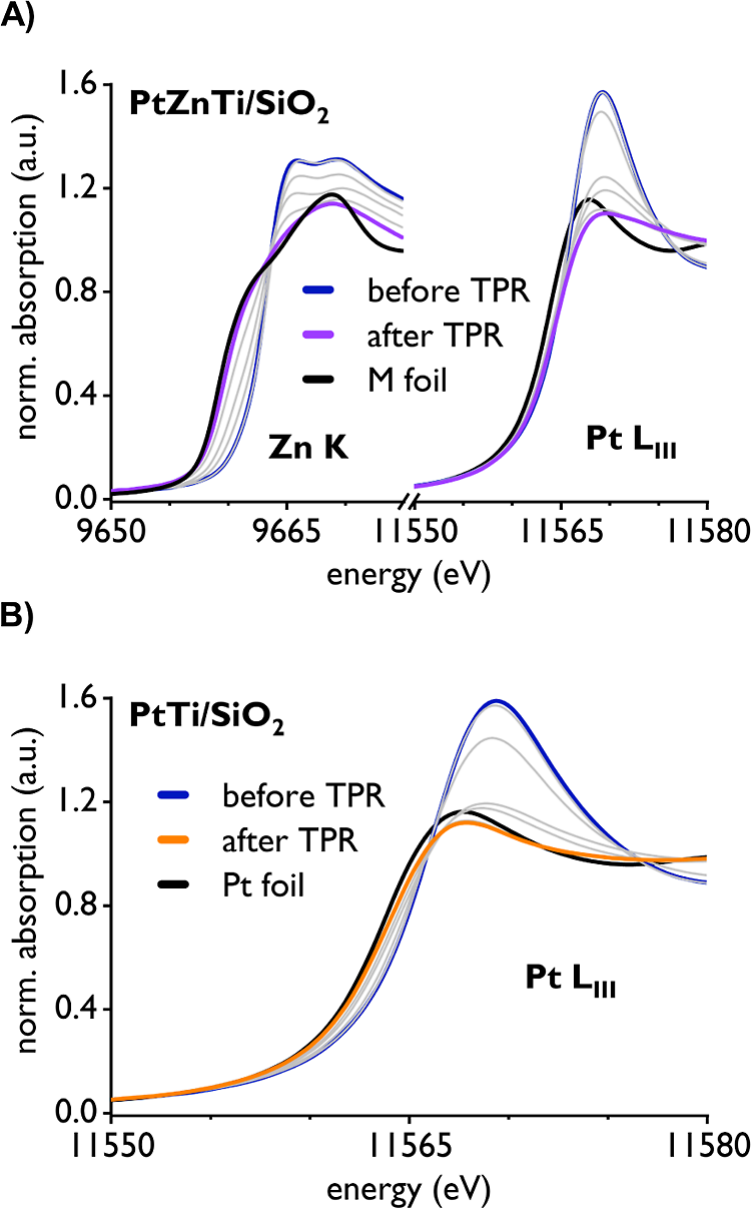
(A) Evolution of XANES spectra of Pt^II^_Zn^II^Ti^IV^/SiO_2_ (blue line) at the Zn K and Pt L_III_ edge under a flow of H_2_ from room temperature to 600 °C (purple line) and comparison to the respective metal foil (black line). (B) Evolution of XANES spectra of Pt^II^_Ti^IV^/SiO_2_ (blue line) at the Pt L_III_ edge under a flow of H_2_ from room temperature to 600 °C (orange line) and comparison to Pt foil (black line).

To gather more detailed information on the structure and composition of the NPs in PtTi/SiO_2_ and PtZnTi/SiO_2_ as well as the chemical state of Zn and Pt in the respective materials, we then performed an XAS study and compared the results to Pt/SiO_2_ and PtZn/SiO_2_ when adequate (see the Supporting Information for additional XAS spectra and information regarding *in situ* experiments). First, we carried out an *in situ* X-ray absorption near edge structure (XANES) analysis of Pt^II^_Ti^IV^/SiO_2_ (Pt L_III_-edge) and Pt^II^_Zn^II^Ti^IV^/SiO_2_ (both Pt L_III_-edge and Zn K edge) under a flow of H_2_ from room temperature to 600 °C to track the evolution of the metal oxidation state during particle formation (see the Supporting Information for details on the procedure). The results are depicted in Figure 3A,B. As seen from the Zn K edge spectra of Pt^II^_Zn^II^Ti^IV^/SiO_2_ during reduction, Zn is almost fully reduced to its metallic state at 600 °C under H_2_ which is manifested in an edge energy shift by −3.6 eV from 9663.3 to 9659.7 eV and changes in the white line (Figure 3A). This is also confirmed by a linear combination fit (LCF) of PtZnTi/SiO_2_ using the initial state (Pt^II^_Zn^II^Ti^IV^/SiO_2_) and Zn foil as references, revealing *ca.* 90% reduction of Zn (Figure S23). All these observations are very similar to what is observed for the same analysis at the Zn K edge for a similar PtZn/SiO_2_ material with a −3.8 eV edge energy shift upon reduction and *ca.* 80% reduced Zn according to LCF analysis.^10^ The Pt L_III_ edge for the same material (PtZnTi/SiO_2_) shows a similar trend to full reduction of Pt. The edge energy shifts by −1.6 eV from 11,566.2 to 11,564.6 eV, and a significant reduction in white line intensity can be observed which clearly indicates reduction of Pt. A very similar shift of −1.3 eV can be observed when performing the same experiment at the Pt L_III_ edge of PtZn/SiO_2_.

Comparison of additional *ex situ* spectra of Pt/SiO_2_ (11,564.0 eV, Figure S25) and PtZn/SiO_2_ (11,564.6 eV, Figure S26) reveals that PtZnTi/SiO_2_ behaves very similar to PtZn/SiO_2_ where the small differences in edge energy and white line compared to Pt foil (11,564.0 eV, Figure S26) can be attributed to a particle size effect and alloy formation, as described in earlier literature,^12,49,50^ while no significant effect on the Pt L_III_ and Zn K XANES signatures by Ti addition could be detected for these materials. Interestingly, the same analysis of Pt^II^_Ti^IV^/SiO_2_ (Figure 3B) at the Pt L_III_ edge reveals a much more similar XANES signature—edge energy and white line structure—between Pt foil (and also Pt/SiO_2_, albeit with small differences which could be attributed to both, Ti addition or a particle size effect; see Figure S25) and Pt^II^_Ti^IV^/SiO_2_ after reduction under an atmosphere of H_2_ at 600 °C (11,564.0 eV) than what is observed for Pt^II^_Zn^II^Ti^IV^/SiO_2_ after reduction. The data further support what was found in the CO adsorption FTIR study, *i.e.*, that Pt and Ti do not form an alloy in PtTi/SiO_2_, but rather that the material consists of Pt NPs supported on a Ti-decorated support (see Figure 1A). It also indicates that the differences in XANES signature at the Pt L_III_ edge for Pt^II^_Zn^II^Ti^IV^/SiO_2_ after reduction and Pt foil are mostly a result of alloy formation between Pt and Zn (with no Ti being alloyed) and less due to a particle size effect (as PtTi/SiO_2_ and PtZnTi/SiO_2_ have very similar PSD). However, under the assumption of full Pt reduction and *ca.* 90% Zn reduction and using the Elemental Analysis (EA) results [1.53 wt % Zn, 2.95 wt % Pt; 1:1.55 (Pt/Zn/mol/mol)], a ratio of 1:1.4 (Pt/Zn) can be calculated for the PtZn particles in PtZnTi/SiO_2_ which is similar to what is observed for PtZn/SiO_2_ [1:1.2 (Pt/Zn) in the particles].^10^

With these data in hand, we then performed an EXAFS analysis to obtain a more detailed understanding of the structure of the PtZn alloy in PtZnTi/SiO_2_ and potential interaction of Ti sites with the NPs. Table 4 summarizes the fitting data of PtTi/SiO_2_, PtZn/SiO_2_, and PtZnTi/SiO_2_ at the Pt L_III_ edge (for more detail regarding fitting, see the Supporting Information and Figures S34–S36). Interestingly, for all three materials, two different Pt-M pathways needed to be included to obtain reasonable fits. In particular, for the PtTi/SiO_2_, the requirement to include two Pt–Ti scattering paths in order to obtain a reasonable fit was indicated (see Figure S34 and Tables 4 and S3). This requirement is consistent with what has been already shown by the chemisorption studies, namely, the presence of Ti strongly alters the properties of the Pt NPs, which is likely due to the strong Pt–Ti interaction, while no indication of alloying is detected according to XANES and CO adsorption FTIR. The requirement for two different paths can likely be attributed to interacting Ti species in different structural environments. In the case of PtZn/SiO_2_ and PtZnTi/SiO_2_, Pt–Zn as well as Pt–Pt scattering paths are needed with very similar coordination numbers, indicating that both materials feature alloyed PtZn particles of similar structure which was already indicated by preceding analyses. The requirement of two different Pt–Zn scattering pathways can either be explained by the formation of a hexagonal close-packed structure in both cases which features different M–M′ distances or by high heterogeneity of rather amorphous NPs which is likely due to the very small particle sizes. In the case of PtZn/SiO_2_, the data (coordination numbers) are in line with an earlier report while extending it by a more detailed analysis, revealing the need for two different Pt–Zn distances.^10^ In the case of PtZnTi/SiO_2_, the simultaneous inclusion of a Pt–Zn and Pt–Ti scattering pathway was not possible due to a too high complexity of the fit, while it is highly likely that Pt–Ti interaction is also present in the case of the trimetallic material. The data at the Pt L_III_ edge are complemented by additional Zn K edge data which support the need for two Pt–Zn distances in both cases (for more details, see the Supporting Information and Figures S37 and S38).

**Table 4:** EXAFS Fit Parameters: PtTi/SiO_2_, PtZn/SiO_2_, and PtZnTi/SiO_2_ (Pt L_III_ Edge)^a^

material	path	nearest neighbors	*r* [Å]	σ^2^ [Å]	Δ*E*_0_	*R* factor
PtTi/SiO_2_	Pt–Ti_1_	0.9 ± 0.4	2.46 ± 0.04	0.0066 ± 0.0024	3.7 ± 2.1	0.015
	Pt–Ti_2_	1.4 ± 0.7	2.69 ± 0.05			
	Pt–Pt	6.6 ± 1.1	2.67 ± 0.02			
PtZn/SiO_2_	Pt–Zn_1_	2.4 ± 0.8	2.50 ± 0.09	0.0073 ± 0.0062	2.7 ± 5.5	0.021
	Pt–Zn_2_	2.1 ± 0.7	2.63 ± 0.13			
	Pt–Pt	2.9 ± 0.9	2.67 ± 0.06			
PtZnTi/SiO_2_	Pt–Zn_1_	2.1 ± 0.6	2.49 ± 0.07	0.0060 ± 0.0053	2.3 ± 4.8	0.021
	Pt–Zn_2_	1.6 ± 0.5	2.63 ± 0.11			
	Pt–Pt	3.0 ± 1.2	2.66 ± 0.05			

a Notation: *r*, scattering path length between absorber and scatterer. σ^2^, mean square relative displacement (Debye-Waller-Factor). Δ*E*_0_, internal energy alignment.

Due to the diverging behavior of PtZn/SiO_2_ and PtZnTi/SiO_2_ under short and long deactivation/regeneration phases (*vide supra*), we were also interested in the structural evolution and potential oxidation state changes of Zn and Pt in the two materials under regeneration conditions. We therefore performed an *in situ* XAS study at the Zn K and Pt L_III_ edge (see the Supporting Information for more detail). The results indicate that under oxidizing conditions (O_2_/Ar) at high temperatures, Zn is partially (PtZnTi/SiO_2_) or almost fully (PtZn/SiO_2_) oxidized to Zn^II^ species. The Pt L_III_ edge structure of both materials resembles very closely the one of Pt foil after such treatment, indicating dealloying in both materials while Pt is not oxidized significantly even at high temperatures (see Figures S28 & S31). Under the subsequently applied reducing conditions (H_2_), Zn^II^ species are re-reduced to Zn^0^ in both cases (see Figures S29 & S30 and S32 & S33). In the case of PtZn/SiO_2_, the data suggest that a different type of Zn^0^ is formed compared to pristine PtZn/SiO_2_. In contrast, for PtZnTi/SiO_2_, the reformed Zn^0^ species resemble quite closely the ones observed in pristine PtZnTi/SiO_2_. Changes at the Pt L_III_ edge align well with the Zn K edge results in that in PtZn/SiO_2_ the edge structure resembles a state in between Pt foil and pristine PtZn/SiO_2_. This indicates partial re-alloying, while the edge structure in PtZnTi/SiO_2_ after reduction resembles closely the one of pristine PtZnTi/SiO_2_. Note that coke removal is likely not fully efficient during the regeneration cycles and thus small differences in Pt/Zn interaction with carbon species could also contribute to the observed spectral changes.

#### 2.3.3 Electron Paramagnetic Resonance Analysis

**Figure 4:**
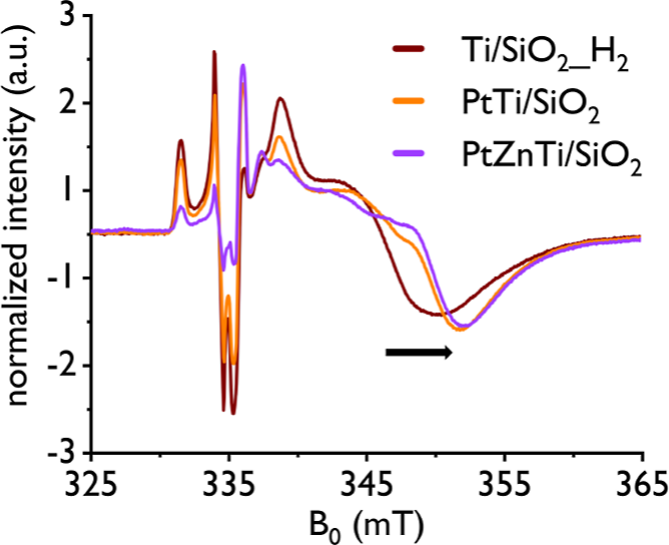
X-band cw spectra of Ti/SiO_2__H_2_ (brown), PtTi/SiO_2_ (orange), and PtZnTi/SiO_2_ (purple), recorded at 20 K. For the spectral features attributed to Ti^III^, a slight shift to higher *B*_0_ field (indicated with a black arrow) can be observed while going from the monometallic Ti to the bi- and trimetallic PtTi and PtZnTi materials.

In order to better understand the nature of Ti sites and their interaction with the NPs, and to explain the observed catalytic behavior, we next conducted an EPR study. First, in the X-band continuous wave (cw) EPR spectra, an identical superoxide signal could be detected in the low field region of the spectrum for all three materials Ti/SiO_2__H_2_, PtTi/SiO_2_, and PtZnTi/SiO_2_ (see Figure 4). The superoxide species features a rhombic *g* tensor (see Table S8), which is in line with the literature.^51,52^ Such superoxide species likely results from the interaction of trace amounts of (adventitious) O_2_ in the glovebox atmosphere with Ti^III^ sites. In addition, the multi-frequency EPR spectra of these materials reveal that at least two Ti^III^ species are present in all the materials (see Figure S39 and Table S8). Importantly, the cw EPR spectroscopic signature of these Ti^III^ species varies between the different materials, shifting the high field minimum to slightly lower *g* values when going from Ti/SiO_2__H_2_ to PtTi/SiO_2_ and PtZnTi/SiO_2_ (see Figure 4). This effect is not necessarily only due to a genuine increase of the lowest principal *g* value but could also be due to increased line broadening, observed for the Ti^III^ species in PtTi/SiO_2_ and PtZnTi/SiO_2_ materials, which is best explained by the presence of Ti^III^–Pt interactions. The pronounced spectral change when comparing Ti/SiO_2__H_2_ with PtTi/SiO_2_ and PtZnTi/SiO_2_, but minor changes between PtTi/SiO_2_ and PtZnTi/SiO_2_ (see Figure 4), indicates that the Ti^III^–Pt interaction is the determining factor for these spectral changes, while Zn contribution is minor. However, no direct Ti^III^–Pt interactions can be detected in the corresponding hyperfine sublevel correlation spectroscopy (HYSCORE)^53^ spectra (see Figures S40 and S41).

In addition, quantitative EPR measurements reveal that only about 1.5–2% of all Ti species are Ti^III^ EPR-active sites in the investigated materials (Figure S42). The obtained Ti^III^ amount would correspond to roughly 0.5 Ti^III^ sites per NP in the PtTi and PtZnTi samples. Such a low amount of Ti^III^ species in the Pt containing materials seems unlikely as noble metals like Pt usually help with the reduction of cationic species with low reducibility.^30^ In that context, it is also surprising that the amount of reduced Ti species appears almost identical in all three cases. While for the Ti/SiO_2__H_2_ material, the low amount of Ti^III^ agrees with previous reports of Ti^IV^ reduction on SiO_2_ being very challenging,^43^ a higher amount of reduced Ti^III^ should be expected for the Pt containing materials. A possible explanation is that the Ti^III^ species with strong Ti^III^–Pt interaction are not detected by EPR. For an even number of Ti^III^ sites interacting with a Pt NP, this could be due to spin recombination and the formation of a *S* = 0 spin system. In the case of odd numbers of Ti^III^ sites interacting with a Pt NP, the resulting *S* = 1/2 spin systems could be undetectable due to fast spin relaxation and resulting signal broadening beyond the detection limit. The observable species therefore most likely correspond to isolated Ti^III^ sites with only a weak Ti^III^–Pt interaction, indicated by spectral shift and line broadening in the cw EPR spectra (Figure 4), agreeing well with the earlier mentioned absence of a direct Ti^III^–Pt coupling in the HYSCORE spectra (see Figures S40 and S41).

### 2.4 Computational Analysis of the Pt–Ti Interaction

The results from chemisorption, XAS analyses, EPR, and microscopy indicate a significant effect of Ti-doping on the properties of monometallic Pt as well as bimetallic PtZn particles supported on SiO_2_. Additionally, *in situ* XAS recorded during PDH and regeneration studies hints at a beneficial effect of Ti on the regeneration of PtZn NPs through sintering prevention. Taken together, all this information clearly points to a specific Pt–Ti interaction, while no indication of alloying has been found.

**Figure 5:**
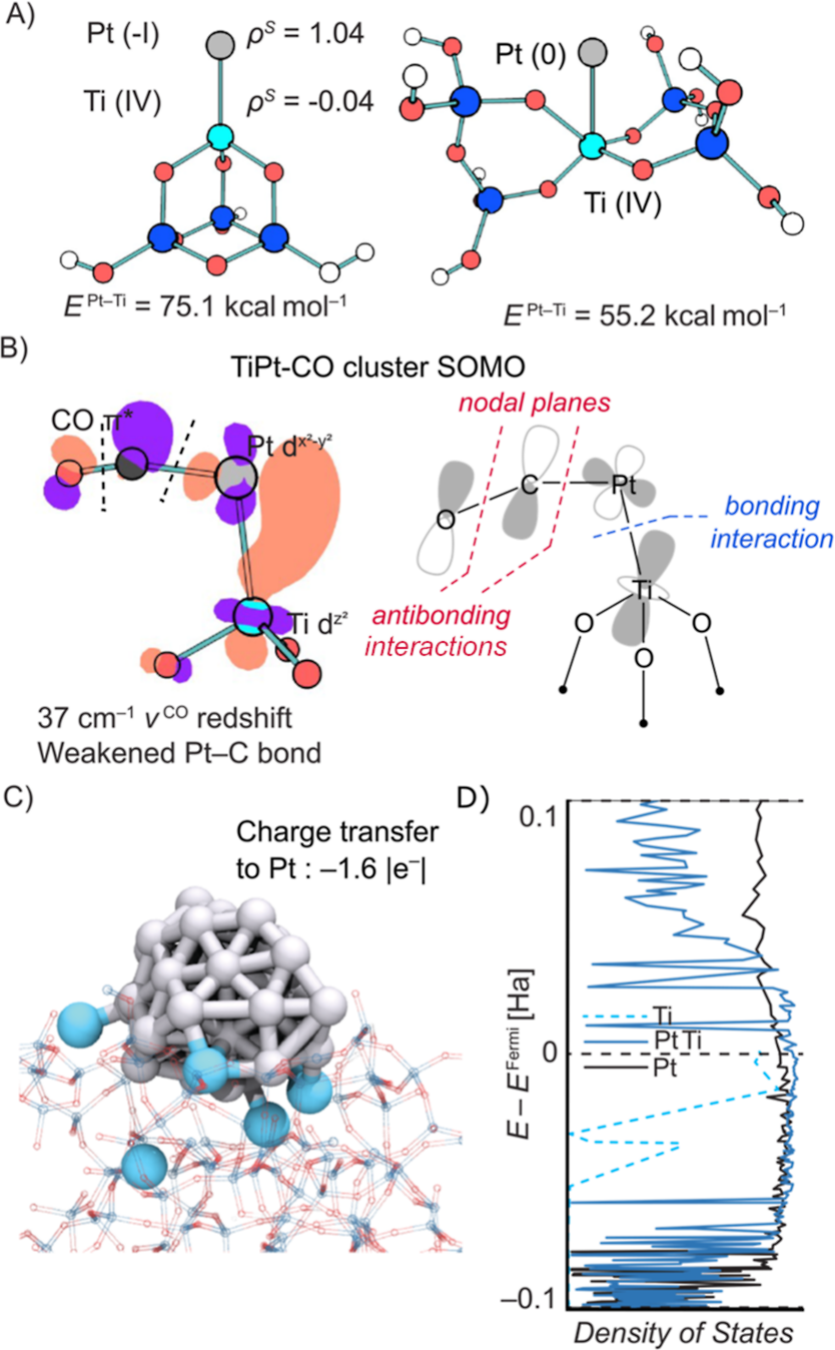
(A) Interaction of a single Pt atom with a Ti site in **Ti**^**III**^**Pt** and **Ti**^**IV**^**Pt** model clusters. In the case of Ti^III^, the entire spin density is transferred to Pt, yielding a Pt–^I^–Ti^IV^ site. (B) Isosurface and simplified drawing of the SOMO of **Ti**^**III**^**Pt(CO)**, evidencing electron transfer from Ti and Pt to the Pt–C–O antibonding three-center orbital. (C) “Full-scale” **Pt**_**38**_**Ti/SiO**_**2**_ model highlighting interaction between the Ti sites and the Pt NPs. (D) PDOS for **Pt**_**38**_**/SiO**_**2**_, **Ti/SiO**_**2**_ (support with five representative Ti sites), and **Pt**_**38**_**Ti/SiO**_**2**_ models (see the computational section of the Supporting Information for details). The increase in the d-band density below the Fermi-level between **Pt**_**38**_**/SiO**_**2**_ and **Pt**_**38**_**Ti/SiO**_**2**_ agrees with electron density transfer from Ti to Pt.

We set out to understand the nature of the interaction of surface Ti with the Pt and PtZn NPs, using computational modeling based on DFT. Simple cluster models (**Ti**^**III**^**Pt** and **Ti**^**IV**^**Pt**, see the computational section of the Supporting Information) were investigated at the PBE0^54^ level using Gaussian,^55^ in order to identify the nature of the Pt–Ti interaction. We first considered the interaction of a single Pt atom with Ti^III^ and Ti^IV^-surface sites (see Figure 5A). The coordination of a Pt atom to Ti^III^ sites in **Ti**^**III**^**Pt** is associated with a strong interaction energy (Δ*E* = −75.1 kcal mol^–1^), which is significantly larger than the one obtained for the coordination of a Pt atom to the corresponding Ti^IV^ site in **Ti**^**IV**^**Pt** (Δ*E* = −55.2 kcal mol^–1^). Similar trends were obtained using periodic amorphous models of Ti^III^ and Ti^IV^ sites on a ∼4 nm^2^ amorphous silica model optimized with the CP2K package^56^ (see the computational section of the Supporting Information and Figures S47–S50 for additional detail). To understand the high affinity of Ti^III^ for Pt^0^, the nature of the Pt^0^–Ti^III^ interaction was studied in more detail using an adequate simple cluster model. The strong interaction energy between a Pt atom and the parent Ti^III^ site is a result of electron donation from the d-orbitals of Pt into empty d orbitals of Ti and the back donation from the unpaired electron located in the d*z*^2^ of Ti^III^ into the empty 6s orbital of Pt. This electron transfer is reflected in an increase of the partial charge on Pt (−0.20, see MO diagram in Figure S43), as shown by a natural bond orbital (NBO) charge analysis. A similar transfer from Ti^III^ to M was experimentally described in the case of TiO_*x*_-supported Pd and Pt NPs.^57,58^ Notably, the spin density is fully transferred onto Pt which indicates that the originally unpaired d-electron of Ti^III^ is now fully localized on Pt (Figure 5A); it is thus best to describe the Pt–Ti pair as Pt^-I^–Ti^IV^ rather than Pt^0^–Ti^III^. Such a change in the electronic structure is expected to affect the adsorption properties of Pt as well as the spectroscopic signature of Ti (see Section 2.3.3).

We next evaluated how this electron transfer affects the interaction of CO with Pt. The interaction energy of CO in **Ti**^**III**^**Pt(CO)** is nearly 50 kcal mol^–1^ less favorable than the one calculated for **Ti**^**IV**^**Pt(CO)** (Figures S44 and S46 and Table S10). This trend is consistent with the experimentally observed decrease of CO-coverage for PtTi/SiO_2_ compared to Pt/SiO_2_. Furthermore, a red-shift of the CO-vibrational frequency (from 2007 to 1968 cm^–1^) is also predicted, in qualitative agreement with observation, albeit with a smaller experimental shift of 5 cm^–1^, likely due to the simplified model and low coverage (compare to Figure 1D and Table 1). Notably, this red shift is shown to originate from the population of the antibonding SOMO, resulting from interaction of the antibonding CO−π* with the Pt–Ti bonding orbital (Figure 5B, full MO diagram in Figure S44). The resulting MO has a C–O antibonding character; population of this orbital thus leads to a decreased CO bond strength and red-shifted CO in IR. At the same time, the SOMO is antibonding relatively to the C–Pt bond so that the CO coordination energy decreases sharply. The agreement between experimental and calculated properties of adsorbed CO further supports the description of the Pt–Ti pair as Pt^-I^–Ti^IV^ sites.

We next considered a larger (more realistic) system consisting of a dehydroxylated silica model featuring ∼1 Ti/nm^2^ in five representative Ti sites (**Ti/SiO**_**2**_, 4x Ti^III^ and 1x Ti^IV^, Figure S51) on which a Pt_38_ NP (ø *ca.* 1 nm) is supported (**Pt**_**38**_**Ti/SiO**_**2**_, Figure 5C, see the computational section of the Supporting Information for detail). This model was investigated at the rev-PBE/MOLOPT-DZ level^59−62^ using the CP2K package. The interaction energy of the Pt_38_ NP with the surface is more than doubled in the presence of Ti sites compared to the case of a simple SiO_2-700_ surface [−318 kcal mol^–1^ (**Pt**_**38**_**Ti/SiO**_**2**_) *vs* −156 kcal mol^–1^ (**Pt**_**38**_**/SiO**_**2**_)].^42^ This further supports that isolated Ti sites are able to stabilize Pt NPs. Similar to the simple cluster model, spin density transfer from Ti^III^ sites onto the Pt_38_ particle is also observed in **Pt**_**38**_**Ti/SiO**_**2**_. The spin density of 3.9 unpaired electrons in the Ti-doped model support is transferred to Pt upon adding the Pt_38_ NP to the system, where spin recombination results in a low-spin configuration (*S* = 0) and an EPR-silent (Pt^δ−^-Ti^IV^) system (Table S12). *S* = 1/2 systems are also conceivable in case of odd numbers of Ti^III^ sites interacting with Pt NPs as mentioned already. Such a system could not be detected experimentally (see Section 2.3.3).

Additionally, Ti to Pt charge transfer is evidenced by the variation of the density-derived atomic point (DDAP) charge (which is implemented in CP2K and was developed for periodic calculations^63^) on the Pt_38_ NP between the Ti-functionalized SiO_2-700_ and the non-functionalized SiO_2-700_ (1.6 |e^–^| transferred to the Pt_38_ NP, Table S11). This is supported by the variation of the projected density of states (PDOS) in this model, which shows electron transfer from Ti to Pt. The occupied d-band of Ti becomes unoccupied upon addition of the Pt_38_ NP, while the d-band of Pt gains density below the Fermi level when Ti sites are present at the surface (Figure 5D), similar to what was obtained for the simple model (*vide supra*).

Overall, the presence of Ti^III^ sites at the interface with Pt NPs induces an electron transfer from Ti to Pt, leading to a strong interaction, which is likely responsible for the stabilization of small Pt NPs during PDH and modifies the adsorption properties of Pt NPs.

### 2.5 Conclusions

In this work, we have described the synthesis, the detailed spectroscopic characterization, and the PDH performances of Pt and PtZn NPs supported on Ti-doped silica and compared them to their silica-supported analogues. We show in particular that the presence of Ti sites at the surface of silica prevents sintering of Pt and PtZn NPs during PDH and modifies the CO/H_2_ adsorption properties as well as the CO IR signatures. A combined chemisorption, EPR, XAS, electron microscopy, and computational study demonstrate that there is a strong interaction between Pt NPs with Ti surface sites, in the absence of bulk TiO_*x*_. While Pt^0^ interacts only weakly with Ti^IV^ sites, such an interaction is highly favored with Ti^III^ sites. In fact, the interaction of Ti^III^ sites with Pt^0^ leads to a very strong Pt–Ti bond, accompanied by an electron transfer from Ti^III^ to Pt^0^, formally generating Pt^-I^–Ti^IV^ systems. Similar results are obtained for a more realistic model which considers the interaction of SiO_2_ supported Ti sites with a Pt NP, showing a strong interaction energy of the Pt NP with the Ti doped compared to the pure SiO_2_ support. The interaction is accompanied by Ti to Pt spin density transfer and recombination on the NPs. The strong interaction of Ti sites with Pt NPs also results in a change of chemisorption properties and the observation of slightly red-shifted CO IR bands by comparison with the silica-supported systems. Notably, a sharp decrease of adsorption of H_2_ and CO—as often reported for classical SMSI in Pt/TiO_2_ and related systems—is observed. While this change in adsorption properties is often associated with a coverage of the Pt NPs with TiO_*x*_ species in the case of Pt/TiO_2_, we could demonstrate that electronic density transferred from a formally reduced Ti site to Pt can also induce a change of CO adsorption properties. The interaction of CO with Pt results in the population of an orbital with C–O and C–Pt antibonding character, lowering the CO adsorption strength, yet causing a red-shifted CO vibrational feature.

### 2.6 Experimental Section

#### 2.6.1 General Considerations

All operations were performed in a M. Braun glovebox under an argon atmosphere or using standard Schlenk techniques. Solvents were purified using a solvent purification system and stored over molecular sieves. SiO_2-700_ was prepared by heating Aerosil (200 m^2^/g) to 500 °C (ramp of 300 °C/h) in air and then calcining in air for 12 h. Afterward, the material was evacuated at high vacuum (10^–5^ mbar) maintaining 500 °C for 8 h, followed by heating to 700 °C (ramp of 60 °C/h), and maintaining 700 °C for 24 h. The molecular complexes [Ti(OSi(OtBu)_3_)_3_(O^i^Pr)], [Zn(OSi(OtBu)_3_)_2_]_2_, and [Pt(OSi(OtBu)_3_)_2_(COD)] were prepared according to the literature procedures.^47,64,65^ Supported species **Ti**^**IV**^**/SiO**_**2**_ (elemental analysis: Ti, 0.6 wt %), supported platinum NP **Pt**^**0**^**/SiO**_**2**_ (elemental analysis: Pt, 3.96 wt %), and the supported species **PtZn/SiO**_**2**_ (elemental analysis: Pt, 3.13 wt %; Zn, 1.63 wt %) were prepared according to the literature procedures.^10,44,46^

Transmission IR spectra were recorded using a Bruker Alpha FTIR spectrometer at 2 cm^–1^ resolution. For CO adsorption followed by FTIR, pellets of *ca.* 10 mg and pressures of 10–120 mbar were used. HAADF-STEM images were recorded on a FEI Talos F200X instrument operated at 200 keV. For data analysis, the standard software ImageJ (version 1.52a) was used. Chemisorption experiments were performed using a BEL JAPAN BELSORP-MAX instrument. Materials were loaded into cells in an Ar-filled and solvent-free glovebox. Pretreatment for H_2_ and CO chemisorption measurements involved heating the samples at 300 °C for 3 h under dynamic vacuum. XAS measurements were carried out at the Zn K-edge and Pt L_III_-edge at the SuperXAS beamline at SLS (PSI, Villigen, Switzerland). Data processing was carried out by standard procedures using ProXASGui software developed at the SuperXAS beamline, PSI, Villigen. The program package Demeter was used for data analysis.^66^ The *S*0^2^ value for the Pt L_III_-edge (0.82 ± 0.02) and for the Zn K-edge (0.90 ± 0.09) was obtained by fitting of Pt and Zn foil, respectively.^22^ X-band *ex situ* cw EPR spectra of the materials were recorded on an Elexsys E580 EPR spectrometer (Bruker Biospin, Rheinstetten Germany), equipped with an ESR900 helium flow cryostat (Oxford Instruments, Oxfordshire, UK) and a Super High Q (SHQ) resonator (Bruker Biospin), at 20 K. X-band *ex situ* echo-detected field sweeps (EDFS) and HYSCORE measurements were performed at 10 K on a Bruker Elexsys E680 EPR spectrometer, equipped with a helium flow cryostat (Oxford Instruments, Oxfordshire), using a MS3 split-ring resonator (Bruker Biospin). Q-band *ex situ* EDFS were recorded on a homebuilt Q-band spectrometer^67^ equipped with a helium flow cryostat (Oxford Instruments, Oxfordshire), using a homebuilt 3 mm resonator.^68^ DFT calculations for the simple cluster models were performed using the Gaussian 09 (Rev D.01) suite of programs.^55^ Structures of minima were optimized using the B3LYP functional.^69−72^ First to third period atoms (H, C, O, and Si) were described using the Pople basis set 6-31+G(d). The Stuttgart/Cologne group effective-core potential and its associated triple-zeta basis set were used to describe Ti and Pt.^73^ Basis set superposition error was corrected using the counterpoise method, as implemented in Gaussian 09 (Rev D.01). Charge, orbital, and spin-population analyses were performed *via* NBO analysis.^74,75^ Structures were visualized with VMD and Chemcraft. Calculations for the more realistic models of Ti-doped silica were carried out using CP2K 3.0.^56^ Structures were optimized using the revised version of the Perdew–Burke–Ernzerhof GGA functional^59,60^ in conjunction with double-ζ MOLOPT basis set^61,62^ and Goedecker-Teter-Hutter pseudopotentials^76^ on all atoms. The D3 empirical dispersion correction^77^ was employed. The Pt_38_ NP was randomly generated using the Packmol package.^78^ Calculations for equilibration at 873 K during at least 1 ps with molecular dynamics at the DFT level (AIMD), and Mulliken,^79^ Hirshfeld,^80^ Löwdin^81^ populations, as well as DDAP^63^ charges, and the PDOS determination were all performed using the CP2K 7.1 package.^56^

#### 2.6.2 Synthesis of Zn^II^Ti^IV^/SiO_2_

To **Ti**^**IV**^**/SiO**_**2**_ (1.025 g) in benzene in a Schlenk flask was added **[Zn(OSi(OtBu)**_**3**_**)**_**2**_**]**_**2**_ (0.183 g, 0.154 mmol) in benzene while stirring (100 rpm). The mixture was stirred at RT for 12 h. The supernatant was decanted, and the material was washed with benzene. The material was dried *in vacuo* to receive **Zn**^**II**^**_Ti**^**IV**^**/SiO**_**2**_ as a white solid. The white material was then transferred to a tubular quartz reactor which was set under high vacuum (10^–5^ mbar) and successively heated to 300 °C (ramp of 5 °C/min) for 1 h, 400 °C (ramp of 5 °C/min) for 1 h, 500 °C (ramp of 5 °C/min) for 1 h, 600 °C (ramp of 5 °C/min) for 12 h yielding **Zn**^**II**^**Ti**^**IV**^**/SiO**_**2**_ as a white solid.

#### 2.6.3 Synthesis of PtTi/SiO_2_

To **Ti**^**IV**^**/SiO**_**2**_ (0.434 g) in benzene in a vial was added **[Pt(OSi(OtBu)**_**3**_**)**_**2**_**(COD)]** (0.108 g, 0.130 mmol) in benzene while stirring (1000 rpm). The mixture was stirred at RT for 10 h. The supernatant was decanted, and the material was washed with benzene. The material was dried *in vacuo* to receive **Pt**^**II**^**_Ti**^**IV**^**/SiO**_**2**_ as a white solid. The material was treated in a tubular quartz flow-reactor slowly heated to 600 °C (ramp of 5 °C/min) under a steady flow of H_2_ and then treated at this final temperature for 9 h, yielding **PtTi/SiO**_**2**_ as a black material.

#### 2.6.4 Synthesis of PtZnTi/SiO_2_

To **Zn**^**II**^**Ti**^**IV**^**/SiO**_**2**_ (0.842 g) in benzene in a vial was added **[Pt(OSi(OtBu)**_**3**_**)**_**2**_**(COD)]** (0.209 g, 0.252 mmol) in benzene while stirring (1000 rpm). The mixture was stirred at RT for 12 h. The supernatant was decanted, and the material was washed with benzene. The material was dried *in vacuo* to receive **Pt**^**II**^**_Zn**^**II**^**Ti**^**IV**^**/SiO**_**2**_ as a white solid. The material was treated in a tubular quartz flow-reactor slowly heated to 600 °C (ramp of 5 °C/min) under a steady flow of H_2_ and then treated at this final temperature for 12 h, yielding **PtZnTi/SiO**_**2**_ as a black material.

#### 2.6.5 Catalytic Tests

Catalytic tests were performed utilizing a quartz flow-reactor designed and a heating/flow setup designed and manufactured by Micromeritics Instrument Cooperation (PID Eng & Tech). Catalyst samples were loaded into a quartz tubular reactor in an Ar-filled glovebox. Reaction temperatures were maintained utilizing a quartz encased thermocouple maintained in contact with the catalyst dispersed in SiC. The output gas composition was analyzed automatically by a GC with a flame ionization detector which was programmed to sample the gas stream every 9 min throughout the reaction. Gas composition and flow rate with a flow of 50 mL/min with 1:4 C_3_H_8_/Ar ratio (v/v) were maintained at all times. In all cases, conversions below equilibrium were achieved.

Detailed experimental procedures, instrument specifications, and characterization data are covered in greater detail in the Supporting Information.

## Supporting Information Available

Detailed experimental procedures, general considerations, microscopy data, computational details, spectroscopy data of all methods, and associated data (PDF)
