# Supplementary material for: Ti-Doping in Silica-Supported PtZn Propane Dehydrogenation Catalysts: From Improved Stability to the Nature of the Pt–Ti Interaction
Source: JACS Au. 2023 Jun 30;3(7):1939–51. doi: 10.1021/jacsau.3c00197 (PMC10369412; doi:10.1021/jacsau.3c00197)
Supplement: Supplementary file 1 — au3c00197_si_001.pdf [file au3c00197_si_001.pdf]

Ti-Doping in a Silica-Supported Pt-Zn Propane Dehydrogenation Catalyst: From Improved Stability to the Nature of the Pt-Ti Interaction

Lukas Rochlitz,[a] Jörg W. A. Fischer,[a] Quentin Pessemesse,[b] Adam H. Clark,[c] Anton Ashuiev,[a] Daniel Klose,[a] Pierre-Adrien Payard,[b] Gunnar Jeschke,[a] Christophe Copéret[a,*]

[a] Department of Chemistry and Applied Biosciences, ETH Zürich, Vladimir-Prelog-Weg 2, CH-8093 Zürich, Switzerland.

[b] Univ Lyon, Université Claude Bernard Lyon I, CNRS, INSA, CPE, UMR 5246, ICBMS, rue Victor Grignard, F-69622 Villeurbanne Cedex, France.

[c] Paul Scherrer Institut, CH-5232 Villigen, Switzerland.

Experimental 2

Synthesis of Ti^{IV}/SiO_2 2

Synthesis of $Zn^{II}Ti^{IV}/SiO_2$ 3

Synthesis of $PtZnTi/SiO_2$ 4

Synthesis of $PtTi/SiO_2$ 5

STEM/EDX 6

$PtTi/SiO_2$ 7

$PtZn/SiO_2$ 7

$PtZnTi/SiO_2$ 9

FTIR CO Adsorption 12

H_2 and CO Chemisorption 13

XAS 16

Ex situ Zn K edge XANES 17

Ex situ Pt L_{III} edge XANES 18

In situ Regeneration Study at the Zn K and Pt L_{III} Edge 20

EXAFS Analysis at the Pt L_{III} Edge 27

EXAFS Analysis at the Zn K Edge 30

Electron Paramagnetic Resonance Spectroscopy 32

Computational Section 32

Catalytic Propane Dehydrogenation 45

References 55

Experimental

All operations were performed in a M. Braun glove box under an argon atmosphere or using high vacuum and standard Schlenk techniques. Pentane was purged with argon for 30 minutes and dried using a MB SPS 800 solvent purification system where columns used for pentane purification were packed with activated copper and alumina. Benzene was either distilled from purple Na^0/benzophenone or obtained from the MB SPS system and used without further purification. Deuterated benzene (C_6D_6) was distilled from purple Na^0/benzophenone. All solvents were stored over 4 Å molecular sieves after being transferred to a glove box. 4 Å molecular sieves were activated under high vacuum overnight at 300°C. SiO_{2-700} was prepared by heating Aerosil® (200 m^2/g) to 500°C (ramp of 300°C/h) in air and then calcining in air for 12 hours. Afterwards, the material was evacuated at high vacuum (10^{-5} mbar) keeping 500°C for 8 hours, followed by heating to 700°C (ramp of 60°C/h), and maintaining 700°C for 24 hours. Titration of the SiO_{2-700} using **[$Mg(CH_2Ph)_2(THF)_2$]** purified *via* sublimation prior to use, yielded an Si-OH density of 0.3 mmol/g, corresponding to 0.9 accessible Si-OH groups per nm^2. The molecular complexes **[$Ti(OSi(OtBu)_3)_3(O^iPr)$]**, **[$Zn(OSi(OtBu)_3)_2$]$_2$** and **[$Pt(OSi(OtBu)_3)_2(COD)$]** were prepared according to literature procedures.[1–3] Propane was purchased from Carbagas and of 99.95% purity. All other reagents were purchased from Sigma-Aldrich or Acros Organics and used as received. The supported species **Ti^{IV}/SiO_2** (Elemental analysis: Ti, 0.6 wt%), supported platinum nanoparticles **Pt^0/SiO_2** (Elemental analysis: Pt, 3.96 wt%) and the supported species **$PtZn/SiO_2$** (Elemental analysis: Pt, 3.13 wt%; Zn, 1.63 wt%) were prepared according to literature procedures.[4–6] Transmission IR spectra were recorded using a Bruker Alpha FT-IR spectrometer. Mikroanalytisches Labor Pascher (An der Pulvermühle 1, 53424 Remagen-Bandorf, Germany) performed elemental analyses (ICP-AES for metals).

Synthesis of Ti^{IV}/SiO_2

SiO_{2-700} (2.234 g, 0.670 mmol -OH) was added to a 100 ml Schlenk flask. Benzene (about 10 ml) was added slowly while stirring to give a white suspension. **[$Ti(OSi(OtBu)_3)_3(O^iPr)$]** (0.602 g, 0.671 mmol) was added slowly to the suspension as a white solution in benzene (about 10 ml) while stirring (100 rpm). The resulting suspension was stirred at RT for 12 h. The benzene on top of the silica material was decanted and the material washed with benzene (10 ml) two times to wash off unreacted

complex. The material was then washed with pentane before it was dried *in vacuo* to receive $Ti^{IV}\_SiO_2$ as a white solid. The white material was then transferred to a tubular quartz reactor. The reactor was set under high vacuum (10^{-5} mbar

) and successively heated to 300°C (ramp of 5°C/min) for 1 h, 400°C (ramp of 5°C/min) for 1 h, 500°C (ramp of 5°C/min) for 1 h, 600°C (ramp of 5°C/min) for 12 h yielding **Ti^{IV}/SiO_2** as white solid. Elemental Analysis: Ti, 0.59 wt%. **Remark**: To obtain **$Ti/SiO_2\_H_2$**, freshly prepared **Ti^{IV}/SiO_2** was treated under H_2 at 600 °C (ramp of 5 °C/min) for an additional 10 h.

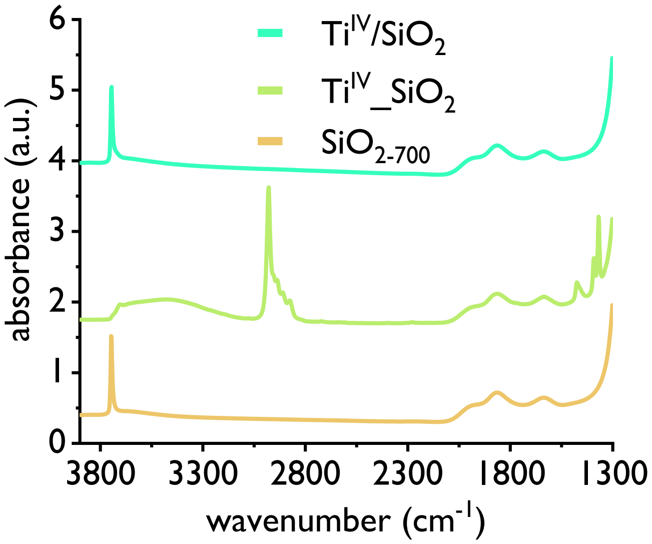

Figure S1. Transmission FTIR spectra of **$SiO_{2\text{-}700}$** (light orange), **$Ti^{IV}\_SiO_2$** (green) and **Ti^{IV}/SiO_2** (turquoise) normalized to the $\upsilon_{Si\text{-}O}$ vibrational frequency at 1865 cm^{-1}.

Synthesis of $Zn^{II}Ti^{IV}/SiO_2$

Ti^{IV}/SiO_2 (1.025 g) was added to a 50 ml Schlenk flask. Benzene (about 5 ml) was added slowly while stirring to give a white suspension. **$[Zn(OSi(OtBu)_3)_2]_2$** (0.183 g, 0.154 mmol (note that it is a dimer)) was added dropwise to the suspension as a colorless solution in benzene (about 10 ml) while stirring (100 rpm). The resulting white suspension was stirred at RT for 12 h. The benzene on top of the silica material was decanted and the material washed with benzene (10 ml) two times to wash off unreacted complex. The material was then washed with pentane before it was dried *in vacuo* to receive **$Zn^{II}\_Ti^{IV}/SiO_2$** as a white solid. The white material was then transferred to a tubular quartz reactor. The reactor was set under high vacuum (10^{-5} mbar) and successively heated to 300°C (ramp of 5°C/min) for 1 h, 400°C (ramp of 5°C/min) for 1 h,

500°C (ramp of 5°C/min) for 1 h, 600°C (ramp of 5°C/min) for 12 h yielding **$Zn^{II}Ti^{IV}/SiO_2$** as white solid.

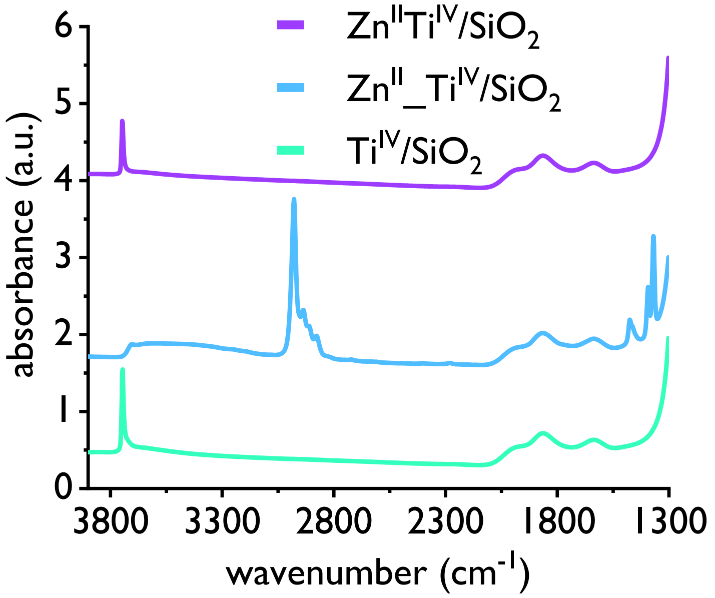

Figure S2. Transmission FTIR spectra of **Ti^{IV}/SiO_2** (turquoise), **$Zn^{II}\_Ti^{IV}/SiO_2$** (blue) and **$Zn^{II}Ti^{IV}/SiO_2$** (purple) normalized to the $\upsilon_{Si\text{-}O}$ vibrational frequency at 1865 cm^{-1}.

Synthesis of PtZnTi/SiO_2

$Zn^{II}Ti^{IV}/SiO_2$ (0.842 g) was added to a 20 ml screw cap vial. Benzene (about 5 ml) was added slowly while stirring to give a white suspension. **[Pt(OSi(OtBu)$_3$)$_2$(COD)]** (0.209 g, 0.252 mmol) was added dropwise to the suspension as a colorless solution in benzene (about 10 ml) while stirring (1000 rpm). The resulting white suspension was stirred at RT for 12 h. The benzene on top of the silica material was decanted and the material washed with benzene (10 ml) two times to wash off unreacted complex. The material was then washed with pentane before it was dried *in vacuo* to receive **$Pt^{II}\_Zn^{II}Ti^{IV}/SiO_2$** as a white solid. The material was subsequently added to a tubular quartz flow-reactor supported with a porous quartz frit. The reactor was heated to 600°C (ramp of 5°C/min) under a steady flow of H_2 and then treated under H_2 at this final temperature for 12 h. The reactor was subsequently evacuated under high vacuum (10^{-5} mbar) while cooling to RT, yielding **PtZnTi/SiO_2** as a black material. Elemental Analysis: Pt, 2.95 wt%; Zn, 1.53 wt%; Ti, 0.58 wt%.

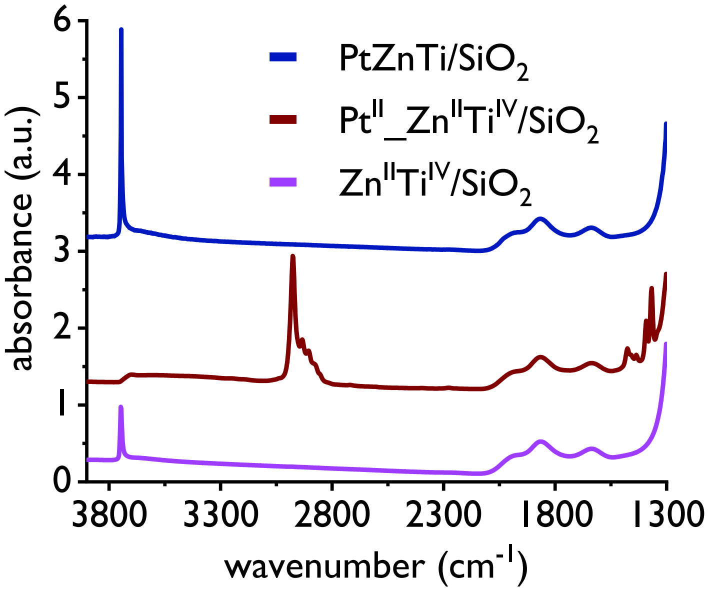

Figure S3. Transmission FTIR spectra of **$Zn^{II}Ti^{IV}/SiO_2$** (purple), **$Pt^{II}\_Zn^{II}Ti^{IV}/SiO_2$** (brown) and **$PtZnTi/SiO_2$** (dark blue) normalized to the $\upsilon_{Si\text{-}O}$ vibrational frequency at 1865 cm^{-1}.

Synthesis of $PtTi/SiO_2$

Ti^{IV}/SiO_2 (0.434 g) was added to a 20 ml screw cap vial. Benzene (about 5 ml) was added slowly while stirring to give a white suspension. **$[Pt(OSi(OtBu)_3)_2(COD)]$** (0.108 g, 0.130 mmol) was added dropwise to the suspension as a colorless solution in benzene (about 5 ml) while stirring (1000 rpm). The resulting white suspension was stirred at RT for 10 h. The benzene on top of the silica material was decanted and the material washed with benzene (5 ml) two times to wash off unreacted complex. The material was then washed with pentane before it was dried *in vacuo* to receive **$Pt^{II}\_Ti^{IV}/SiO_2$** as a white solid. The material was subsequently added to a tubular quartz flow-reactor supported with a porous quartz frit. The reactor was heated to 600°C (ramp of 5°C/min) under a steady flow of H_2 and then treated under H_2 at this final temperature for 9 h. The reactor was subsequently evacuated under high vacuum (10^{-5} mbar) while cooling to RT, yielding **$PtTi/SiO_2$** as a black material. Elemental Analysis: Pt, 3.46 wt%; Ti, 0.59 wt%.

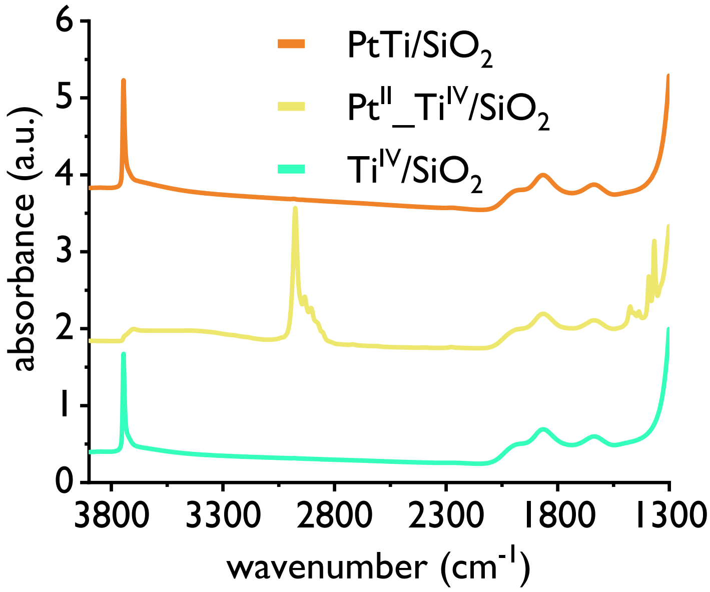

Figure S4. Transmission FTIR spectra of **Ti^{IV}/SiO_2** (turquoise), **Pt^{II}_Ti^{IV}/SiO_2** (yellow) and **$PtTi/SiO_2$** (orange) normalized to the $\upsilon_{Si\text{-}O}$ vibrational frequency at 1865 cm^{-1}.

STEM/EDX

High-angle annular dark-field scanning transmission electron microscope (HAADF-STEM) images were recorded on a FEI Talos F200X instrument operated at 200 keV. Powdered samples were mixed in solid form with a Lacey-C 400 mesh Cu grid inside of a glovebox under an atmosphere of Ar before mounted on a vacuum transfer tomography holder from Fischione Instruments (model #2560) inside the glove box which was subsequently transferred to the chamber of the TEM in the absence of air. Imaging of spent materials was done after exposure to air. All given values for size distributions assume a normal distribution. Some of the overlayed distribution curves are lognormal, indicating that these distributions are better represented by a lognormal distribution. For all materials exactly 300 nanoparticles were counted. The nanoparticle diameter determination to obtain the particle size distribution (PSD) was done by manual measurement with the standard software ImageJ (version 1.52a). The "±" in the depicted particle size distributions indicates the standard deviation of the mean.

PtTi/SiO$_2$

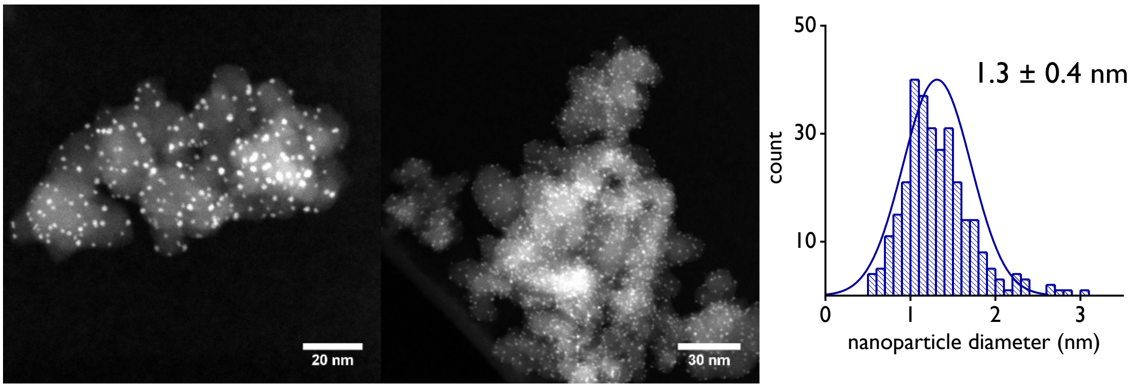

Figure S5. HAADF-STEM images of **PtTi/SiO$_2$** and corresponding particle size distribution.

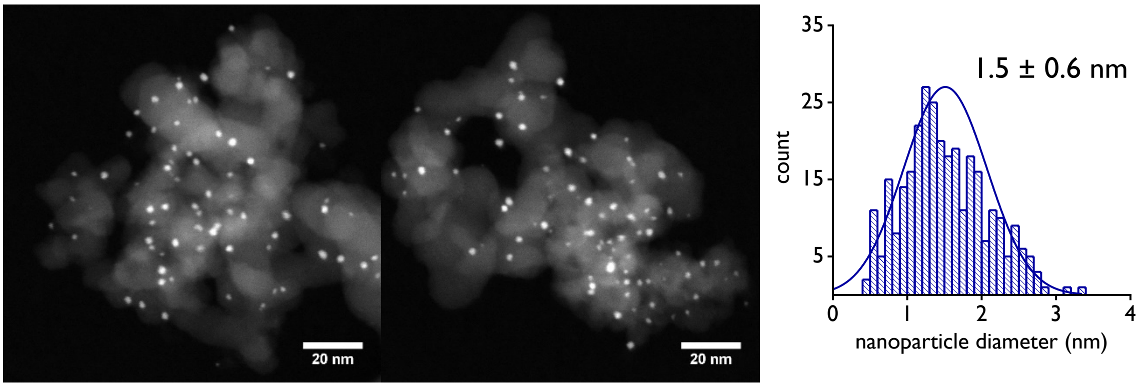

Figure S6. HAADF-STEM images of **PtTi/SiO$_2$** and corresponding particle size distribution after 18.8 h on stream under PDH conditions.

PtZn/SiO$_2$

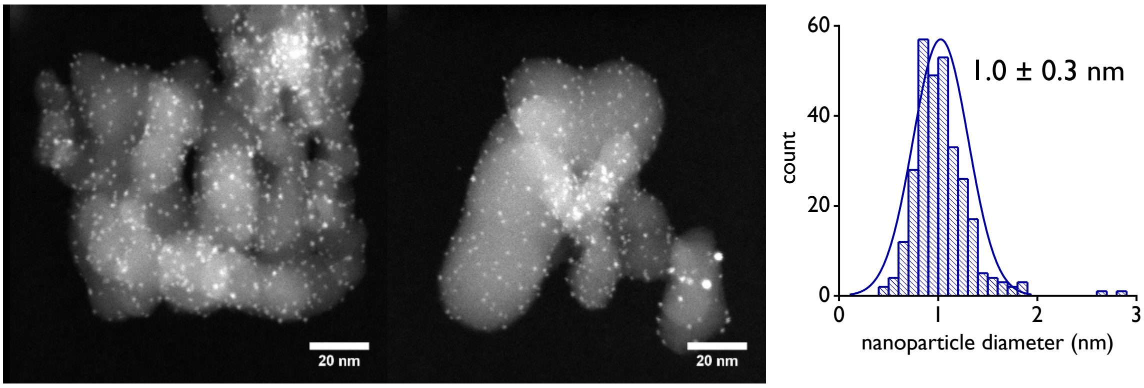

Figure S7. HAADF-STEM images of **PtZn/SiO$_2$** and corresponding particle size distribution.

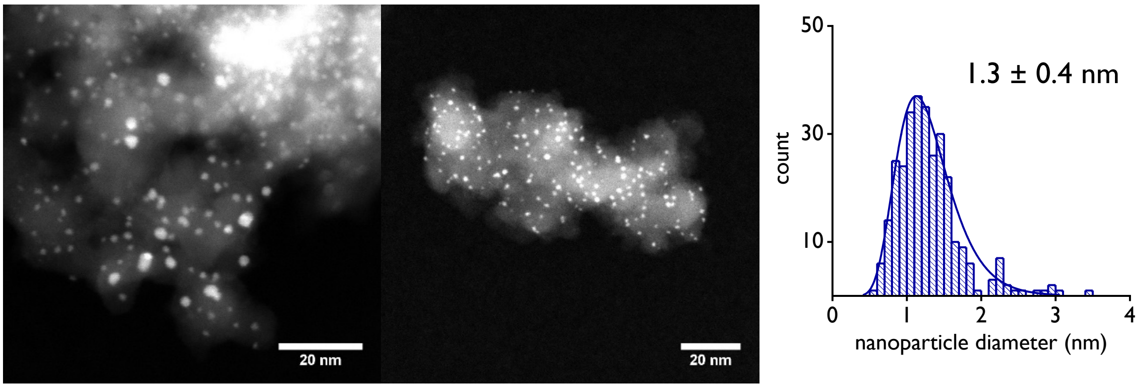

Figure S8. HAADF-STEM images of **$PtZn/SiO_2$** and corresponding particle size distribution (lognormal curve shown) after 108.8 h on stream under PDH conditions.

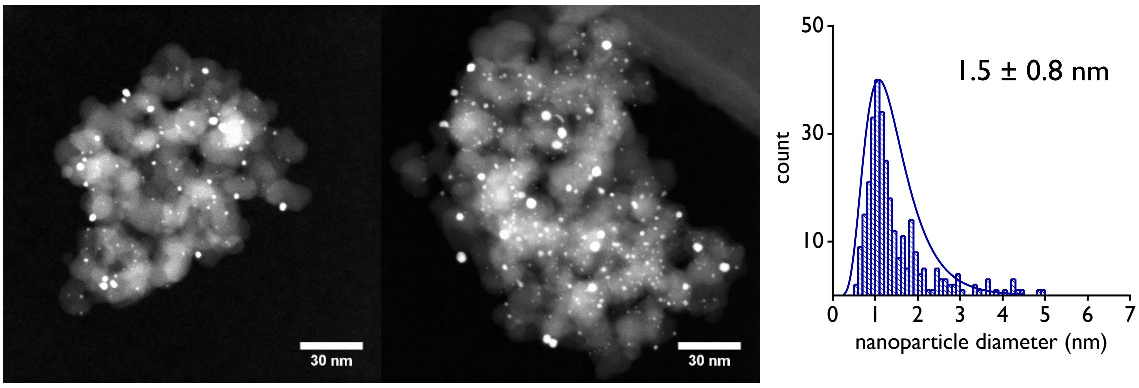

Figure S9. HAADF-STEM images of **$PtZn/SiO_2$** and corresponding particle size distribution (lognormal curve shown) after two long regeneration cycles and a total of 315.1 h on stream under PDH conditions.

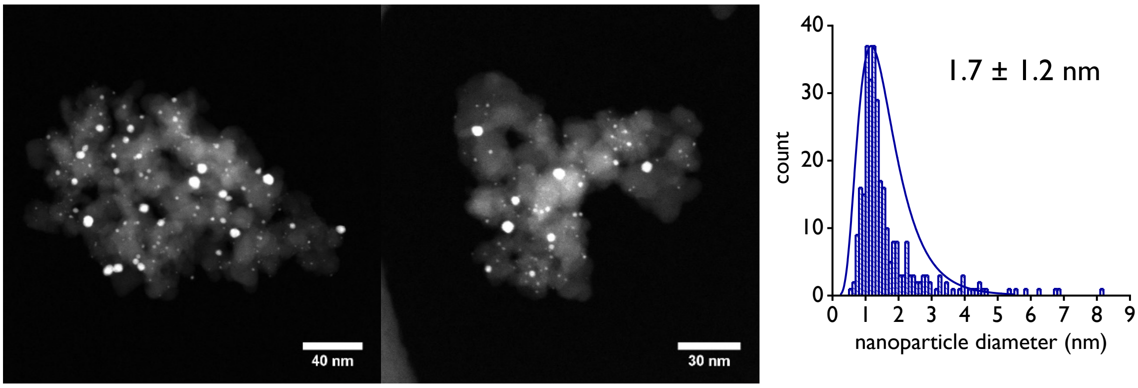

Figure S10. HAADF-STEM images of **$PtZn/SiO_2$** and corresponding particle size distribution (lognormal curve shown) after six short regeneration cycles and a total of 2 h on stream under PDH conditions.

PtZnTi/SiO2

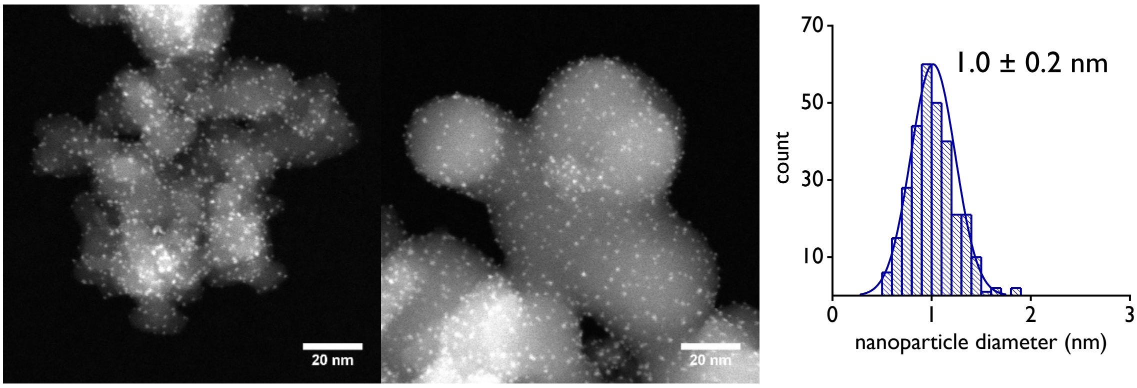

Figure S11. HAADF-STEM images of **PtZnTi/SiO2** and corresponding particle size distribution.

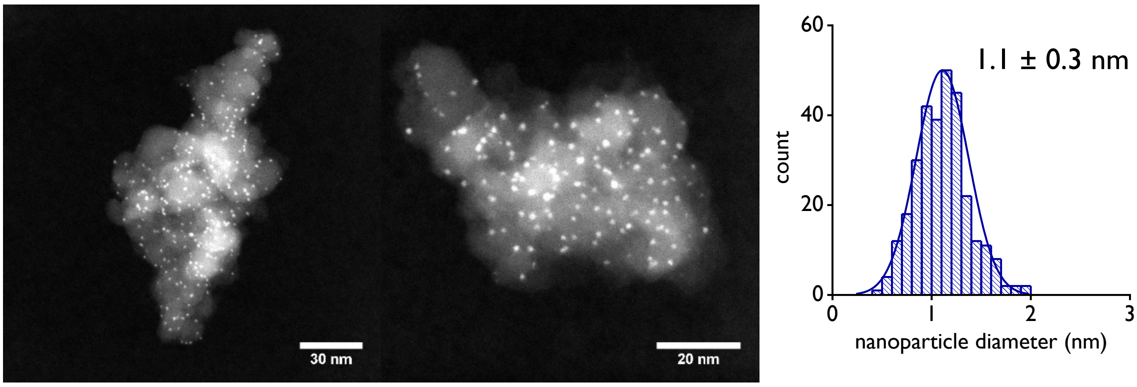

Figure S12. HAADF-STEM images of **PtZnTi/SiO2** and corresponding particle size distribution after 114.4 h on stream under PDH conditions.

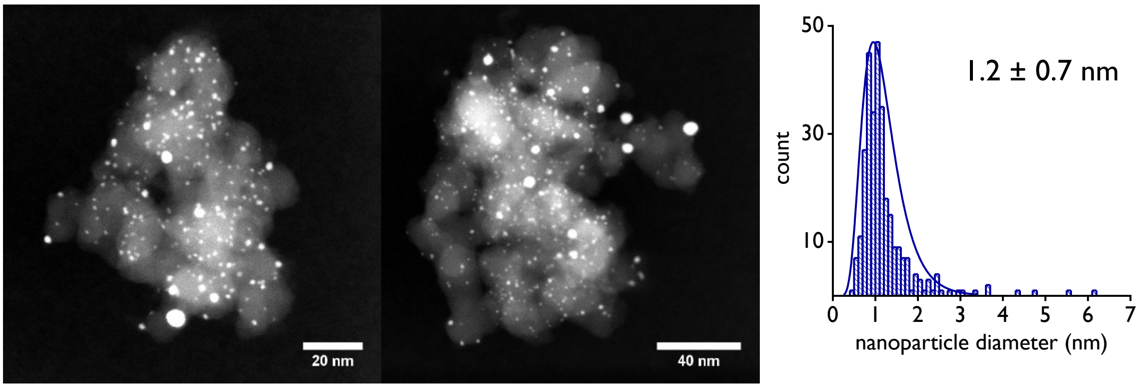

Figure S13. HAADF-STEM images of **PtZnTi/SiO2** and corresponding particle size distribution (lognormal curve shown) after two long regeneration cycles and a total of 221.3 h on stream under PDH conditions.

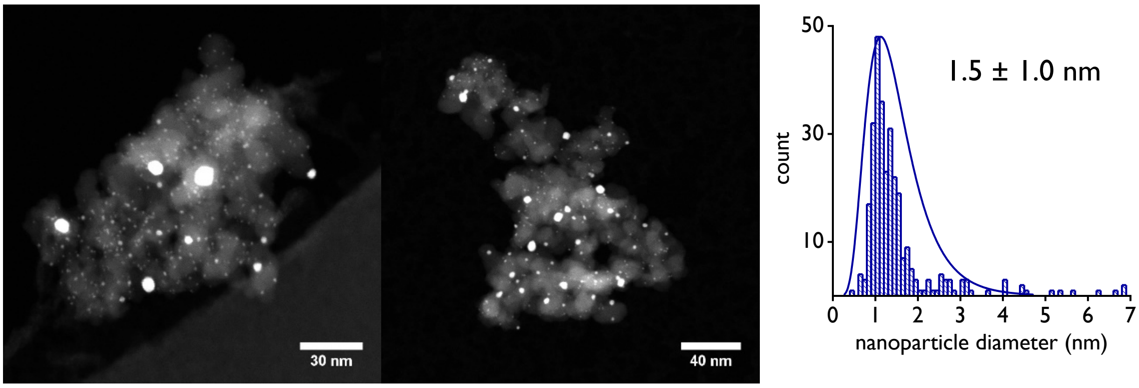

Figure S14. HAADF-STEM images of **PtZnTi/SiO_2** and corresponding particle size distribution (lognormal curve shown) after six short regeneration cycles and a total of 2 h on stream under PDH conditions.

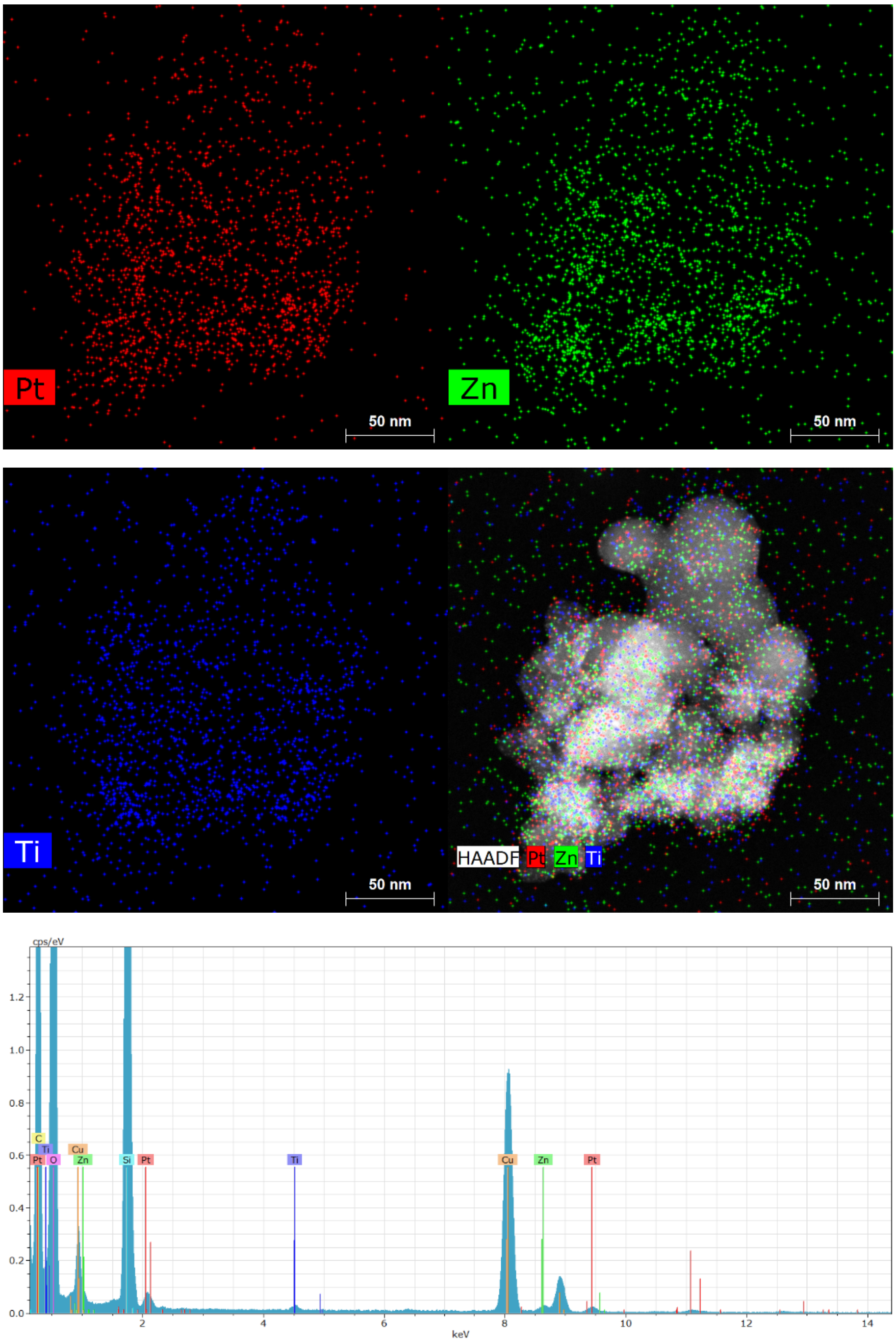

Figure S15. HAADF-STEM/EDX image of **PtZnTi/SiO_2** and corresponding EDX spectrum with energy resolution of ≤ 136 eV (for Mn Kα). Pt, Zn and Ti are well dispersed over the SiO_2 support.

FTIR CO Adsorption

Self-supporting pellets of **Ti^{IV}/SiO$_2$**, **Ti/SiO$_2$_H$_2$**, **PtTi/SiO$_2$** and **PtZnTi/SiO$_2$** were freshly prepared, submitted to an atmosphere of CO and analyzed by transmission Fourier transform infrared (FTIR) spectroscopy at 2 cm^{-1} resolution. For the Pt containing materials pellets of ca. 10-11 mg were prepared and CO pressures (ca. 10-11 mbar) were kept low to prevent saturation of the spectra. For the Ti only containing materials, pellets of 10-11 mg were prepared and submitted to ca. 120 mbar CO pressure. After placing a sample under high vacuum (10^{-5} mbar), the pellet was exposed to CO and subsequently analyzed by FTIR spectroscopy. All pressures were maintained for a minimum of 5 minutes prior to measurement.

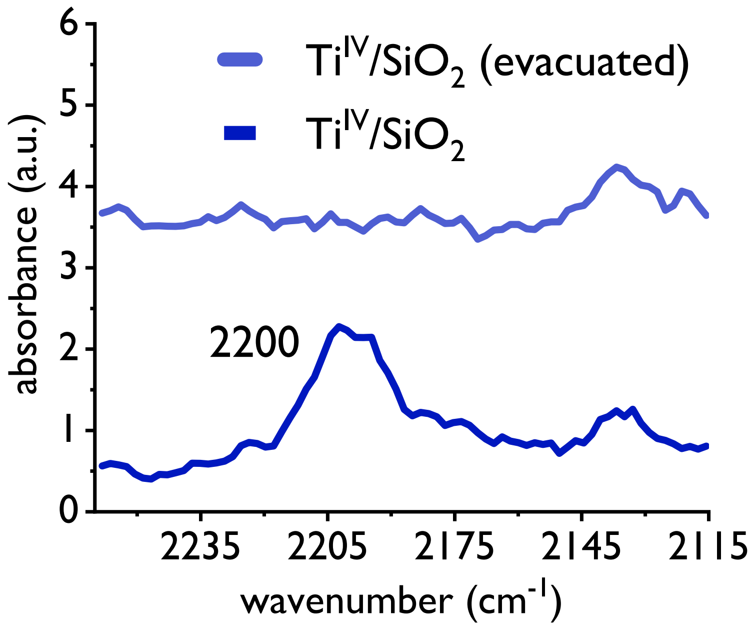

Figure S16. ^{12}CO adsorption on **Ti^{IV}/SiO$_2$** before and after evacuation of CO. The spectra are background corrected by subtraction.

H_2 and CO Chemisorption

Chemisorption experiments were performed using a BEL JAPAN BELSORP-MAX. Around 222 mg (**PtTi/SiO$_2$**) and 180 mg (**PtZnTi/SiO$_2$**) of the materials were loaded into cells in an Ar-filled and solvent-free glovebox. Pretreatment for H_2 and CO chemisorption measurements involved heating the samples at 300°C for 3 h under dynamic vacuum. Adsorption experiments were performed at 40°C under isothermal conditions using a circulating thermostat bath. The chemisorption curves were fitted with the following Langmuir adsorption isotherms:

$$Q_{H_2}(P) = Q_{H_2 sat} \cdot \frac{\sqrt{K_{H_2}\frac{p}{p_0}}}{1+\sqrt{K_{H_2}\frac{p}{p_0}}} \quad \text{or} \quad Q_{CO}(P) = Q_{CO\ sat} \cdot \frac{K_{CO}\frac{p}{p_0}}{1+K_{CO}\frac{p}{p_0}}$$

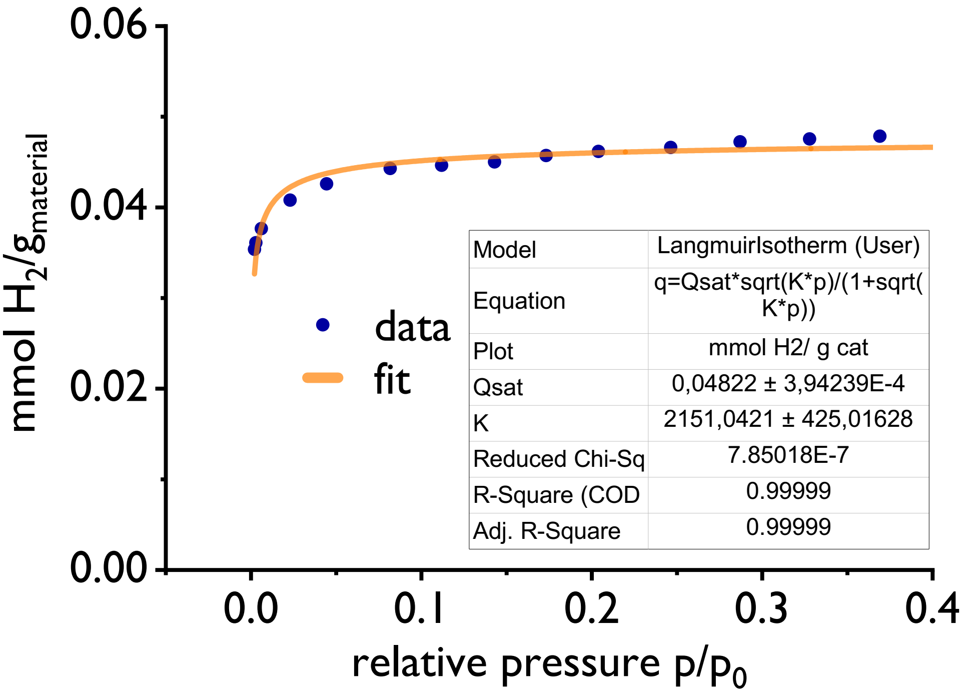

Figure S17. H_2 chemisorption data and fit for **PtTi/SiO$_2$**.

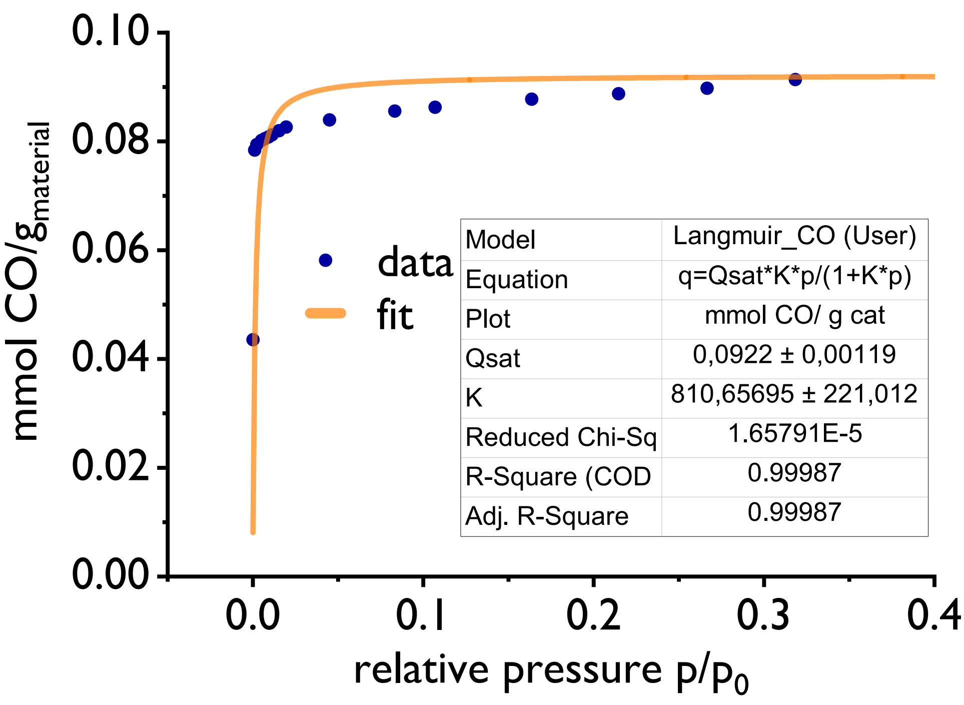

Figure S18. CO chemisorption data and fit for **PtTi/SiO_2**.

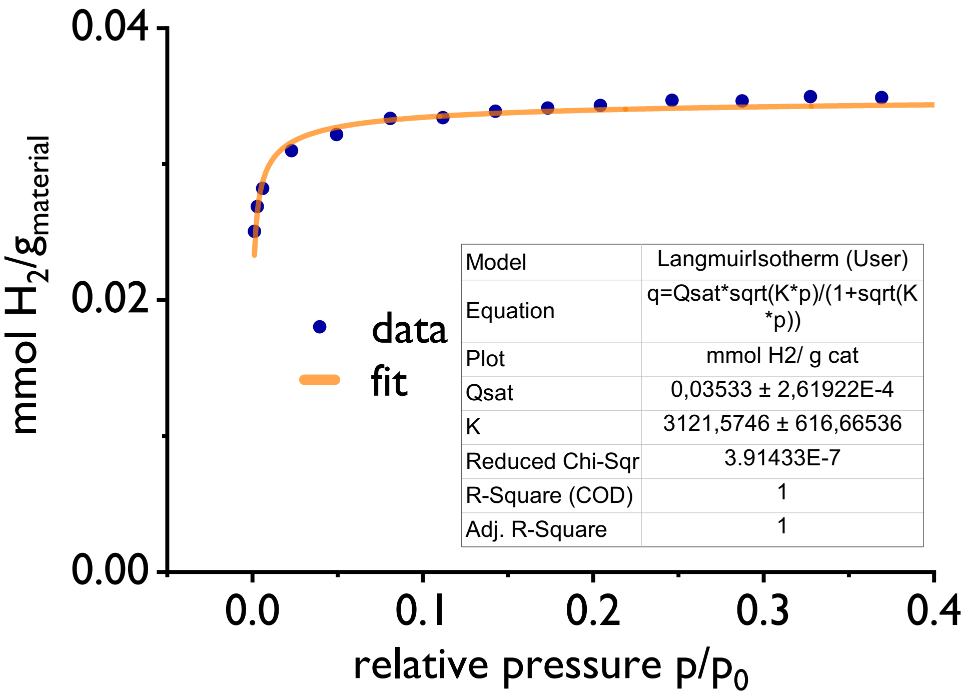

Figure S19. H_2 chemisorption data and fit for **PtZnTi/SiO_2**.

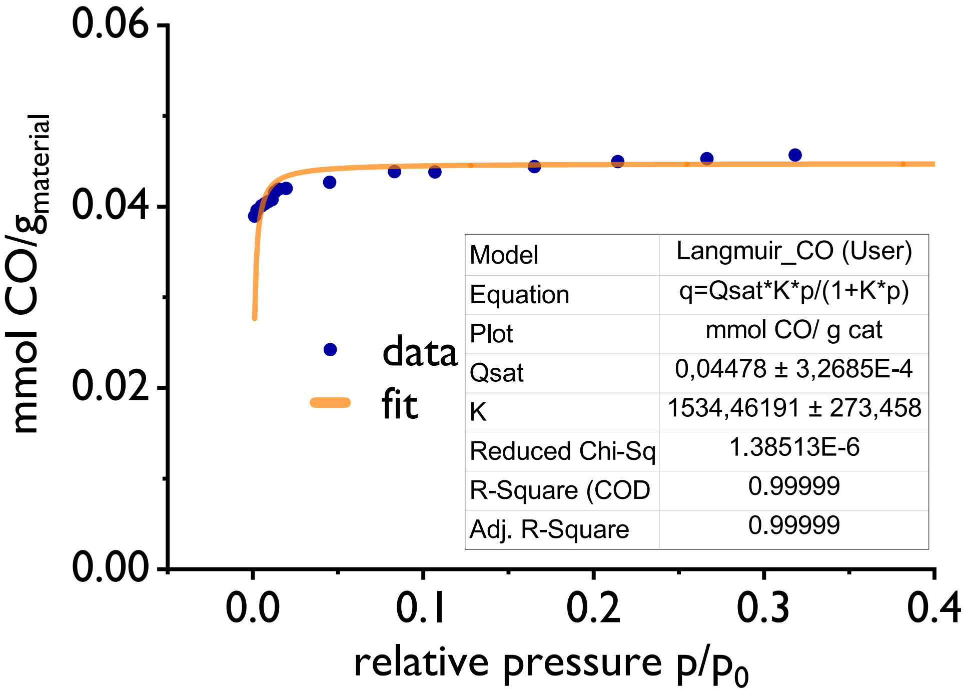

Figure S20. CO chemisorption data and fit for **PtZnTi/SiO$_2$**.

Table S1. H_2 and CO chemisorption data for **PtTi/SiO$_2$** and **PtZnTi/SiO$_2$**.

material	PtTi/SiO$_2$	PtZnTi/SiO$_2$
$Q_{CO\ sat}\left(\frac{mmol\ CO}{g_{Pt}}\right)$	2.7	1.5
$Q_{H_2 sat}\left(\frac{mmol\ H_2}{g_{Pt}}\right)$	1.4	1.2

XAS

XAS measurements were carried out at the Zn K-edge and Pt L_{III}-edge at the SuperXAS beamline at SLS (PSI, Villigen, Switzerland). The storage ring was operated at 2.4 GeV in top-up mode with a ring current of around 400 mA. The incident photon beam provided by a 2.9 T superbend magnet was selected by a liquid nitrogen cooled Si(111) quick-EXAFS monochromator and the rejection of higher harmonics and focusing were achieved by a rhodium-coated double focusing mirror at 2.9 mrad for both Pt L_{III} and Zn K edges. The beamsize on the sample was 400 x 150 µm. During measurements, the monochromator was rotating with 1 Hz frequency and X-ray absorption spectra were collected in transmission mode using ionization chambers (filling of 2.5 bar for both Pt L_{III} and Zn K measurements) especially developed for quick data collection with 2 MHz frequency.[7] Spectra were collected for 3-10 min and averaged. The beamline energy was calibrated with a Pt reference foil to the Pt L_{III}-edge energy at 11564.0 eV. To avoid contact with air, all samples were sealed in a glovebox. For *ex situ* samples, pressed pellets (with optimized thickness for transmission detection) were placed in two aluminized plastic bags (Polyaniline (15 µm), polyethylene (15 µm), Al (12 µm), polyethylene (75 µm) from Gruber-Folien GmbH & Co. KG, Straubing, Germany) using an impulse sealer inside a glovebox; one sealing layer was removed immediately before the measurements. Data processing was done by standard procedures using the ProXASGui software developed at the SuperXAS beamline, PSI, Villigen. The program package Demeter was used for data analysis.[8] The $S0^2$ value for the Pt L_{III}-edge (0.82 ± 0.02) was obtained by fitting of Pt foil.[9] Coordination numbers were fixed for this fit. For all Pt L_{III} edge fits coordination numbers were forced to be positive. The $S0^2$ value for the Zn K-edge (0.90 ± 0.09) was obtained by fitting of Zn foil. For the Zn K edge the background was fit in order to remove unrealistic low frequency oscillation from the data. In all cases, a Zn hexagonal close-packed structure was used for the fits, lattice parameters were shrank by 8% to adjust for the small nanoparticle sizes and either the core atom or nearest neighbors replaced by another element of interest to create different distances.

Ex situ Zn K edge XANES

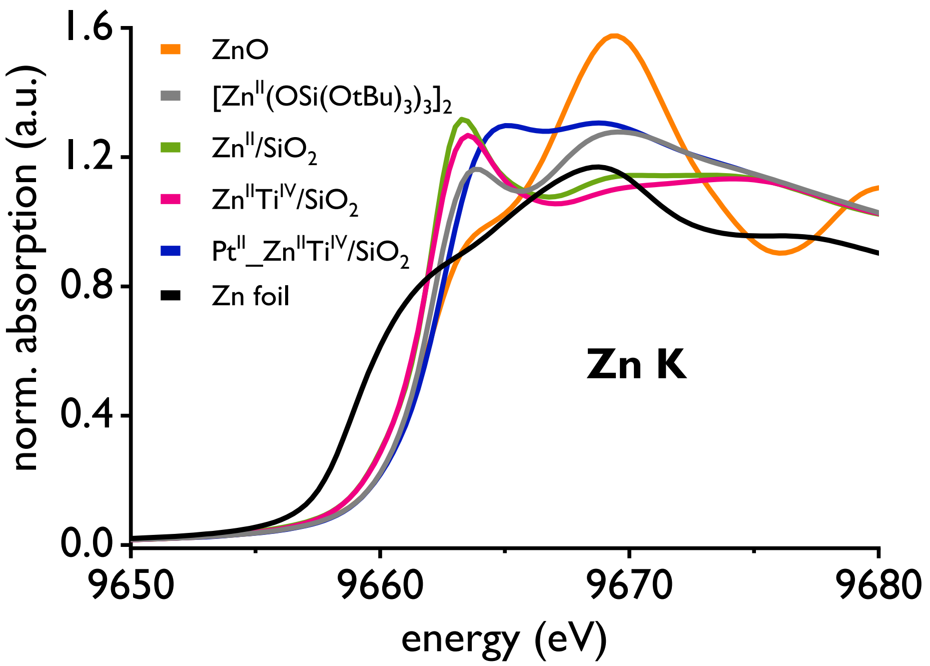

Figure S21. *Ex situ* XANES spectra at the Zn K edge of several Zn^{II} containing materials and Zn foil.

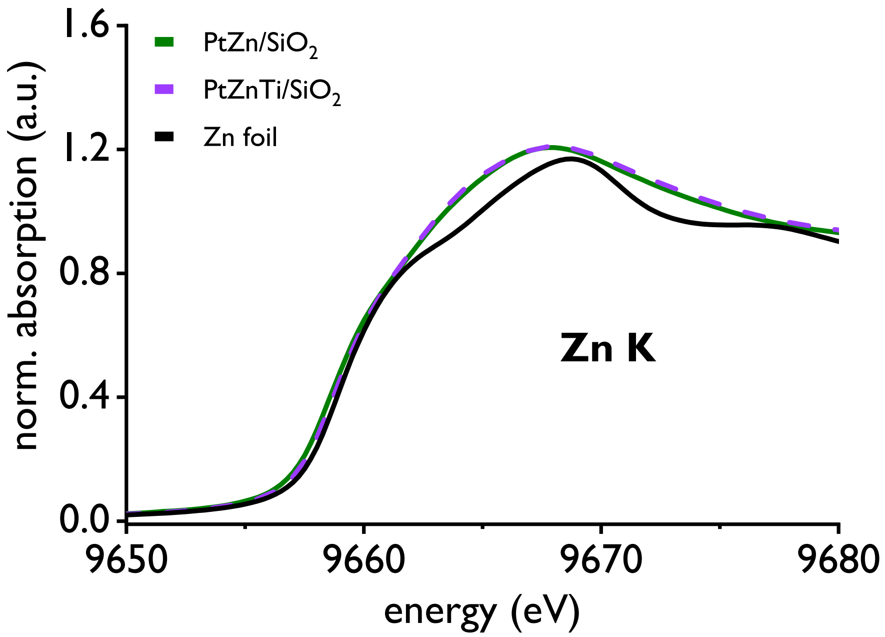

Figure S22. *Ex situ* XANES spectra at the Zn K edge of two PtZn containing materials after reduction under a flow of H_2 at 600°C and Zn foil.

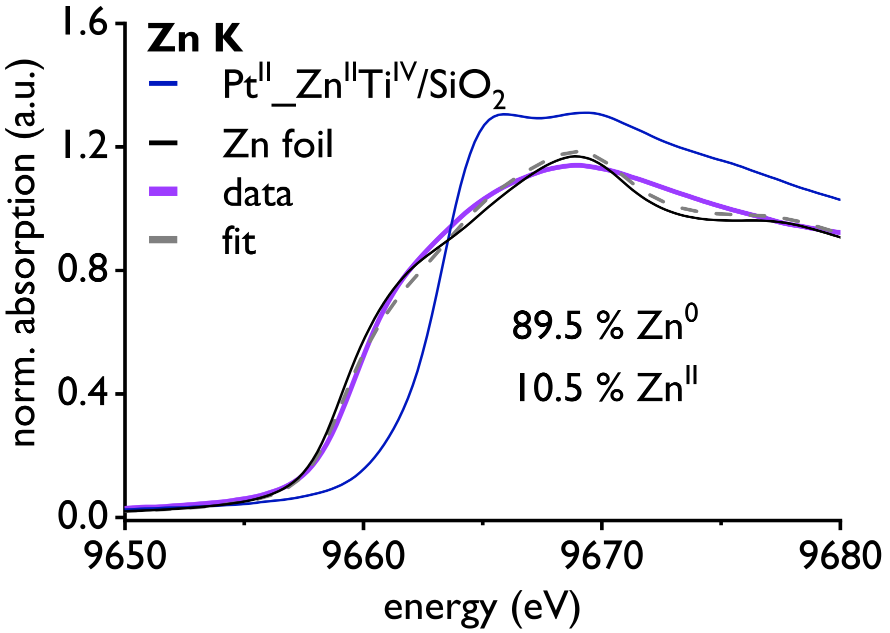

Figure S23. Linear combination fit (LCF) of **PtZnTi/SiO₂** (purple line, after TPR as depicted in Figure 2 A of the main text) at the Zn K edge using the initial state **Pt^{II}_$Zn^{II}Ti^{IV}/SiO_2$** of the TPR experiment and Zn foil as standards. The R factor of the fit is 0.002.

Ex situ Pt L_{III} edge XANES

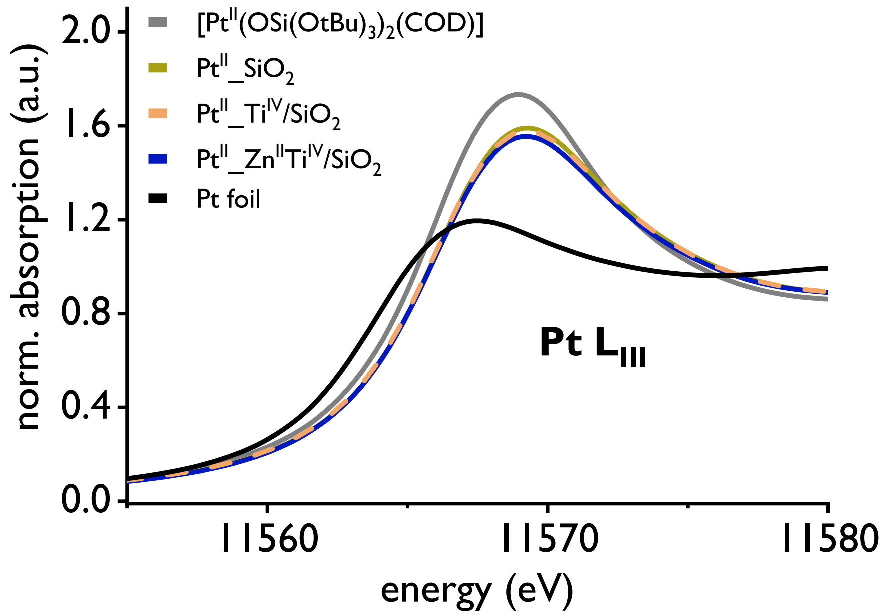

Figure S24. *Ex situ* XANES spectra at the Pt L_{III} edge of several Pt^{II} containing materials and Pt foil.

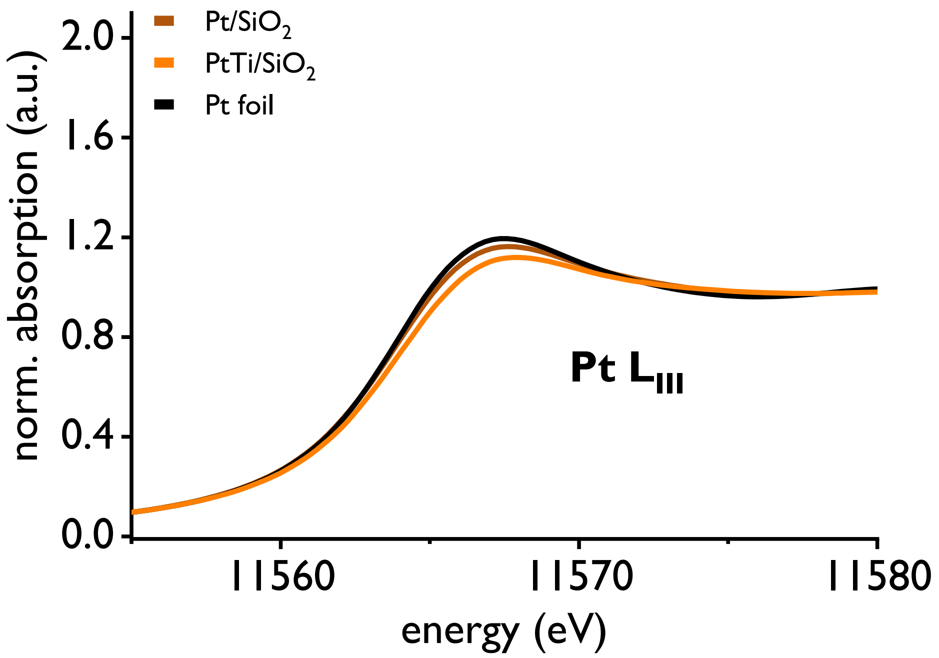

Figure S25. *Ex situ* XANES spectra at the Pt L_{III} edge of two Pt containing materials after reduction under a flow of H_2 at 600°C and Pt foil.

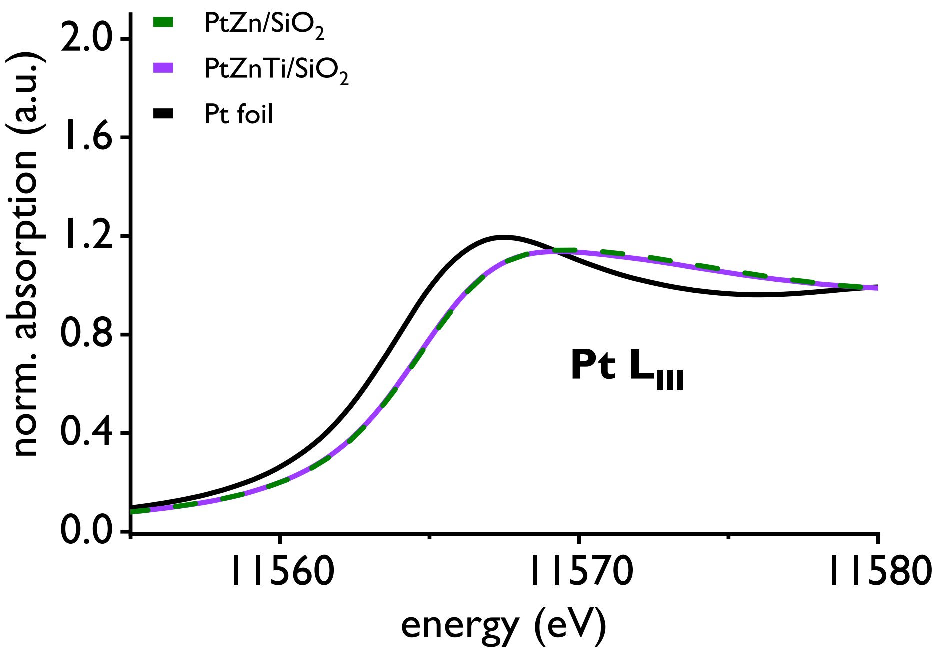

Figure S26. *Ex situ* XANES spectra at the Pt L_{III} edge of two PtZn containing materials after reduction under a flow of H_2 at 600°C and Pt foil.

In situ Regeneration Study at the Zn K and Pt L_{III} Edge

For *in situ* measurements, catalyst beds of appropriate mass were supported with quartz wool in 3 mm quartz capillaries. Gas flow and composition were controlled using Bronkhorst mass flow controllers and a back-pressure regulator. For the PtZn and PtZnTi materials different conditions were applied during the regeneration cycles as the allocated beamtimes at the SuperXAS beamline at PSI were approximately 2 years apart from each other and setups and amount of time allocated were different, which required adjustments of the experimental procedures. Nevertheless, the conditions used to acquire the presented datasets should provide comparable information to showcase the effects of high temperature oxidizing (O_2) and reducing (H_2) conditions after a deactivation phase under PDH conditions. The important treatment parameters are summarized in Table S2 for both materials. For the PtZnTi sample TPR (of **Pt^{II}_$Zn^{II}Ti^{IV}/SiO_2$**) was followed by PDH/oxidation/reduction phases while for the PtZn material the TPR treatment was not preceding the regeneration phases but pre-reduced **$PtZn/SiO_2$** was directly submitted to PDH conditions followed by the regeneration phases. Ar, H_2 and propane were purified by passing through a column with molecular sieves and Cu-0226 S (Q5) catalyst prior to introduction to the XAS quartz capillary. O_2/Ar was purified by passing through a column with molecular sieves. Pressures were generally kept at ca. 1.3 atmospheres to avoid leaking into the capillaries while heating ramps were set to 10°C/min while cooling ramps were at 40°C/min for the PtZnTi material. During the time when PtZn was measured no precise heating ramps were available and the heating was approximately 20°C/min.

Table S2. Parameters for the *in situ* regeneration study of $PtZn/SiO_2$ and $PtZnTi/SiO_2$.

treatment	TPR	PDH	Oxidation	Reduction
			**PtZnTi material**	
Flowrate [ml/min]	6/4	20	20	6/4
Gas	H_2/Ar	C_3H_8	5% and 20% O_2/Ar	H_2/Ar
Temperature [°C]	600	550	600	600
Duration [h]	1	4	1 and 2	1.5
			**PtZn material**	
Flowrate [ml/min]	-	10/40	20	10
Gas	-	C_3H_8/Ar	20% O_2/Ar	H_2
Temperature [°C]	-	550	550	550
Duration [h]	-	4	1	1

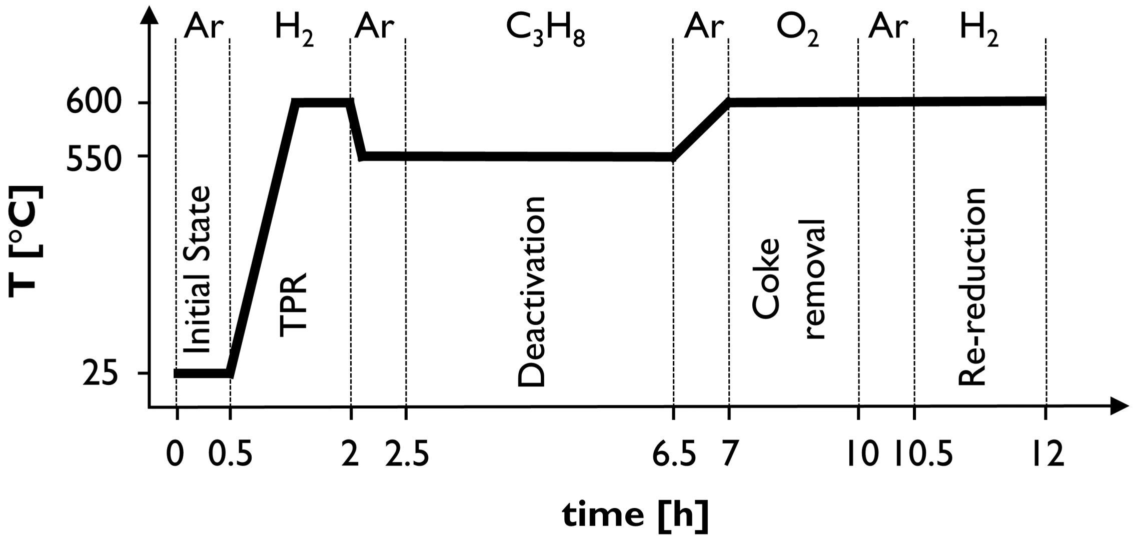

Figure S27. Schematic presentation of a typical regeneration experiment of **Pt^{II}_$Zn^{II}Ti^{IV}/SiO_2$**. For $PtZn/SiO_2$ the first part (TPR) was skipped and PDH conditions applied to the pre-reduced material.

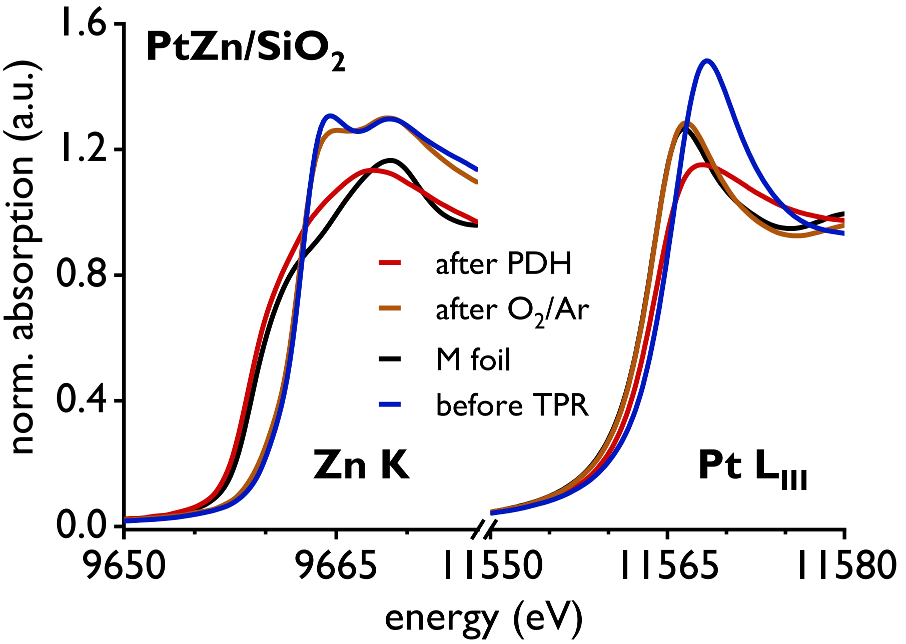

Figure S28. *In situ* spectra of **$PtZn/SiO_2$** at the Zn K and the Pt L_{III} edge after PDH conditions were applied (red line) and evolution under O_2/Ar (brown line). It can be observed that Zn is clearly oxidized to Zn^{II} being very similar to the Zn species in **Pt^{II}_Zn^{II}/SiO_2** before TPR conditions are applied. For Pt a change towards more similarity

with Pt foil can be observed, clearly indicating that Pt and Zn dealloy while Pt is not significantly oxidized, which could be explained by the formation of a ZnO layer around the particles or simply by not harsh enough conditions to oxidize Pt to Pt^{II}.

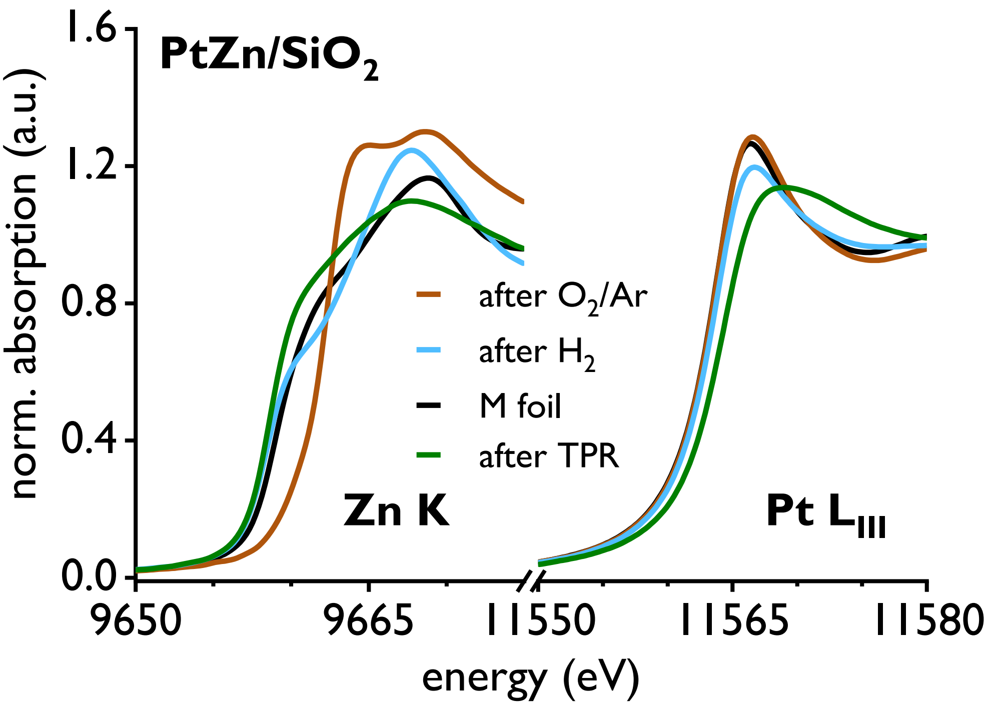

Figure S29. *In situ* spectra of **PtZn/SiO₂** at the Zn K and the Pt L_{III} edge after oxidizing conditions (O_2/Ar) were applied (brown line) and evolution under H_2 (blue line). It can be observed that Zn is re-reduced under H_2. However, the species after the reductive treatment does not resemble the structure after a TPR treatment (which resembles the pristine catalyst before PDH conditions are applied). A similar trend can be observed for the Pt L_{III} edge in that only slight changes can be observed after the reductive treatment, indicating that Pt and Zn do not fully realloy after the H_2 treatment but that Pt stays rather metallic and that the state of Zn does not resemble a fully alloyed state.

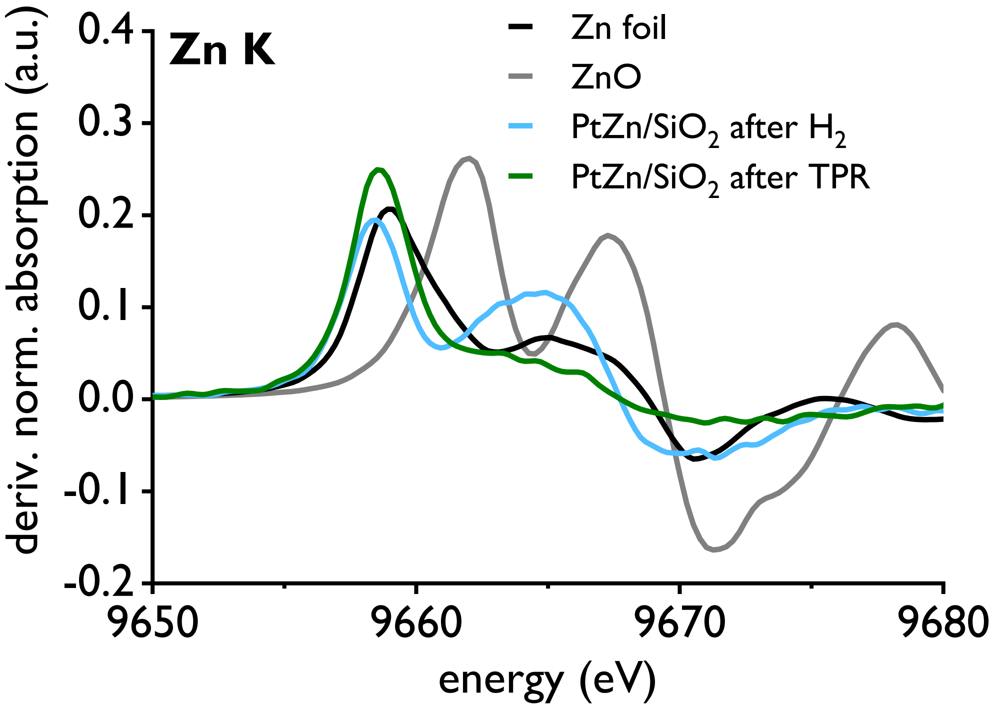

Figure S30. First derivative *in situ* spectrum of **PtZn/SiO$_2$** at the Zn K edge after reducing conditions (H_2) were applied (blue line) compared to the material after TPR treatment (green line) to better illustrate the changes in spectral features. Comparing the spectra to Zn foil as well as ZnO it can be seen that after the H_2 treatment **PtZn/SiO$_2$** rather shows features of Zn foil and **PtZn/SiO$_2$** and no features of ZnO. The data shows that Zn is mostly re-reduced but the nature of Zn and with that the nature of the nanoparticles after H_2 treatment changes compared to the nature after TPR.

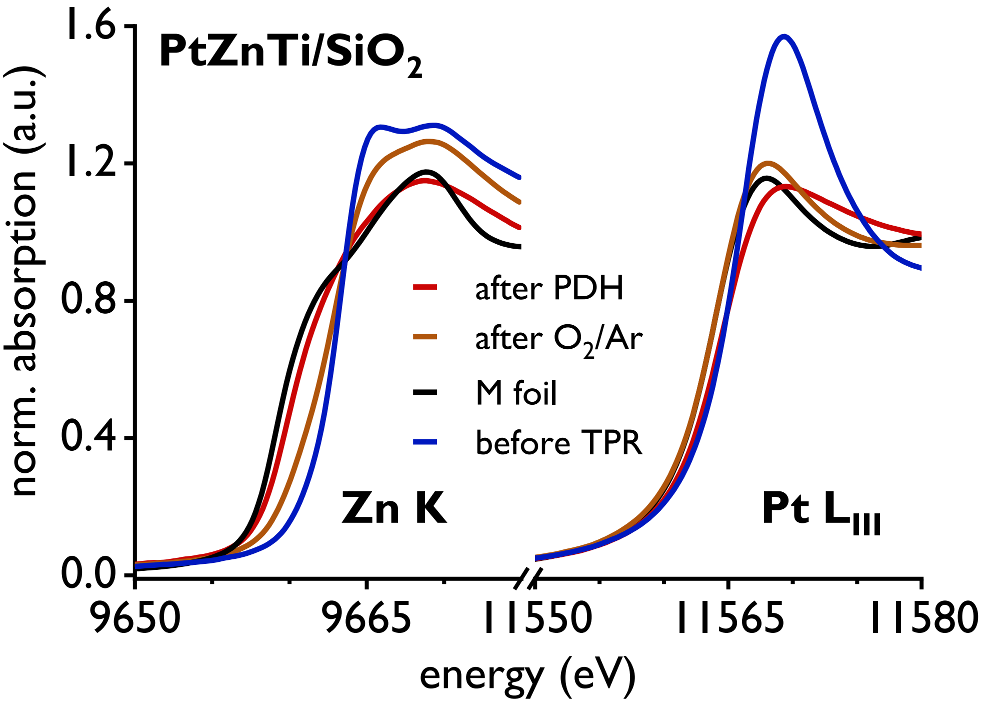

Figure S31. *In situ* spectra of **PtZnTi/SiO₂** at the Zn K and the Pt L_{III} edge after PDH conditions were applied (red line) and evolution under O_2/Ar (brown line). It can be observed that Zn is partially oxidized to Zn^{II} while not as distinct as in the case of the PtZn material. For Pt a similar change compared to the PtZn material can be observed, indicating that Pt after O_2/Ar is more similar to Pt foil and thus that Pt and Zn (partially) dealloy while Pt is not significantly oxidized during the oxidizing treatment.

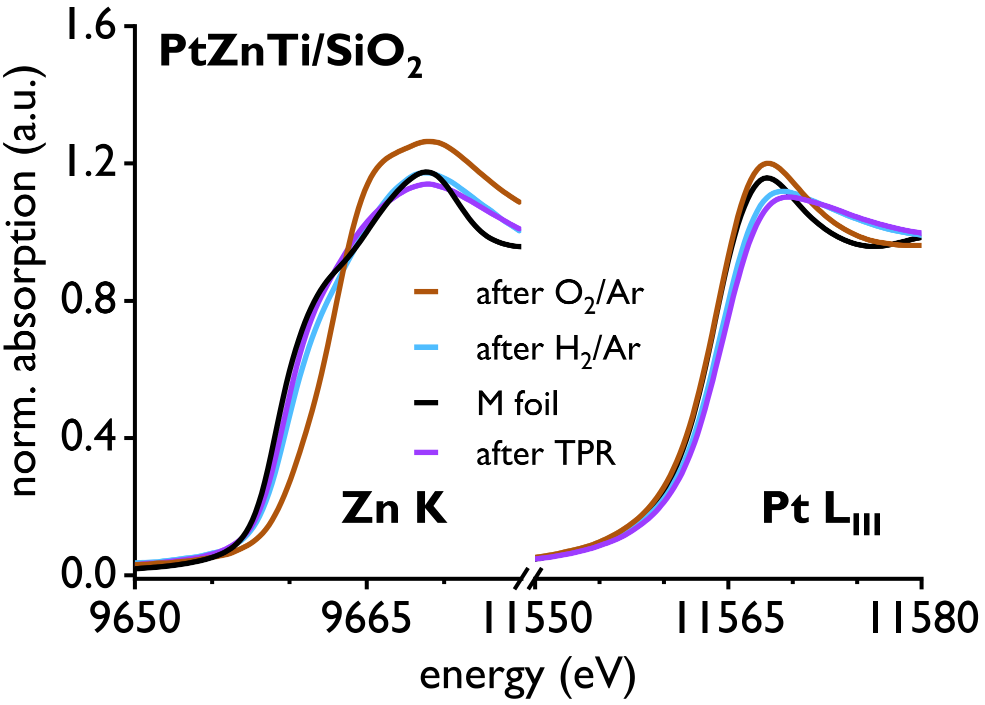

Figure S32. *In situ* spectra of **PtZnTi/SiO₂** at the Zn K and the Pt L_{III} edge after oxidizing conditions (O_2/Ar) were applied (brown line) and evolution under H_2 (blue line). It can be observed that Zn is re-reduced under H_2 and resembles almost the same Zn K edge structure after as the material after a TPR treatment (which resembles the pristine catalyst before PDH conditions are applied). A similar trend can be observed at the Pt L_{III} edge in that the structure of the spectrum resembles very closely the one after TPR which is different to the PtZn material where a more metallic state is observed for Zn (See Figure S29).

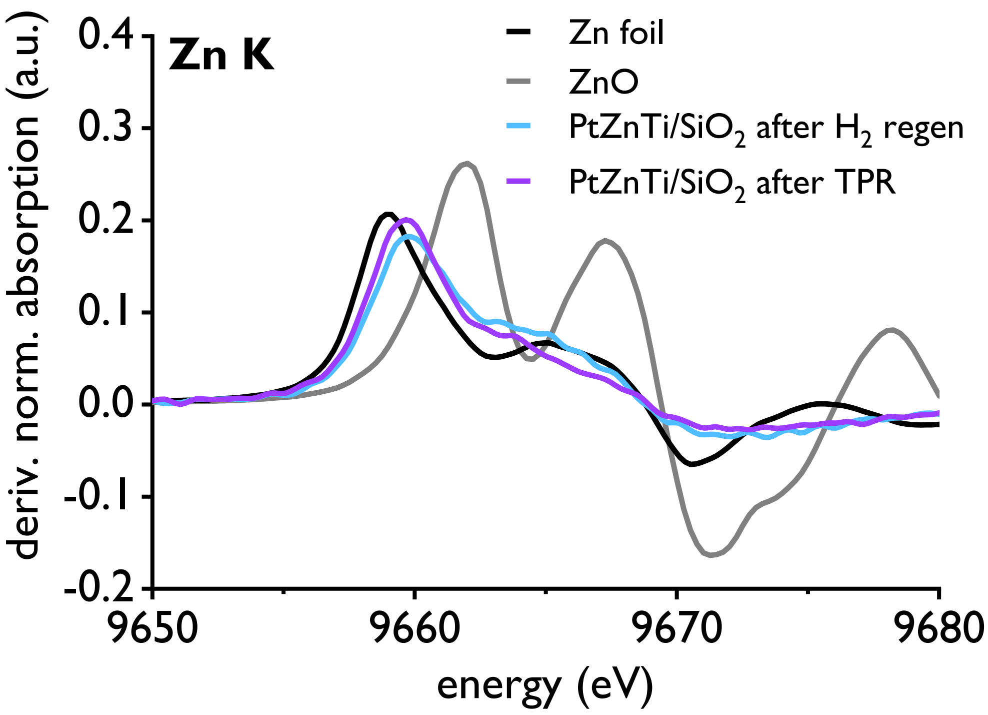

Figure S33. First derivative *in situ* spectrum of **PtZnTi/SiO$_2$** at the Zn K edge after reducing conditions (H_2) were applied (blue line) compared to the material after TPR treatment (purple line) to better illustrate the changes in spectral features. Comparing the spectra to Zn foil as well as ZnO it can be seen that **PtZnTi/SiO$_2$** after the H_2 treatment indeed very closely resembles **PtZnTi/SiO$_2$** after TPR (compare Figure S32), indicating that Pt and Zn re-alloy almost fully after the regeneration treatment and resemble a similar nature as the nanoparticles after TPR.

EXAFS Analysis at the Pt L_{III} Edge

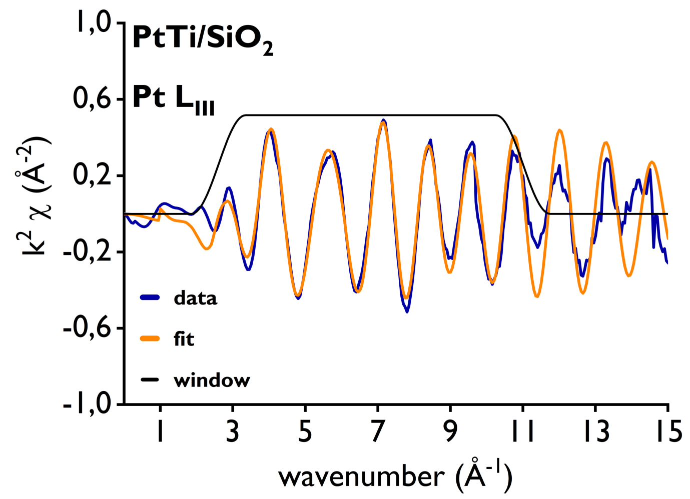

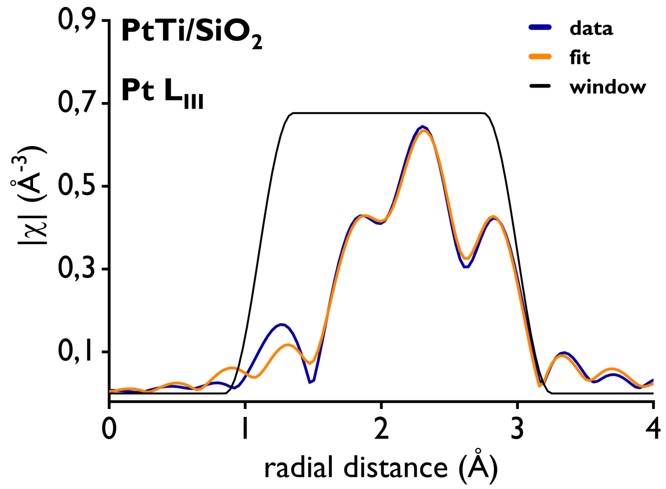

Figure S34. EXAFS data and fit of **$PtTi/SiO_2$** at the Pt L_{III} edge in k-space (upper) and R-space (lower). See Table S3 regarding details of the fit.

Table S3. EXAFS fit parameters for **$PtTi/SiO_2$** at the Pt L_{III} edge. $S0^2$=0.82; k range 2.6-11 Å; R range 1.1-3 Å; k weight 2; Hanning window dk=1.5; Hanning window dr=0.5; R factor 0.015.

Path	N	R, Å	σ^2, Å^2	ΔE_0, eV
Pt-Ti_1	0.9 +/- 0.4	2.46 +/- 0.04		
Pt-Ti_2	1.4 +/- 0.7	2.69 +/- 0.05	0.0066 +/- 0.0024	3.7 +/- 2.1
Pt-Pt	6.6 +/- 1.1	2.67 +/- 0.02		

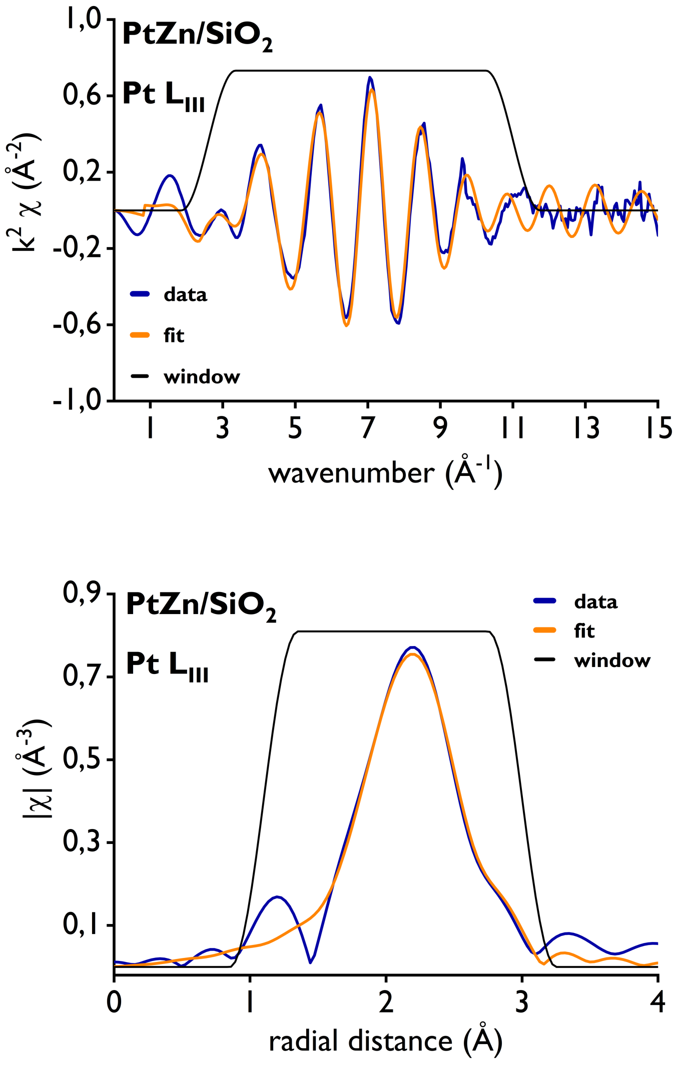

Figure S35. EXAFS data and fit of **$PtZn/SiO_2$** at the Pt L_{III} edge in *k*-space (upper) and *R*-space (lower). See Table S4 regarding details of the fit.

Table S4. EXAFS fit parameters for **$PtZn/SiO_2$** at the Pt L_{III} edge. $S0^2$=0.82; k range 2.6-11 Å; R range 1.1-3 Å; k weight 2; Hanning window dk=1.5; Hanning window dr=0.5; R factor 0.021.

Path	N	R, Å	σ^2, Å^2	ΔE_0, eV
Pt-Zn_1	2.4 +/- 0.8	2.50 +/- 0.09		
Pt-Zn_2	2.1 +/- 0.7	2.63 +/- 0.13	0.0073 +/- 0.0062	2.7 +/- 5.5
Pt-Pt	2.9 +/- 0.9	2.67 +/- 0.06		

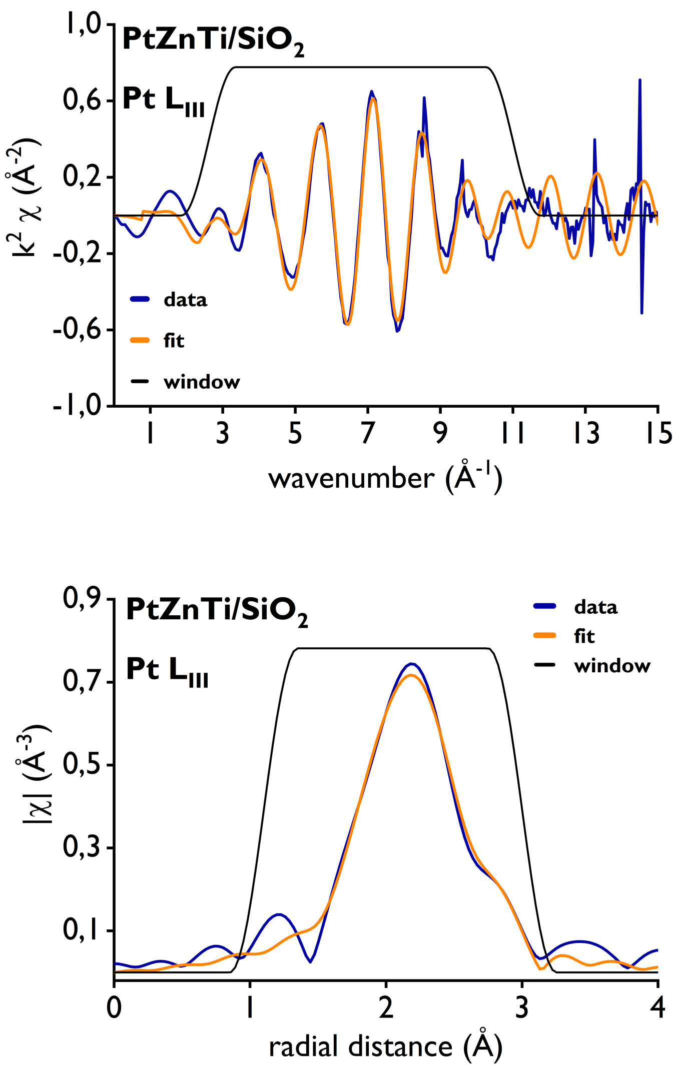

Figure S36. EXAFS data and fit of **PtZnTi/SiO₂** at the Pt L_{III} edge in *k*-space (upper) and *R*-space (lower). See Table S5 regarding details of the fit.

Table S5. EXAFS fit parameters for **PtZnTi/SiO₂** at the Pt L_{III} edge. $S0^2$=0.82; k range 2.6-11 Å; R range 1.1-3 Å; k weight 2; Hanning window dk=1.5; Hanning window dr=0.5; R factor 0.021.

**Path**	**N**	**R, Å**	**σ^2, Å^2**	**ΔE_0, eV**
Pt-Zn_1	2.1 +/- 0.6	2.49 +/- 0.07		
Pt-Zn_2	1.6 +/- 0.5	2.63 +/- 0.11	0.0060 +/- 0.0053	2.3 +/- 4.8
Pt-Pt	3.0 +/- 1.2	2.66 +/- 0.05		

EXAFS Analysis at the Zn K Edge

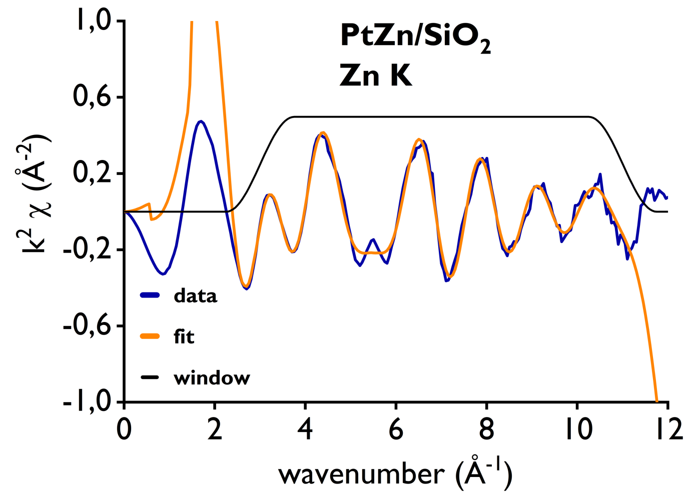

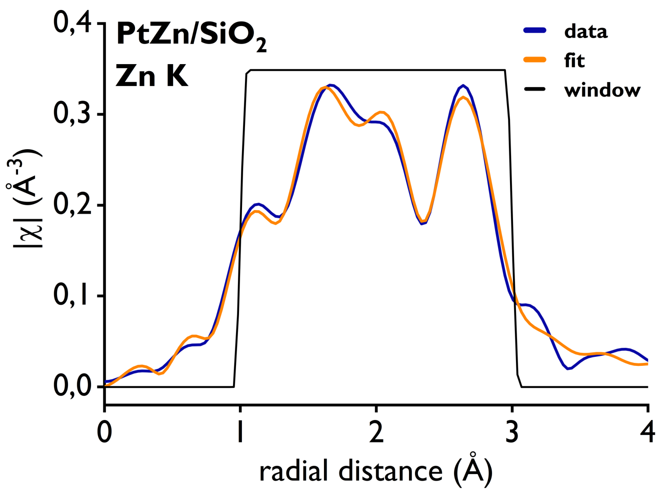

Figure S37. EXAFS data and fit of **$PtZn/SiO_2$** at the Zn K edge in *k*-space (upper) and *R*-space (lower). See Table S6 regarding details of the fit.

Table S6. EXAFS fit parameters for **$PtZn/SiO_2$** at the Zn K edge. $S0^2$=0.90; k range 3.0-11 Å; R range 1-3 Å; k weight 1, 2 and 3; Hanning window dk=1.5; Hanning window dr=0.1; R factor 0.012. Values without errors are fixed.

**Path**	**N**	**R, Å**	**σ^2, Å^2**	**ΔE_0, eV**
Zn-O	3.0 +/- 1.2	1.95 +/- 0.15	0.0274 +/- 0.0842	
Zn_1-Pt	1.8 +/- 0.7	2.490		1.2
Zn_2-Pt	2.1 +/- 0.6	2.630	0.0081 +/- 0.0128	
Zn-Zn	0.5 +/- 0.5	2.82 +/- 0.15		

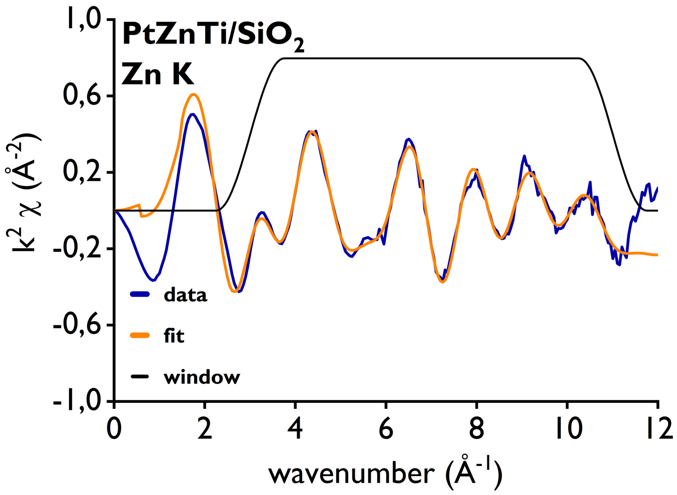

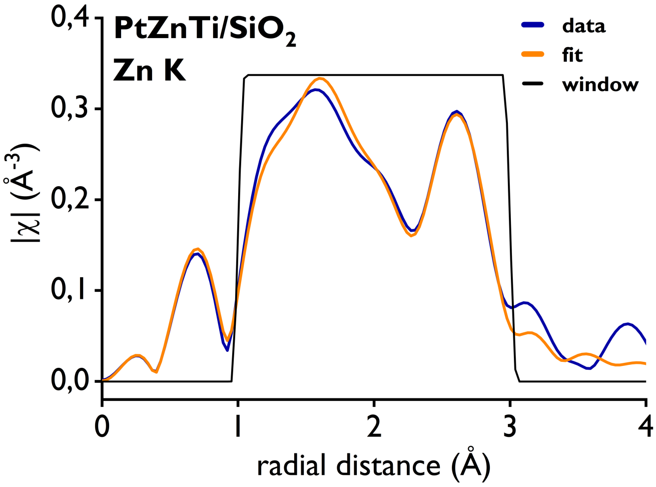

Figure S38. EXAFS data and fit of **PtZnTi/SiO$_2$** at the Zn K edge in *k*-space (upper) and *R*-space (lower). See Table S7 regarding details of the fit.

Table S7. EXAFS fit parameters for **PtZnTi/SiO$_2$** at the Zn K edge. S0^2=0.90; k range 3.0-11 Å; R range 1-3 Å; k weight 1, 2 and 3; Hanning window dk=1.5; Hanning window dr=0.1; R factor 0.018. Values without errors are fixed.

Path	N	R, Å	σ^2, Å^2	ΔE$_0$, eV
Zn-O	1.5 +/- 0.8	1.92 +/- 0.11	0.0132 +/- 0.0485	
Zn$_1$-Pt	1.1 +/- 0.8	2.49		1.2
Zn$_2$-Pt	1.4 +/- 0.8	2.63	0.0056 +/- 0.0227	
Zn-Zn	0.5 +/- 0.5	2.79 +/- 0.21		

Electron Paramagnetic Resonance Spectroscopy

X-band *ex situ* cw EPR spectra of Ti/SiO_2_H_2, PtTi/SiO_2 and PtZnTi/SiO_2 materials (Figure S39) were recorded on an Elexsys E580 EPR spectrometer (Bruker Biospin, Rheinstetten Germany), equipped with an ESR900 helium flow cryostat (Oxford Instruments, Oxfordshire, UK) and a Super High Q (SHQ) resonator (Bruker Biospin), at 20 K. All samples were evacuated under high vacuum prior to sealing of the capillary. The lock-in amplifier time constant and conversion time were set to 81.92 ms and 163 ms, respectively; the modulation amplitude was set to 0.2 mT (modulation frequency 100 kHz). The spectra were measured in a linear power regime with microwave power attenuation 48 dB (0.003181 mW) All cw EPR spectra were baseline-corrected with an experimental cw EPR spectrum of SiO_2 support material, treated with H_2.

For the estimation of the concentration of Ti^{III} species in the studied materials (Figure S42), a series of cw EPR spectra of $Cu(pic)_2$:$Zn(pic)_2$ reference samples were measured with the same experimental parameters as indicated above, and the intensities of Cu(II) signals, obtained via the double integration of experimental cw EPR spectra, were compared to those of the Ti^{III} species. In order to prepare the reference samples, the desired amount of $Cu(pic)_2$ and $Zn(pic)_2$ complexes (Hpic = 2-picolinic acid) was dissolved in milliQ water, and the water was slowly evaporated on a heat plate at 44 °C. The obtained crystals were grinded carefully, and the weight percentage of Cu was measured with ICP-MS.

X-band *ex situ* echo-detected field sweeps (EDFS) and HYSCORE measurements of Ti/SiO_2_H_2, PtTi/SiO_2 and PtZnTi/SiO_2 materials (Figure S40) were performed at 10 K on a Bruker Elexsys E680 EPR spectrometer, equipped with helium flow cryostat (Oxford Instruments, Oxfordshire,), using a MS3 split-ring resonator (Bruker Biospin). The spectra were recorded at 10 K or 5 K. The EDFS spectra were recorded, using a Hahn echo sequence $\pi/2$-τ-π-τ-echo, with pulse lengths of $t_{\pi/2}$ = 12 ns, t_π = 24 ns, and an interpulse delay τ of 300 ns. For all HYSCORE measurements, the standard 4-pulse sequence $\pi/2$-τ-$\pi/2$-t_1- π-t_2- $\pi/2$- τ-echo with pulse lengths $t_{\pi/2}$ = 16 ns, t_π = 12 ns, and a τ of 192 ns was used. Time steps t_1 and t_2 were set to 16 ns. An eight-step phase cycle was used to remove unwanted echo contributions.

Q-band *ex situ* echo-detected field sweeps (EDFS, Figure S39) of Ti/SiO_2_H_2, PtTi/SiO_2 and PtZnTi/SiO_2 materials were recorded on a homebuilt Q-band spectrometer[10] equipped with a helium flow cryostat (Oxford Instruments, Oxfordshire), using a homebuilt 3 mm

resonator[11]. The spectra were recorded at 5 K using a Hahn echo sequence $\pi/2$-τ-π-τ-echo with pulse lengths of $t_{\pi/2}$ = 12 ns, t_π = 24 ns, and an interpulse delay τ of 400 ns. Simulation of cw and EDFS spectra (Figure S39) was done in Easyspin[12] program. Simulation of HYSCORE spectra (Figure S41) was performed using Hyscorean[13] program.

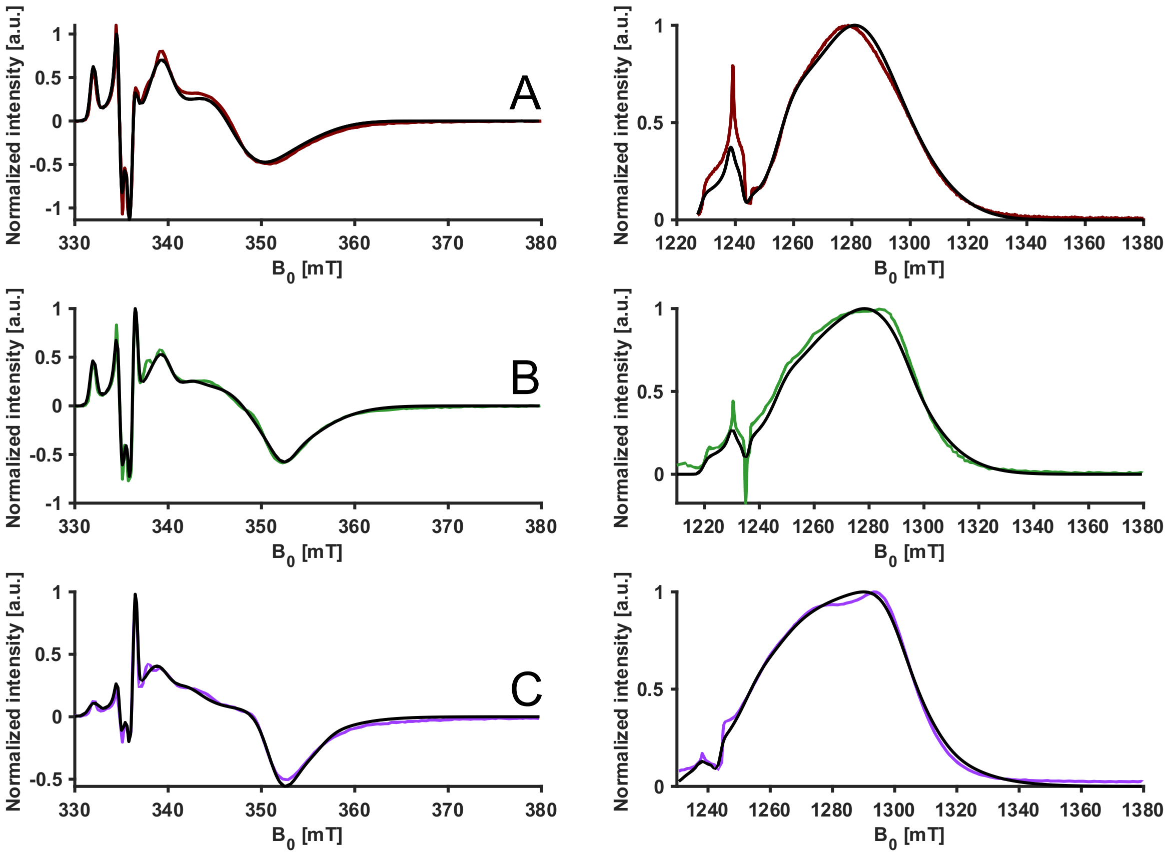

Figure S39. X-band cw (left) and Q-band EDFS (right) spectra of Ti/SiO_2_H_2 (A), $PtTi/SiO_2$ (B) and $PtZnTi/SiO_2$ (C) with the respective simulation in black (see Table S8 for the parameters of the simulation).

Table S8. Parameters of the simulation of experimental X-band cw and Q-band EDFS spectra (Figure S39) of Ti/SiO_2_H_2, PtTi/SiO_2 and PtZnTi/SiO_2 materials. gStrain parameters indicate FWHM Gaussian parameters of inhomogeneous line broadening.

Species	**PtZnTi/SiO_2**	**PtTi/SiO_2**	**Ti/SiO_2_H_2**
Superoxide	*g* = 2.003 2.009 2.026	*g* = 2.003 2.009 2.026	*g* = 2.003 2.009 2.026
	gStrain = 0.003 0.002 0.004	gStrain = 0.003 0.002 0.004	gStrain = 0.003 0.002 0.004
	weight = 0.0146	weight = 0.0447	weight = 0.0646
Ti^{III} (1)	*g* = 1.913 1.922 1.974	*g* = 1.905 1.921 1.973	*g* = 1.905 1.933 1.981
	gStrain = 0.020 0.089 0.031	gStrain = 0.062 0.037 0.027	gStrain = 0.050 0.030 0.015
	weight = 0.85	weight = 0.78	weight = 0.79
Ti^{III} (2)	*g* = 1.891 1.98 1.998	*g* = 1.909 1.977 1.999	*g* = 1.942 1.950 1.999
	gStrain = 0.016 0.015 0.003	gStrain = 0.014 0.013 0.004	gStrain = 0.021 0.054 0.003
	weight = 0.15	weight = 0.22	weight = 0.21

To gain further insights into the EPR active Ti^{III} species, X-band HYSCORE measurements were performed. Figure S40 shows the corresponding X-band EDFS spectra and the HYSCORE spectra for all three materials.

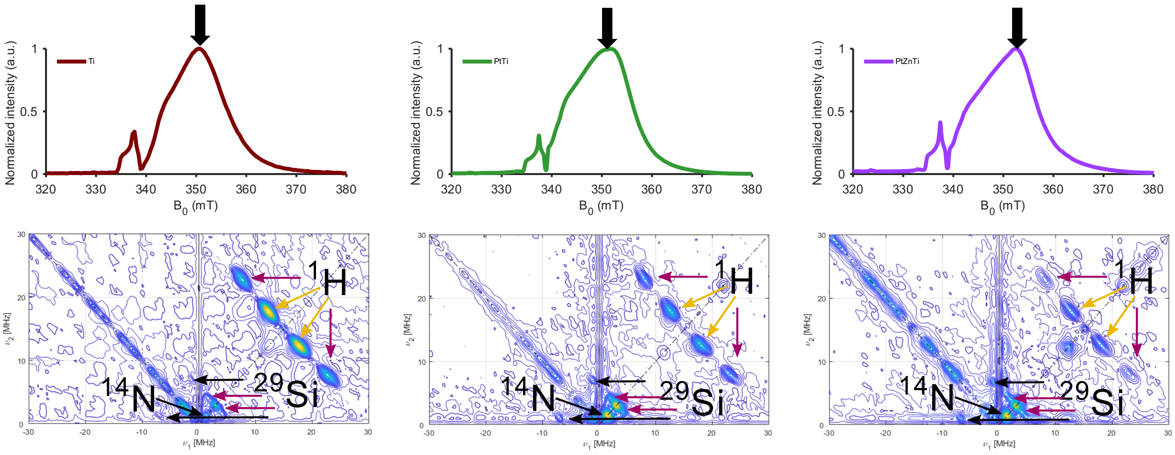

Figure S40. Upper panel: X-band EDFS spectra of Ti/SiO_2_H_2 (dark red), PtTi/SiO_2 (green) and PtZnTi/SiO_2 (purple). Lower panel: the corresponding X-band HYSCORE spectra. Arrows on the EDFS spectra indicate field positions for the corresponding HYSCORE measurements. Different ^{1}H, ^{14}N and ^{29}Si hyperfine couplings are indicated with the arrows of different colors on the corresponding HYSCORE spectra.

The HYSCORE spectra for all three materials are similar to each other. Two different ^{1}H couplings can be distinguished, as well as a coupling to two different Si nuclei from the supporting material (see Figure S40). Additionally, all spectra contain signals centered at

the ^{14}N nuclear frequency. This suggests the presence of hyperfine coupled ^{14}N nuclei, which may arise from nitrogen-containing impurities. N_2 impurities have been reported earlier[14] and are potentially formed upon reaction with gaseous N_2 present as an impurity in the glovebox argon atmosphere.

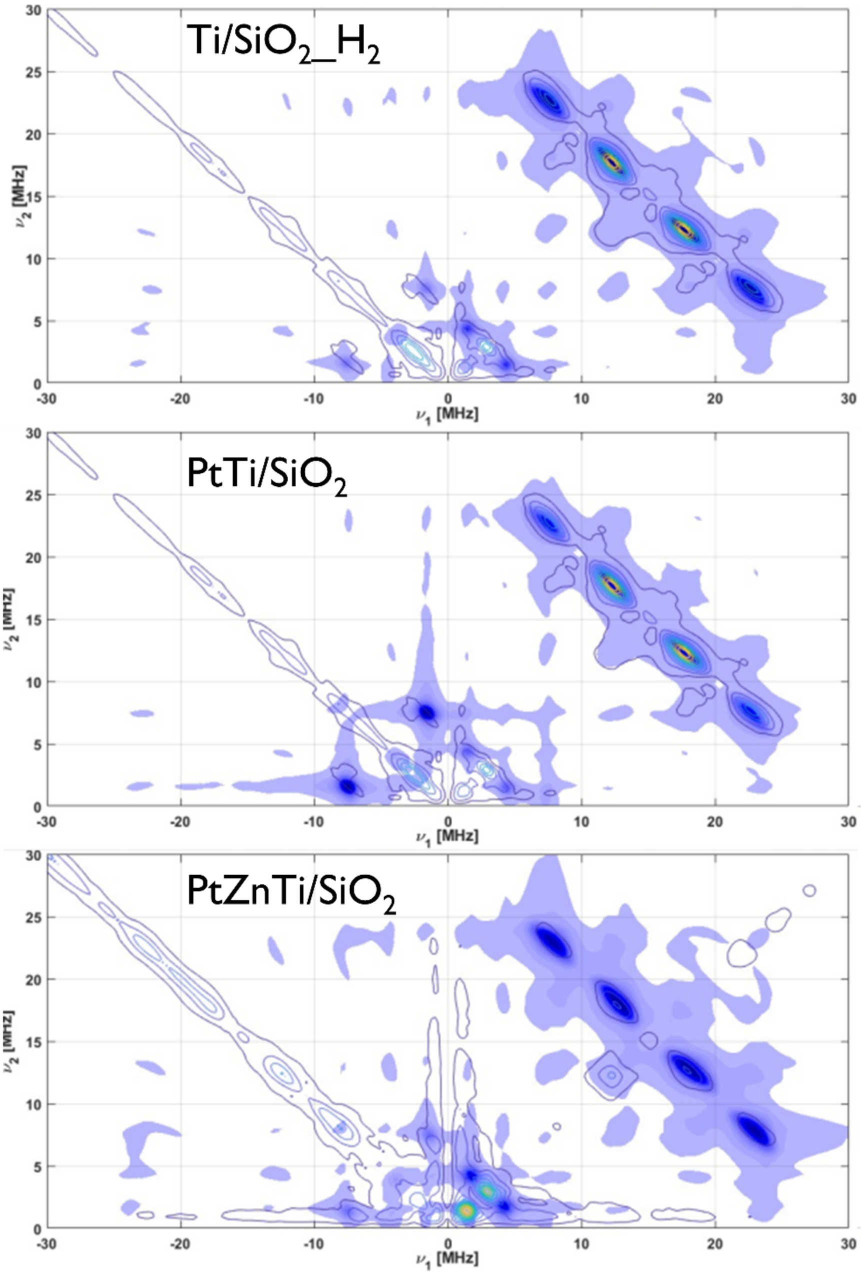

Figure S41. X-band HYSCORE spectra of $Ti/SiO_2\_H_2$, $PtTi/SiO_2$ and $PtZnTi/SiO_2$ materials (contour plots, blue to yellow), together with their spectral simulations (blue patches; see Table S9 for the simulation parameters).

Table S9. Simulation parameters of the X-band HYSCORE shown in Figure S41.

	PtZnTi/SiO2	PtTi/SiO2	Ti/SiO2_H2
^{1}H (MHz)	$A_{\perp}$: 2.2 $A_{\parallel}$:11.9	$A_{\perp}$:2.2 $A_{\parallel}$: 11.9	$A_{\perp}$:2.2 $A_{\parallel}$: 11.9
	$A_{\perp}$: 11.4 $A_{\parallel}$:18.5	$A_{\perp}$:11.4 $A_{\parallel}$: 18.5	$A_{\perp}$:11.4 $A_{\parallel}$: 18.5
^{29}Si (MHz)	$A_{\perp}$: 8.4 $A_{\parallel}$:10.4	$A_{\perp}$:8.4 $A_{\parallel}$: 10.4	$A_{\perp}$:8.9 $A_{\parallel}$: 9.9
	$A_{\perp}$:2.6 $A_{\parallel}$: 3.1	$A_{\perp}$:2.5 $A_{\parallel}$: 3.4	$A_{\perp}$:2.5 $A_{\parallel}$: 3.4

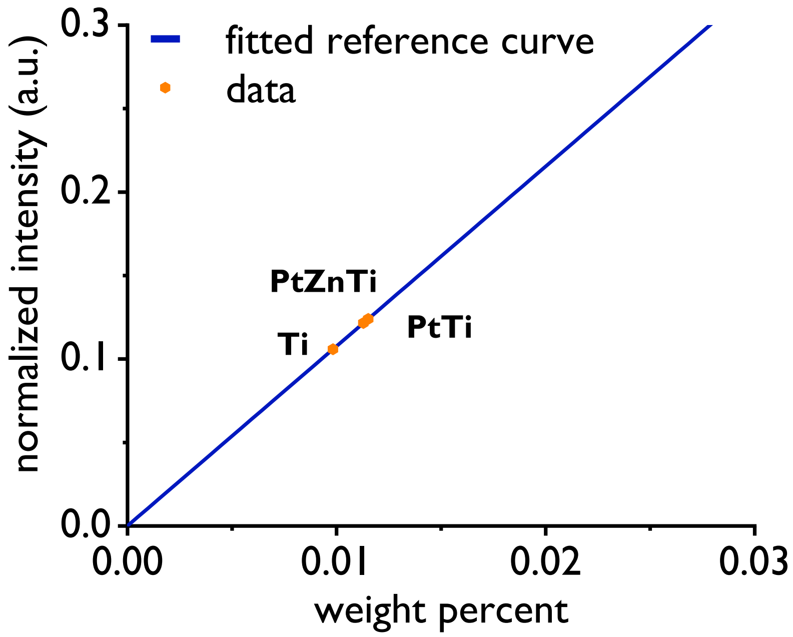

Figure S42. Quantification of Ti^{III} species in Ti/SiO_2_H_2, $PtTi/SiO_2$ and $PtZnTi/SiO_2$ based on a $Cu(pic)_2$:$Zn(pic)_2$ calibration curve and double integration of the cw EPR spectra.

Computational Section

1. Simple cluster models for CO adsorption on SiO_2 supported Pt and TiPt

The models were built by adding Pt atom or a Pt(CO) unit to SiO_2 clusters bearing a Ti^{III} site, a Ti^{IV} site or no Ti site. The following cluster models were considered (*vide infra* for Figures depicting the models):

- $Si_5H_8O_{15}Pt$ – **Pt**,
- $Si_5H_8O_{15}Pt(CO)$ – **Pt(CO)**,
- $Si_3H_3O_9Ti^{III}Pt$ – **Ti^{III}Pt**,
- $Si_3H_3O_9Ti^{III}Pt(CO)$ – **Ti^{III}Pt(CO)**,
- $Si_4H_8O_{15}Ti^{IV}$ –**Ti^{IV}Pt**,

- $Si_4H_8O_{15}Ti^{IV}(CO)$ – **$Ti^{IV}Pt(CO)$**.

DFT calculations on model clusters were performed using the Gaussian 09 (Rev D.01) suite of programs.[15] Structure of minima were optimized using the B3LYP functional.[16–19] First to third period atoms (H, C, O, Si) were described using the Pople basis set 6-31+G(d). The Stuttgart/Cologne group effective-core potential and its associated triple-zeta basis set were used to describe Ti and Pt.[20] Finer than default integration grid and convergence criteria for the optimization were used. The nature of all stationary points was checked by analytical frequency calculations. Basis set superposition error was corrected using the counterpoise method as implemented in Gaussian 09 (Rev D.01). Charge, orbital and spin-population analyses were performed *via* Natural Bond Orbital (NBO) analysis.[21,22] Structures were visualized with VMD and Chemcraft with the following color code for all following Figures of the computational section: Si: blue, C: gray, H: white, Ti: light blue. Pt: light grey, O: red.

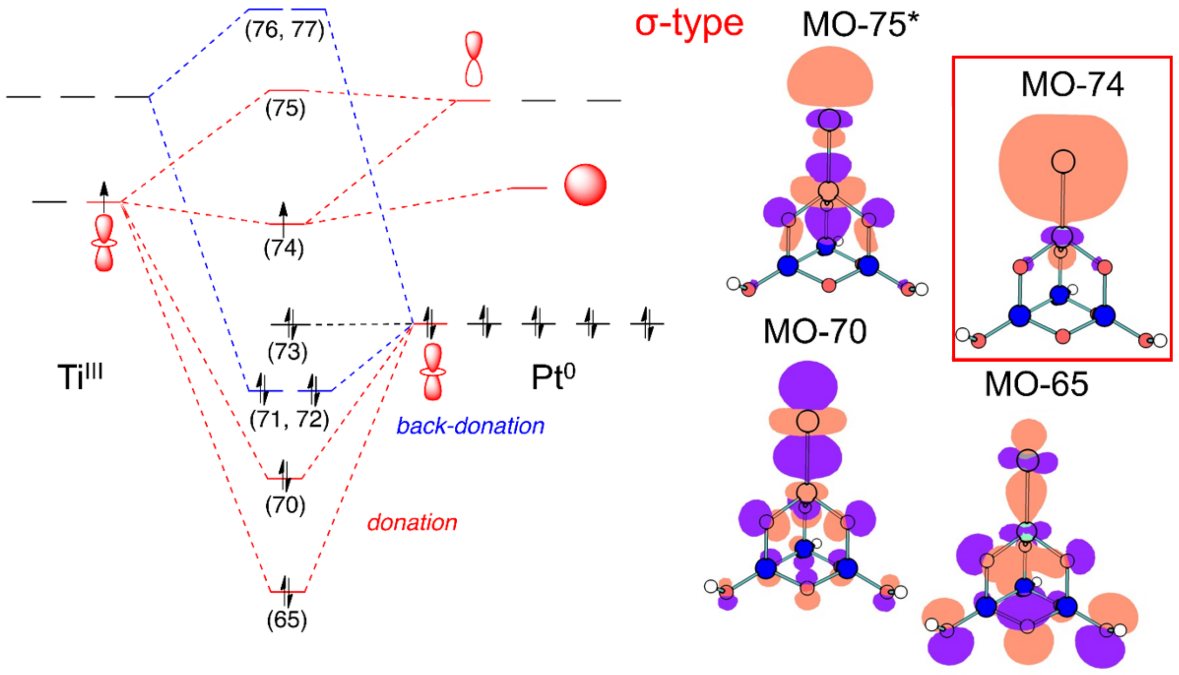

Figure S43. Orbital diagram as obtained from calculations on **$Ti^{III}Pt$**, where Ti^{III} and Pt interact. It can be observed that the SOMO contains the electron formerly located on Ti^{III}, and is now almost fully located on Pt (MO-74). This is also confirmed by spin-population (Pt: 1.04; Ti: -0.04) and charge calculation (Pt: -0.20, Ti +0.15).

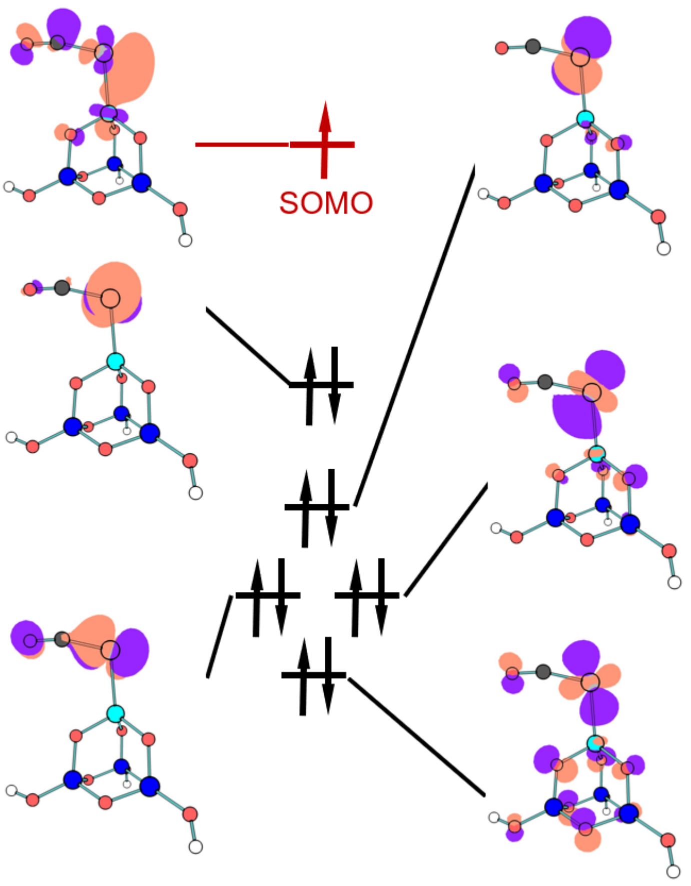

Figure S44. Molecular orbital diagram of the platinum d orbitals and Ti–Pt electron transfer SOMO as obtained from calculations on **Ti^{III}Pt(CO)**.

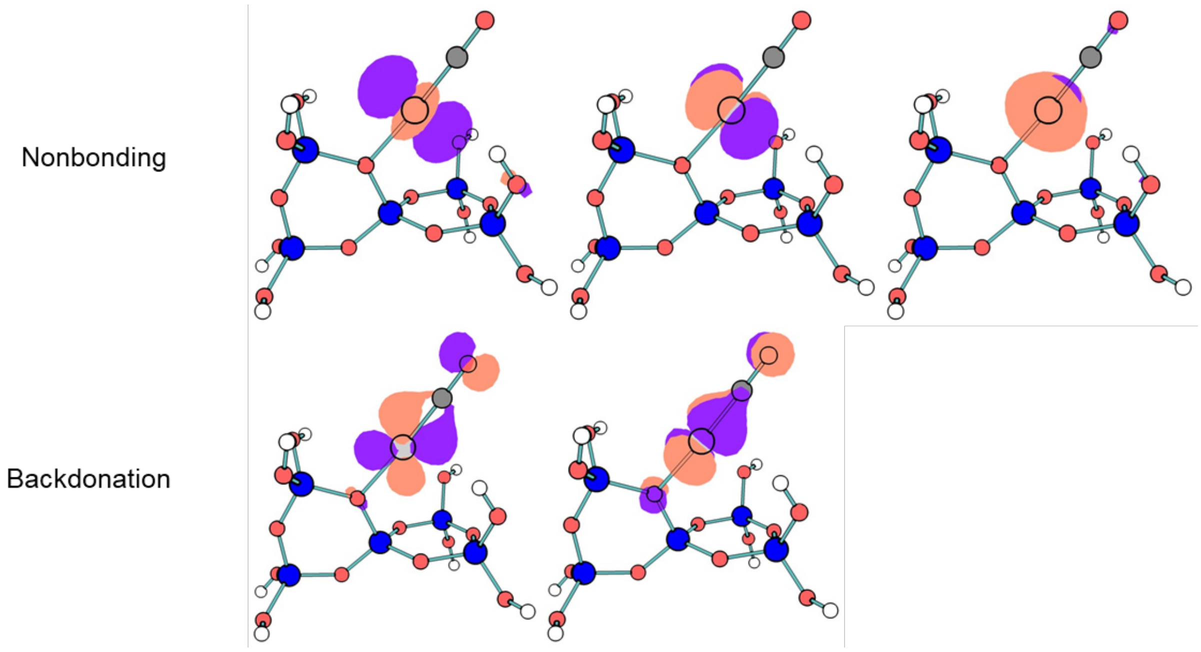

Figure S45. NBOs associated with the d orbitals of Pt for **Pt(CO)**.

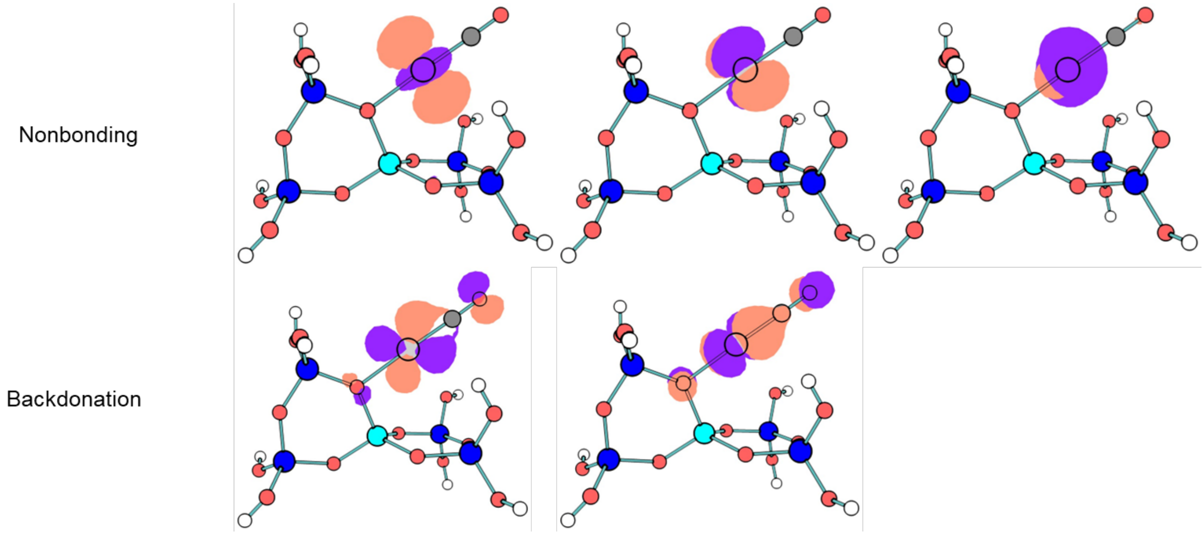

Figure S46. NBOs associated with the d orbitals of Pt for the **Ti^{IV}Pt(CO)**.

Table S10. CO complexation energies (ΔE, kcal mol^{-1}), CO stretching frequencies (v(CO), cm^{-1}), variation of CO stretching frequencies and variation of complexation energies for **Pt(CO)**, **Ti^{IV}Pt(CO)** and **Ti^{III}Pt(CO)**

	**v(CO) (cm^{-1})**	**ΔE (kcal mol^{-1})**
**Pt(CO)**	2006.6	-83.1
**Ti^{IV}Pt(CO)**	2005.2	-80.2
**Ti^{III}Pt(CO)**	1968.0	-35.5
**Δ[Pt(CO)–Ti^{IV}Pt(CO)]**	-1.3	-3.0
**Δ[Pt(CO)–Ti^{III}Pt(CO)]**	-38.6	-47.7

2. Structure optimization on realistic models of Ti-doped amorphous dehydroxylated silica.

Calculations were carried out using CP2K 3.0.[23] Structures were optimized using the revised version of the Perdew–Burke-Ernzerhof GGA functional[24,25] in conjunction with double-ζ MOLOPT basis set[26,27] and Goedecker-Teter-Hutter pseudopotentials[28] on all atoms. The D3 empirical dispersion correction[29] was employed with the damping function of Becke and Johnson.[30] The model of amorphous SiO_2 support previously described by our group was used as starting point.[31] Ti^{III} single sites were created by replacing a Si-OH group with a Ti^{III} center. Ti^{IV} single sites were created by exchanging a Si atom with a Ti^{IV} center. We then considered the interaction of a single Pt^{0} atom with the respective sites. In the case of the most distorted Ti^{IV} sites, Pt can insert into the elongated

Ti-O bond (Figure S48, site II and III), as previously reported for Si-O bonds on dehydroxylated silica.[32,33] Notably, the interaction of a Pt atom with a Ti^{III} site is always significantly more favorable ($\overline{\Delta E}$ = -87.5 kcal mol^{-1}, Figure S50) compared to interaction with Ti^{IV} ($\overline{\Delta E}$ = -59.0 kcal mol^{-1}, very similar to interaction with Ti-absent SiO_2 at $\overline{\Delta E}$ = -54.6 kcal mol^{-1}; see Figure S48) and shows minor changes upon site variation. Overall, these results are in line with those obtained on model clusters **PtTi^{III}** and **PtTi^{IV}**, while evidencing some impact of site variability on the stabilization of Pt atoms by Ti sites.

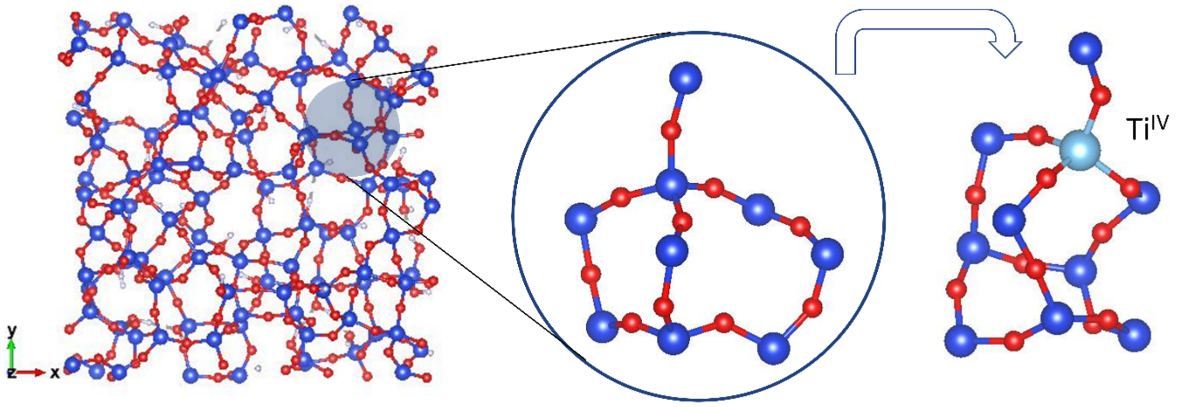

Figure S47. Schematic representation of the design of Ti^{IV} site model for the interaction analysis with a Pt single atom. Different sites were taken from a realistic amorphous SiO_2 model and a Si atom replaced by Ti^{IV}. Only the first coordination sphere is shown for clear representation while the entire surface was used for calculations.

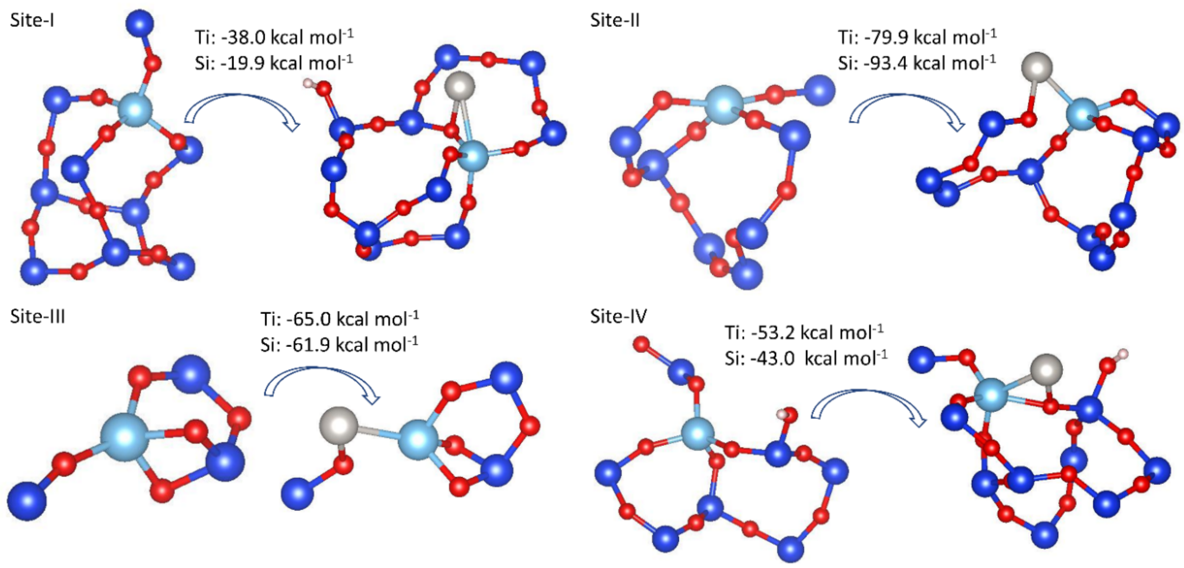

Figure S48. Calculated interaction energies of four different Ti^{IV} sites as well as parent Si containing sites with Pt. The average interaction energy is only slightly higher for the interaction with Ti^{IV} instead of Si. Only the first coordination sphere is shown for clear representation while the entire surface was used for calculations.

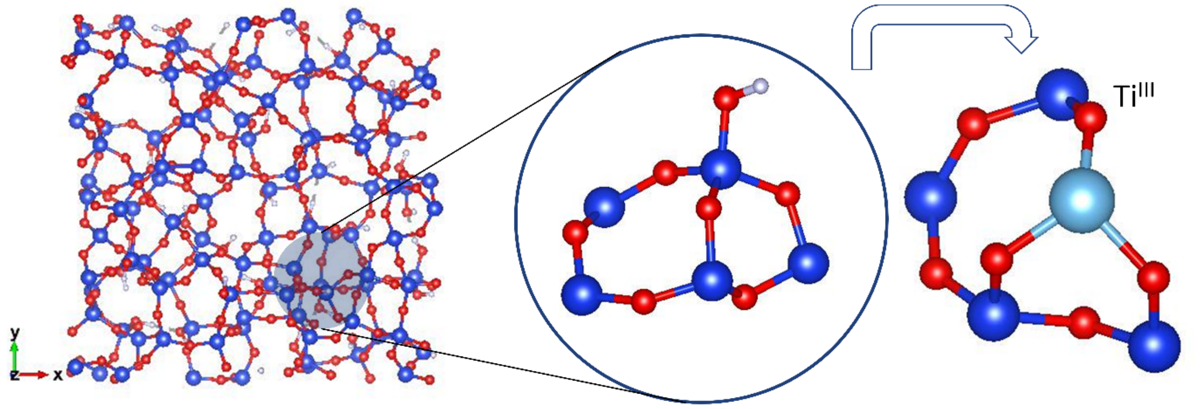

Figure S49. Schematic representation of the design of Ti^{III} site model for the interaction analysis with a Pt single atom. Different sites were taken from a realistic amorphous SiO_2 model and a Si-OH group replaced by Ti^{III}. Only the first coordination sphere is shown for clear representation while the entire surface was used for calculations.

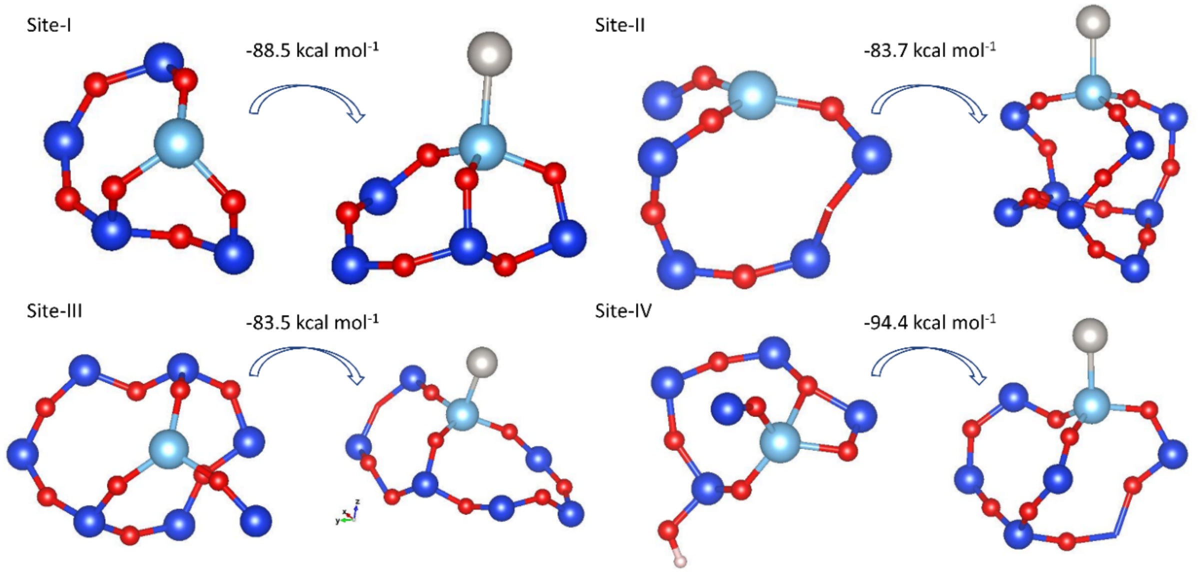

Figure S50. Calculated interaction energies of four different Ti^{III} sites with Pt. The average interaction energy is considerably higher compared to Ti^{IV}/Si interaction with Pt. Only the first coordination sphere is shown for clear representation while the entire surface was used for calculations.

3. Models for Pt nanoparticles supported on $SiO_{2\text{-}700}$ (Pt_{38}/SiO_2) and on Ti-single-site-functionalized $SiO_{2\text{-}700}$ ($Pt_{38}Ti/SiO_2$)

The model was created by adding a Pt_{38} nanoparticle (~1 nm diameter) generated randomly using the Packmol package[34], close to the amorphous silica model with (**$Pt_{38}Ti/SiO_2$**) and without (**Pt_{38}/SiO_2**) Ti single sites. The Ti-functionalized SiO_2 model (**Ti/SiO_2**), was created by adding 4 Ti^{III} sites and 1 Ti^{IV} site (Figure S51). In order to relax the fast degrees of freedom of the system, the structures were equilibrated to 873 K during at least 1 ps with molecular dynamics at the DFT level (AIMD) using the CP2K 7.1 package[23] and optimized, at the level of theory described above. The structures were analyzed using CP2K 7.1[23] to yield Mulliken[35], Hirshfeld[36], Löwdin[37] populations, as well as Density Derived Atomic Point (DDAP)[38] charges, and the Projected Density of States (PDOS).

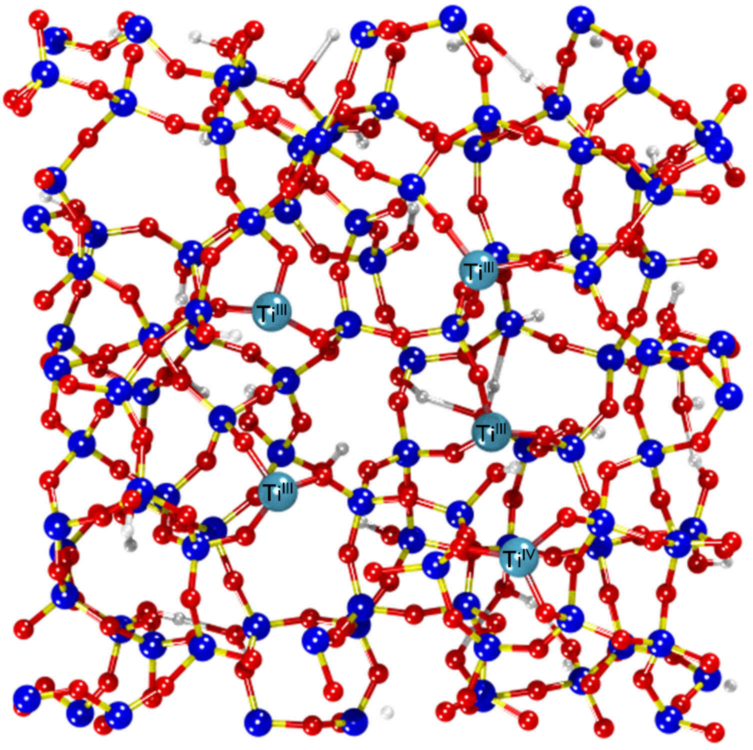

Figure S51. Representation of the designed surface model **Ti/SiO_2** with 4 Ti^{III} sites and 1 Ti^{IV} site.

Table S11. Density derived atomic point charge analysis highlights charge transfer from the Ti sites to the Pt nanoparticle in the $\mathbf{Pt_{38}Ti_5/SiO_2}$ system.

	**Charge (|e-|)**
Ti atoms in $\mathbf{Ti_5/SiO_2}$	+7.1
Ti atoms in $\mathbf{Pt_{38}Ti_5/SiO_2}$	+6.1
**Δ[$\mathbf{Pt_{38}Ti_5/SiO_2}$–$\mathbf{Ti_5/SiO_2}$]**	**-1.0**
Pt atoms in $\mathbf{Pt_{38}/SiO_2}$	-1.0
Pt atoms in $\mathbf{Pt_{38}Ti_5/SiO_2}$	-2.6
**Δ[$\mathbf{Pt_{38}Ti_5/SiO_2}$–$\mathbf{Pt_{38}/SiO_2}$]**	**-1.6**

Table S12. Spin density calculations according to Mulliken, Löwdin and Hirshfeld agree with four independent Ti^{III} sites in the $\mathbf{Ti_5/SiO_2}$ model. The calculation on $\mathbf{Pt_{38}Ti_5/SiO_2}$ and $\mathbf{Pt_{38}/SiO_2}$ converge to an open-shell singlet state, indicating transfer of spin density to Pt.

**Spin of Ti**	**Mulliken**	**Löwdin**	**Hirshfeld**
Total spin density localized on Ti sites	3.88	3.64	3.75
Total spin for $\mathbf{Pt_{38}Ti_5/SiO_2}$		0	
Total spin for $\mathbf{Pt_{38}/SiO_2}$		0	

Catalytic Propane Dehydrogenation

Catalytic tests were performed utilizing a quartz flow reactor designed in our laboratories and a heating/flow setup designed and produced by Micromeritics Instrument Cooperation (PID Eng & Tech). Catalyst samples were loaded into a quartz tubular reactor in an Ar filled glovebox. Prior to exposing the catalyst to flow conditions, a bypass was purged for 20 min with Ar (40 mL/min). The samples were then heated to 550°C utilizing a tubular furnace under a flow of Ar (40 mL/min) and the temperature was maintained until fully stabilized. The gas mixtures containing propane and Ar or other gases/mixtures for the regeneration study (H_2, O_2/Ar) were subsequently mixed and purged through a bypass for 20 min at the desired flow rate prior to contact with the catalyst. Reaction temperatures were maintained utilizing a quartz encased thermocouple maintained in contact with the catalyst dispersed in SiC to yield a total weight of around 2.5 g. The output gas composition was analyzed automatically by a GC with a flame ionization detector (FID) which was programmed to sample the gas stream every 9 minutes throughout the reaction. Gases were purified by passing through a column with molecular sieves and Cu-0226 S (Q5) catalyst (or only molecular sieves in the case of O_2/Ar) prior to introduction to the flow reactor. Propane conversion $X_{C_3H_8}$, selectivity S_i and the carbon balance were calculated according to following equations:

$$X_{C_3H_8} = 1 - \frac{n_{C_3H_8,out}}{n_{C_3H_8,out} + n_{C_3H_6,out} + n_{C_2H_6,out} + n_{C_2H_4,out} + n_{CH_4,out}} \tag{1}$$

$$S_i = \frac{n_{i,out} \Big/ \frac{3}{c_i}}{\sum_i n_{i,out} \Big/ \frac{3}{c_i}} \qquad (c_i = number\ of\ carbon\ atoms\ in\ the\ respective\ molecule\ i) \tag{2}$$

$$carbon\ balance = \frac{n_{C_3H_8,out} + n_{C_3H_6,out} + \frac{n_{C_2H_6,out} \cdot 2}{3} + \frac{n_{C_2H_4,out} \cdot 2}{3} + \frac{n_{CH_4,out}}{3}}{n_{C_3H_8,in}} \tag{3}$$

The catalytic activity of **Ti/SiO₂_H₂**, **PtTi/SiO₂**, **PtZn/SiO₂**, and **PtZnTi/SiO₂** for propane dehydrogenation are summarized in Figures S49-S52. For a typical catalytic test, varying amounts of catalyst (15 mg (**Ti/SiO₂_H₂**), 16.1 mg (**PtTi/SiO₂**), 16.5/77 mg (**PtZn/SiO₂**) and 16.2/75 mg (**PtZnTi/SiO₂**) were used while maintaining identical gas composition and flow rate with a flow of 50 mL/min with 1:4 C_3H_8/Ar (any ratios of gases given are in

v:v). In all cases conversions below equilibrium are achieved. A control experiment using the same experimental setup with just SiC was reported earlier.[9] We were also interested if the difference in stability could arise from a difference in Zn loss for **PtZn/SiO2** and **PtZnTi/SiO2** during the deactivation phase. We therefore performed EA after the long deactivation phases using more catalytic material (see Figure S53). Under the assumption of no loss of Pt, comparing the Pt/Zn wt% ratio after the deactivation phases, it can be calculated that **PtZn/SiO2** contains around 1.14 wt% Zn (1.63 wt% initial) while **PtZnTi/SiO2** contains 1.02 wt% Zn (1.53 wt% initial) after the deactivation phase. This corresponds to 70% (PtZn) and 67% (PtZnTi) of initial Zn for the two cases after deactivation, indicating that it is highly unlikely that the difference in stability arises from a difference in Zn loss. The flow rates, temperatures and pressures used during the catalytic/regeneration tests are as follows:

Long regeneration cycles and standard catalytic tests (if not indicated otherwise):
16.2 mg and 16.3 mg of **PtZn/SiO2** and **PtZnTi/SiO2** were used, respectively. PDH conditions: 550°C, 50 ml/min, 1 barg, 1:4 C_3H_8/Ar for varying times. Oxidation treatment: 500°C, 27 ml/min 5% O_2/Ar, 0.5 barg, 1.5 h. Reduction treatment: 550°C, 20 ml/min H_2, 0.5 barg, 1 h. The setup was flushed with Ar between the PDH and O_2/Ar and between the O_2/Ar and H_2 treatment.

Short regeneration cycles:

31.6 mg and 31.9 mg of material **PtZn/SiO2** and **PtZnTi/SiO2** were used, respectively. PDH conditions: 550°C, 50 ml/min, 1 barg, 1:4 C_3H_8/Ar for 20 min. Oxidation treatment: 500°C, 27 ml/min 5% O_2/Ar, 0.5 barg, 20 min. Reduction treatment: 550°C, 20 ml/min H_2, 0.5 barg, 20 min. The setup was flushed with Ar between the PDH and O_2/Ar and between the O_2/Ar and H_2 treatment.

In the following figures selectivity for all gas phase products is only shown in case of low propene selectivity of the catalyst. The side products in all cases are methane, ethane, ethene and coke in different compositions. Carbon balances close to >95% in all cases except for the quick regeneration cycles where they close 93% for **PtZn/SiO2** and 94% for **PtZnTi/SiO2**. The calculated equilibrium conversion under the employed conditions is around 40%.

The setup was flushed with Ar between the PDH and O_2/Ar and between the O_2/Ar and H_2 treatment.

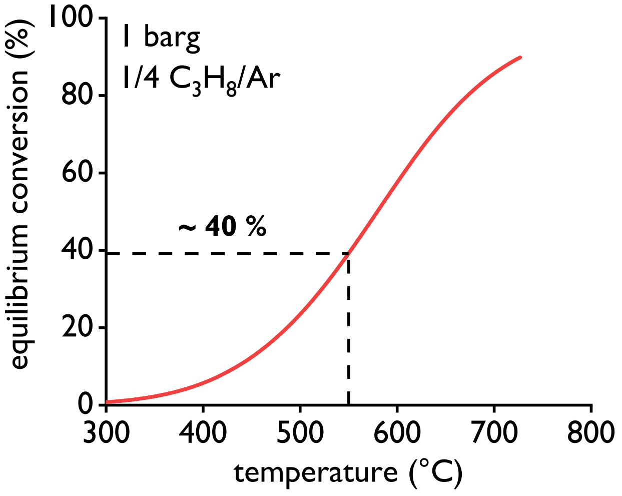

Figure S52. Calculated equilibrium conversion of propane under the PDH conditions used for all catalytic tests.

Mass and Heat transfer limitation analysis. [39] For explanation of symbols refer to Table S13:

$$Mears\ criterion\ (external\ mass\ transport):\quad \frac{-r(obs) \cdot \rho_b \cdot r \cdot n}{k_c \cdot c} < 0.15 \qquad (4)$$

$$external\ heat\ transport:\quad \left|\frac{-\Delta H_r \cdot (-r(obs)) \cdot \rho_b \cdot r \cdot E_a}{h_c \cdot T^2 \cdot R}\right| < 0.15 \qquad (5)$$

$$Weisz - Prater\ criterion\ (internal\ mass\ transport):\quad \frac{-r(obs) \cdot \rho_c \cdot r^2}{D_e \cdot c} \ll 1 \qquad (6)$$

Table S13. List of catalyst process parameters.

**parameter**	**value**
Observed initial consumption rate per mass of catalyst: r(obs) (kmol/(kg_{cat} s))	1.42x10^{-4}
Bulk density of catalyst bed: ρ_b=(1-φ)p_c (kg/m^3)	1860
Porosity: φ[40]	0.38
Catalyst particle radius: r (m)	2.25x10^{-4}
Reaction order: n	1
Mass transfer coefficient: k_c (m/s)	0.268
reactant concentration: c (mol/dm^3)	8.07x10^{-3}
Heat of reaction: ΔH_r (kJ/mol)	124
Activation Energy: E_a (kJ/kmol)[doubled from [41]]	200
Heat transfer coefficient between gas and pellet: h_c (kJ/(m^2 s K))	0.193
Reaction temperature: T (K)	823.15
Ideal gas constant: R (J/(mol K))	8.314
Solid density of catalyst pellet: ρ_c (kg/m^3)	3000
Effective diffusivity: D_e (m^2/s)	1.17x10^{-5}
**Mears number**	**2.75x10^{-2}**
**Heat transport number**	**1.36x10^{-6}**
**Weisz-Prater number**	**2.28x10^{-1}**

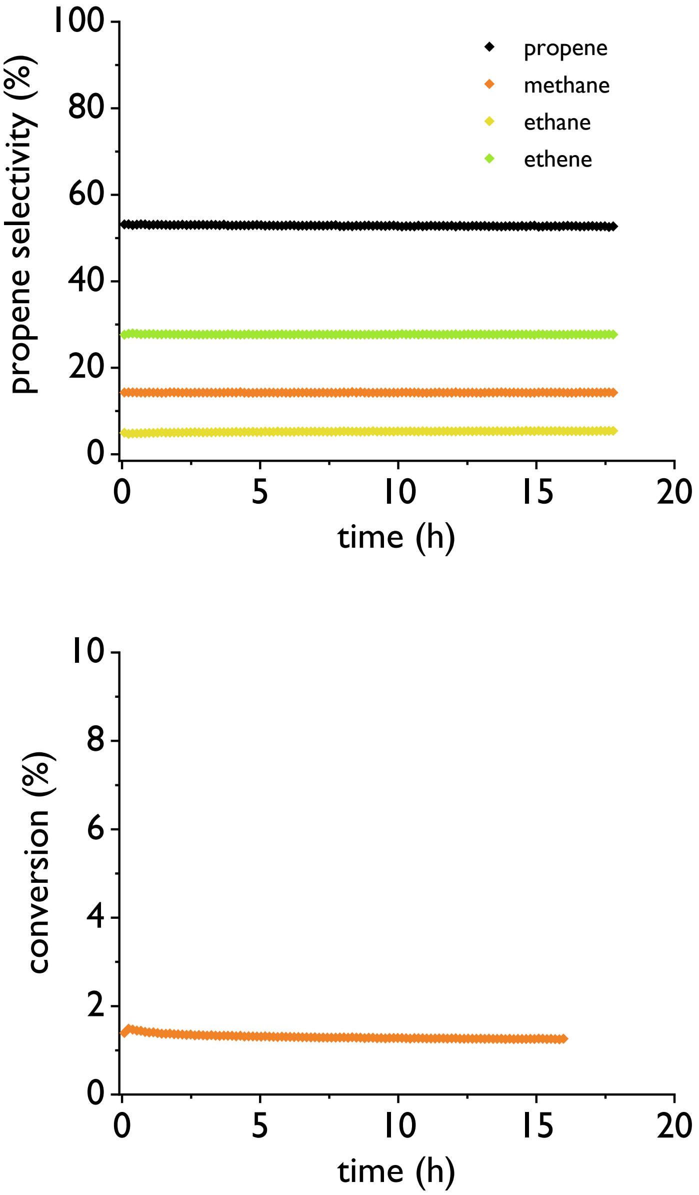

Figure S53. Top: Selectivities of all gas phase products of **Ti/SiO_2_H_2.** Bottom: Propane conversion of **Ti/SiO_2_H_2.** A propene selectivity of around 53 % and conversion of around 1.4 % indicate that this catalyst performs very similar to SiC under the given conditions.

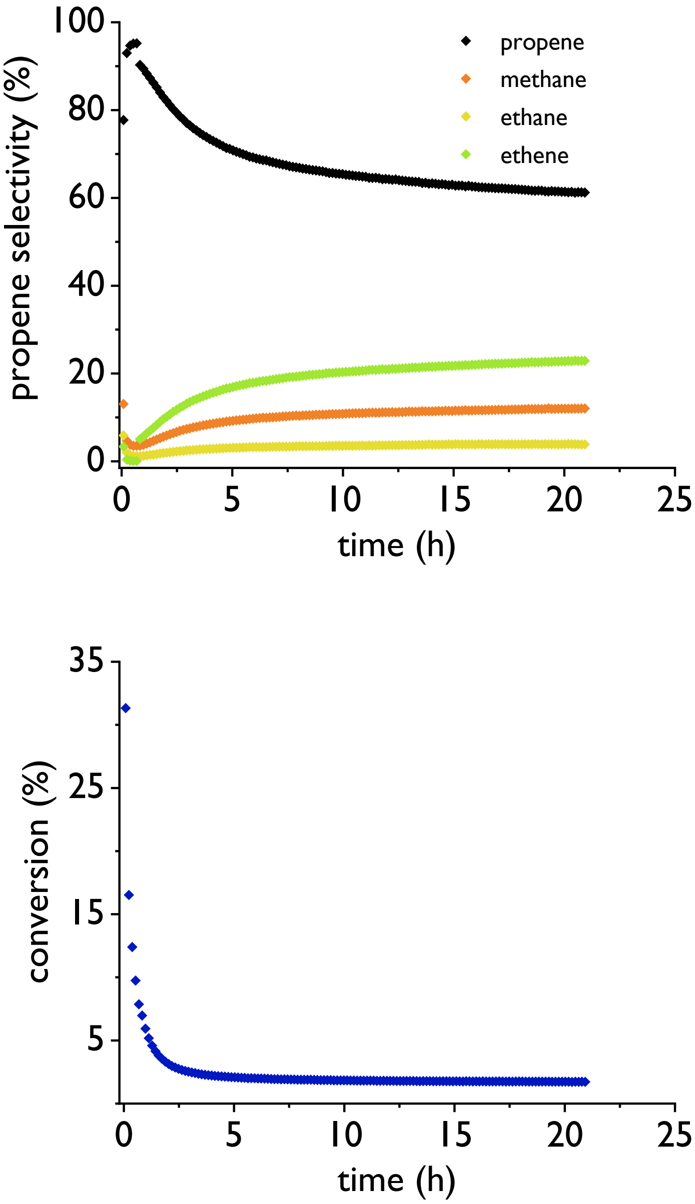

Figure S54. Top: Selectivities of all gas phase products for **PtTi/SiO₂**. Bottom: Propane conversion of **PtZn/SiO₂**.

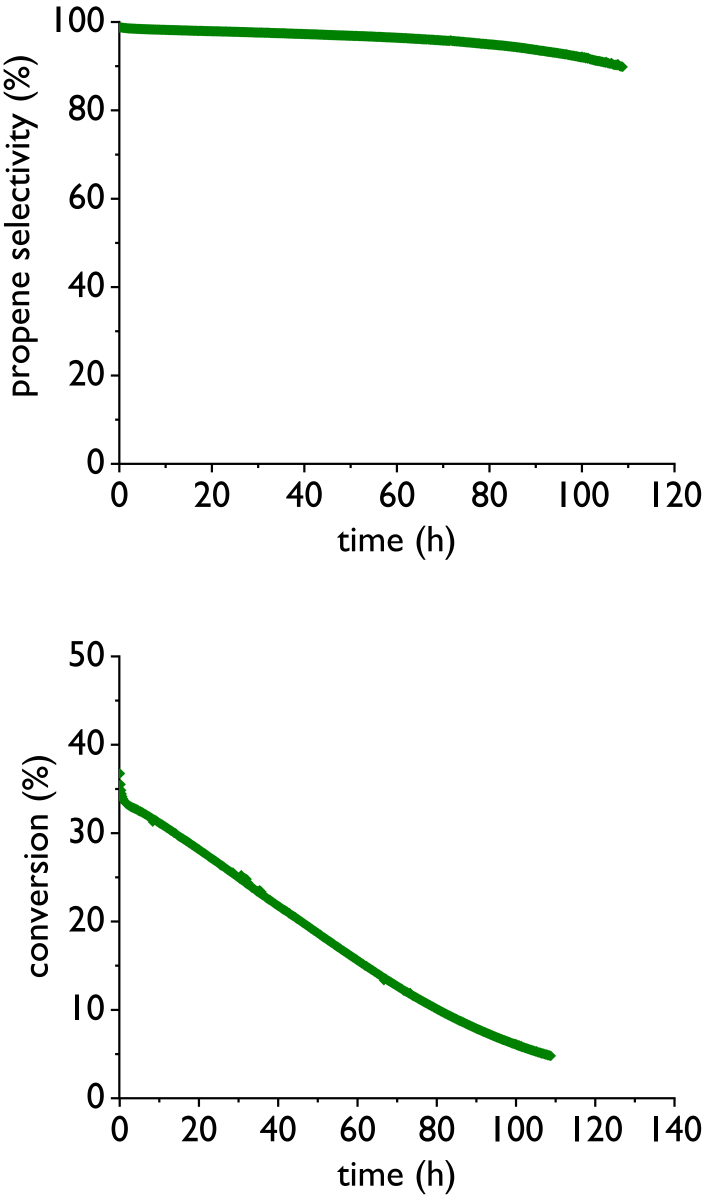

Figure S55. Top: Propene selectivity of **PtZn/SiO$_2$**. Bottom: Propane conversion of **PtZn/SiO$_2$**.

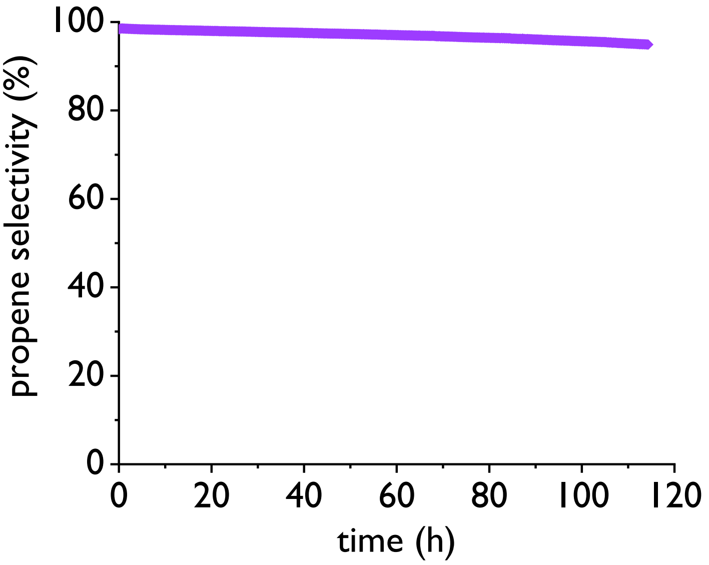

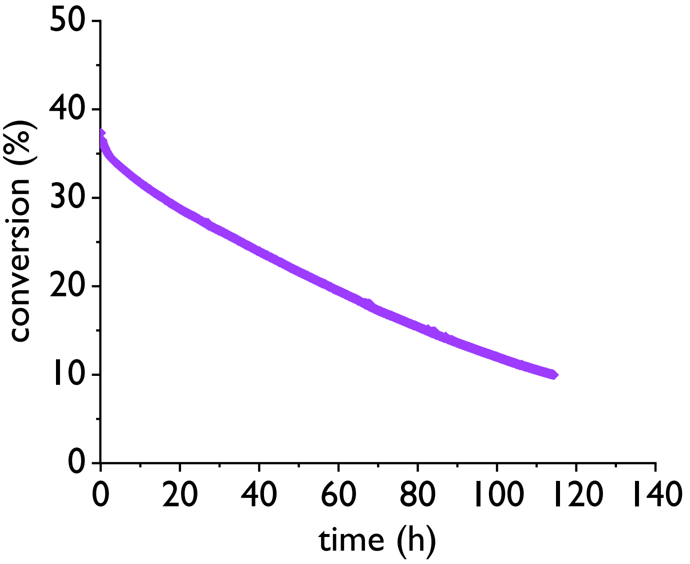

Figure S56. Top: Propene selectivity of **PtZnTi/SiO_2**. Bottom: Propane conversion of **PtZnTi/SiO_2**.

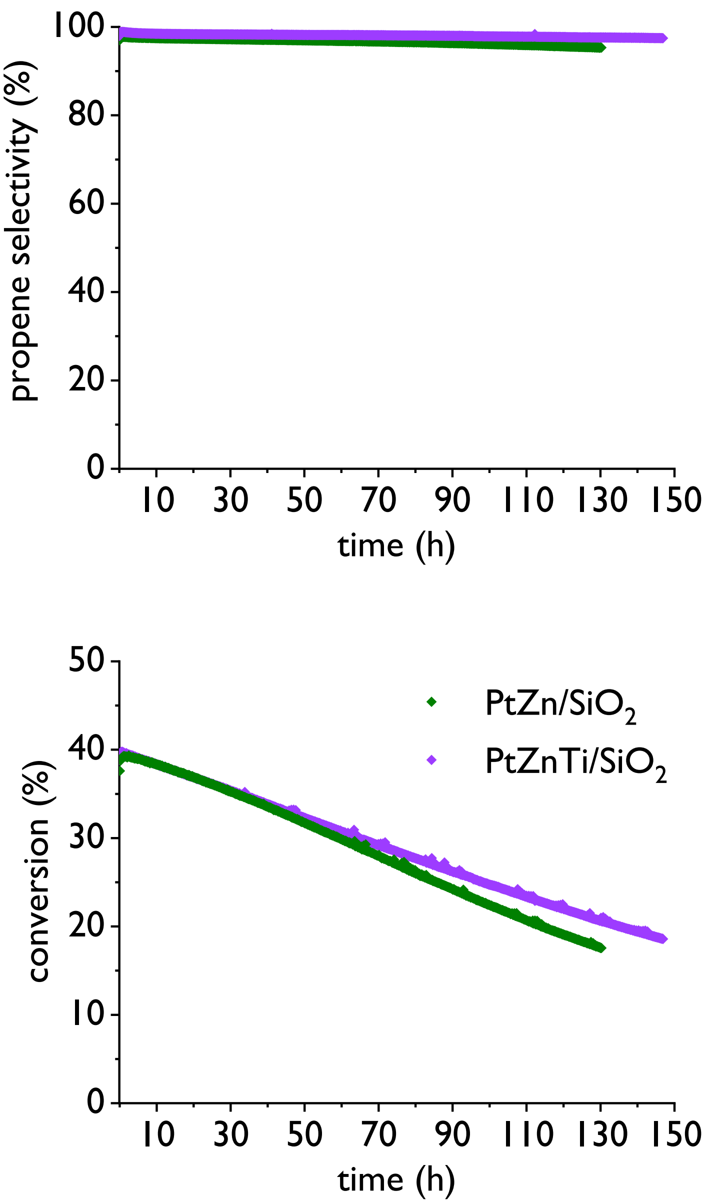

Figure S57. Top: Propene selectivity of **PtZn/SiO2** (green line) and **PtZnTi/SiO2** (purple line). Bottom: Propane conversion of **PtZn/SiO2** (green line, 77 mg, WHSV: 443 h^{-1}) and **PtZnTi/SiO2** (purple line, 75 mg, WHSV: 482 h^{-1}). The stabilizing effect of Ti is still visible but unsurprisingly less pronounced due to the lower WHSV used in this case. The propene selectivity is consistently high for both cases.

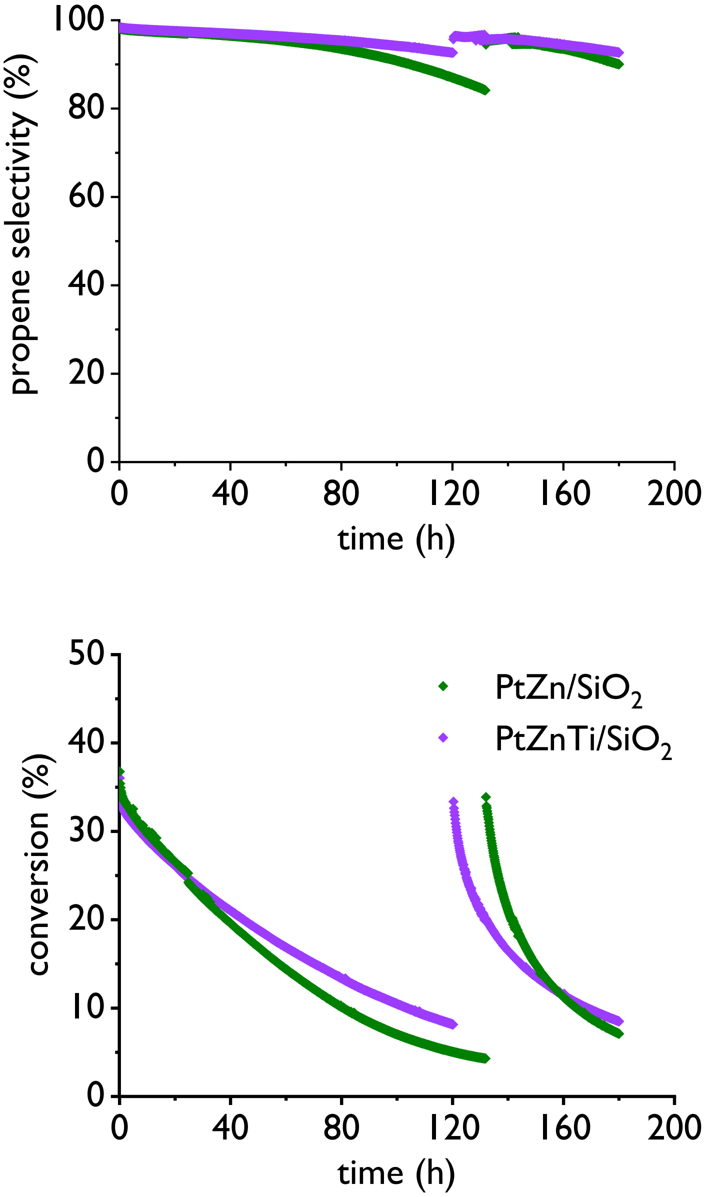

Figure S58. Top: Propene selectivity of **PtZn/SiO₂** (green line) and **PtZnTi/SiO₂** (purple line) under two long deactivation phases with one long regeneration cycle in between. Bottom: Propane conversion of **PtZn/SiO₂** and **PtZnTi/SiO₂** under two long deactivation phases with one long regeneration cycle in between.

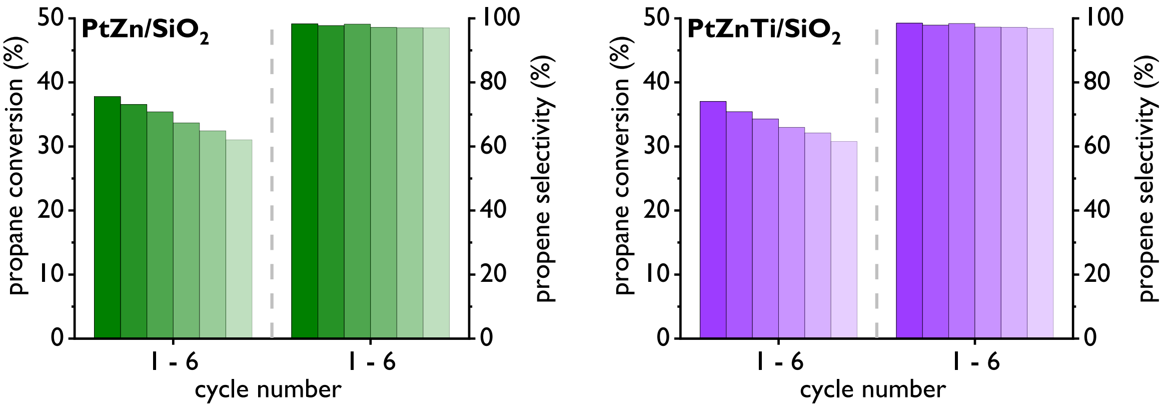

Figure S59. Propane conversion and propene selectivity of **PtZn/SiO_2** (left) and **PtZnTi/SiO_2** (right) during 6 fast, consecutive regeneration cycles, indicating similar robustness of the two materials with 82.0% of initial conversion for **PtZn/SiO_2** and 83.2% for **PtZnTi/SiO_2** after 6 regeneration cycles. The propene selectivity shows a slight decrease over the 6 cycles but stays very high in both cases.

References

[1] K. Su, T. D. Tilley, M. J. Sailor, *J. Am. Chem. Soc.* **1996**, *118*, 3459–3468.

[2] D. A. Ruddy, J. Jarupatrakorn, R. M. Rioux, J. T. Miller, M. J. McMurdo, J. L. McBee, K. A. Tupper, T. D. Tilley, *Chem. Mater.* **2008**, *20*, 6517–6527.

[3] T. Gunji, T. Kasahara, Y. Abe, *J. Sol-Gel Sci. Technol.* **1999**, *13*, 975–979.

[4] G. Noh, E. Lam, J. L. Alfke, K. Larmier, K. Searles, P. Wolf, C. Copéret, *ChemSusChem* **2019**, *12*, 968–972.

[5] P. Laurent, L. Veyre, C. Thieuleux, S. Donet, C. Copéret, *Dalt. Trans.* **2013**, *42*, 238–248.

[6] L. Rochlitz, K. Searles, J. Alfke, D. Zemlyanov, O. V. Safonova, C. Copéret, *Chem. Sci.* **2020**, *11*, 1549–1555.

[7] O. Müller, J. Stötzel, D. Lützenkirchen-Hecht, R. Frahm, *J. Phys. Conf. Ser.* **2013**, *425*, 92010.

[8] B. Ravel, M. Newville, *J. Synchrotron Radiat.* **2005**, *12*, 537–541.

[9] L. Rochlitz, Q. Pessemesse, J. W. A. Fischer, D. Klose, A. H. Clark, M. Plodinec, G. Jeschke, P.-A. Payard, C. Copéret, *J. Am. Chem. Soc.* **2022**, *144*, 13384–13393.

[10] I. Gromov, J. Shane, J. Forrer, R. Rakhmatoullin, Y. Rozentzwaig, A.

Schweiger, *J. Magn. Reson.* **2001**, *149*, 196–203.

[11] R. Tschaggelar, B. Kasumaj, M. G. Santangelo, J. Forrer, P. Leger, H. Dube, F. Diederich, J. Harmer, R. Schuhmann, I. García-Rubio, G. Jeschke, *J. Magn. Reson.* **2009**, *200*, 81–87.

[12] S. Stoll, A. Schweiger, *J. Magn. Reson.* **2006**, *178*, 42–55.

[13] L. Fábregas Ibáñez, J. Soetbeer, D. Klose, M. Tinzl, D. Hilvert, G. Jeschke, *J. Magn. Reson.* **2019**, *307*, 106576.

[14] F. Allouche, D. Klose, C. P. Gordon, A. Ashuiev, M. Wörle, V. Kalendra, V. Mougel, C. Copéret, G. Jeschke, *Angew. Chemie - Int. Ed.* **2018**, *57*, 14533–14537.

[15] M. J. Frisch, G. W. Trucks, H. B. Schlegel, G. E. Scuseria, M. A. Robb, J. R. Cheeseman, G. Scalmani, V. Barone, B. Mennucci, G. A. Petersson, H. Nakatsuji, M. Caricato, X. Li, H. P. Hratchian, A. F. Izmaylov, J. Bloino, G. Zheng, J. L. Sonnenberg, M. Hada, M. Ehara, K. Toyota, R. Fukuda, J. Hasegawa, M. Ishida, T. Nakajima, Y. Honda, O. Kitao, H. Nakai, T. Vreven, J. A. Montgomery Jr., J. E. Peralta, F. Ogliaro, M. Bearpark, J. J. Heyd, E. Brothers, K. N. Kudin, V. N. Staroverov, R. Kobayashi, J. Normand, K. Raghavachari, A. Rendell, J. C. Burant, S. S. Iyengar, J. Tomasi, M. Cossi, N. Rega, J. M. Millam, M. Klene, J. E. Knox, J. B. Cross, V. Bakken, C. Adamo, J. Jaramillo, R. Gomperts, R. E. Stratmann, O. Yazyev, A. J. Austin, R. Cammi, C. Pomelli, J. W. Ochterski, R. L. Martin, K. Morokuma, V. G. Zakrzewski, G. A. Voth, P. Salvador, J. J. Dannenberg, S. Dapprich, A. D. Daniels, Ö. Farkas, J. B. Foresman, J. V Ortiz, J. Cioslowski, D. J. Fox, *Gaussian Inc. Wallingford CT* **2013**.

[16] A. D. Becke, *J. Chem. Phys.* **1993**, *98*, 5648–5656.

[17] C. Lee, W. Yang, R. G. Parr, *Phys. Rev. B* **1988**, *37*, 785–789.

[18] S. H. Vosko, L. Wilk, M. Nusair, *Can. J. Phys.* **1980**, *58*, 1200–1211.

[19] P. J. Stephens, F. J. Devlin, C. F. Chabalowski, M. J. Frisch, *J. Phys. Chem.* **1994**, *98*, 11623–11627.

[20] K. A. Peterson, D. Figgen, M. Dolg, H. Stoll, *J. Chem. Phys.* **2007**, *126*, 124101.

[21] A. E. Reed, R. B. Weinstock, F. Weinhold, *J. Chem. Phys.* **1985**, *83*, 735–746.

[22] E. D. Glendening, J. K. Badenhoop, A. E. Reed, J. E. Carpenter, J. A. Bohmann, C. M. Morales, F. Weinhold, “NBO 5.9,” **2012**.

[23] T. D. Kühne, M. Iannuzzi, M. Del Ben, V. V. Rybkin, P. Seewald, F. Stein, T. Laino, R. Z. Khaliullin, O. Schütt, F. Schiffmann, D. Golze, J. Wilhelm, S. Chulkov, M. H. Bani-Hashemian, V. Weber, U. Borštnik, M. Taillefumier, A. S. Jakobovits, A. Lazzaro, H. Pabst, T. Müller, R. Schade, M. Guidon, S. Andermatt, N. Holmberg, G. K. Schenter, A. Hehn, A. Bussy, F. Belleflamme, G. Tabacchi, A. Glöß, M. Lass, I. Bethune, C. J. Mundy, C. Plessl, M. Watkins, J. VandeVondele, M. Krack, J. Hutter, *J. Chem. Phys.* **2020**, *152*, DOI 10.1063/5.0007045.

[24] J. P. Perdew, K. Burke, M. Ernzerhof, *Phys. Rev. Lett.* **1996**, *77*, 3865–3868.

[25] Y. Zhang, W. Yang, *Phys. Rev. Lett.* **1998**, *80*, 890.

[26] G. Lippert, J. Hutter, M. Parrinello, *Mol. Phys.* **1997**, *92*, 477–488.

[27] J. VandeVondele, J. Hutter, *J. Chem. Phys.* **2007**, *127*, DOI 10.1063/1.2770708.

[28] M. Krack, *Theor. Chem. Acc.* **2005**, *114*, 145–152.

[29] S. Grimme, J. Antony, S. Ehrlich, H. Krieg, *J. Chem. Phys.* **2010**, *132*, DOI 10.1063/1.3382344.

[30] S. Grimme, S. Ehrlich, L. Goerigk, *J. Comput. Chem.* **2011**, *32*, 1456–1465.

[31] A. Comas-Vives, *Phys. Chem. Chem. Phys.* **2016**, *18*, 7475–7482.

[32] P.-A. Payard, L. Rochlitz, K. Searles, L. Foppa, B. Leuthold, O. V. Safonova, A. Comas-Vives, C. Copéret, *JACS Au* **2021**, *1*, 1445–1458.

[33] D. Gioffrè, L. Rochlitz, P.-A. Payard, A. Yakimov, C. Copéret, *Manuscript under Review*, **2022**.

[34] L. Martínez, R. Andrade, E. G. Birgin, J. M. Martínez, *J. Comput. Chem.* **2009**, *30*, 2157–2164.

[35] R. S. Mulliken, *J. Chem. Phys.* **1955**, *23*, 1833.

[36] F. L. Hirshfeld, *Theor. Chim. Acta* **1977**, *44*, 129–138.

[37] P. O. Löwdin, *J. Chem. Phys.* **1950**, *18*, 365–375.

[38] P. E. Blöchl, *J. Chem. Phys.* **1995**, *103*, 7422–7428.

[39] H. S. Fogler, *Elements of Chemical Reaction Engineering*, Prentice Hall, **2006**.

[40] A. S. Pushnov, *Chem. Pet. Eng.* **2006**, *42*, 14–17.

[41] J. T. Miller, V. J. Cybulskis, B. C. Bukowski, H.-T. Tseng, J. R. Gallagher, Z. Wu, E. Wegener, A. J. Kropf, B. Ravel, F. H. Ribeiro, J. Greeley, *ACS Catal.* **2017**, *7*, 4173–4181.
